# An Expedient Total Synthesis of Chivosazole F: an Actin‐Binding Antimitotic Macrolide from the Myxobacterium *Sorangium Cellulosum*

**DOI:** 10.1002/anie.201610636

**Published:** 2016-11-29

**Authors:** Simon Williams, Jialu Jin, S. B. Jennifer Kan, Mungyuen Li, Lisa J. Gibson, Ian Paterson

**Affiliations:** ^1^ University Chemical Laboratory University of Cambridge Lensfield Road Cambridge CB2 1EW UK

**Keywords:** macrocycles, macrolides, polyenes, Stille cross-coupling, total synthesis

## Abstract

A unified strategy for the chemical synthesis of the chivosazoles is described. This strategy is based on two closely related approaches involving the late‐stage installation of the isomerization‐prone (2*Z*,4*E*,6*Z*,8*E*)‐tetraenoate motif, and an expedient fragment‐assembly procedure. The result is a highly convergent total synthesis of chivosazole F through the orchestration of three mild Pd/Cu‐mediated Stille cross‐coupling reactions, including the use of a one‐pot, site‐selective, three‐component process, in combination with controlled installation of the requisite alkene geometry.

The chivosazoles (Scheme 1) are a structurally unique family of bioactive polyene macrolides, which were first isolated by Höfle and Reichenbach from the myxobacterium *Sorangium cellulosum*.^1^ They show highly potent antiproliferative activity against cancer cell lines and have been proposed to function by inhibiting actin polymerization through specific binding to G‐actin, thereby leading to disruption of cytoskeletal dynamics. While actin is increasingly being recognized as a potential target protein in cancer chemotherapy,^2^ the chivosazoles exhibit little structural homology with other known actin‐binding ligands.^3^ As a result, the exact binding site and mode of action of the chivosazoles are likely to be distinct, thus making them an intriguing chemotype for studying the actin cytoskeleton, as well as a promising antimicrofilament lead candidate for drug discovery.

**Scheme 1:**
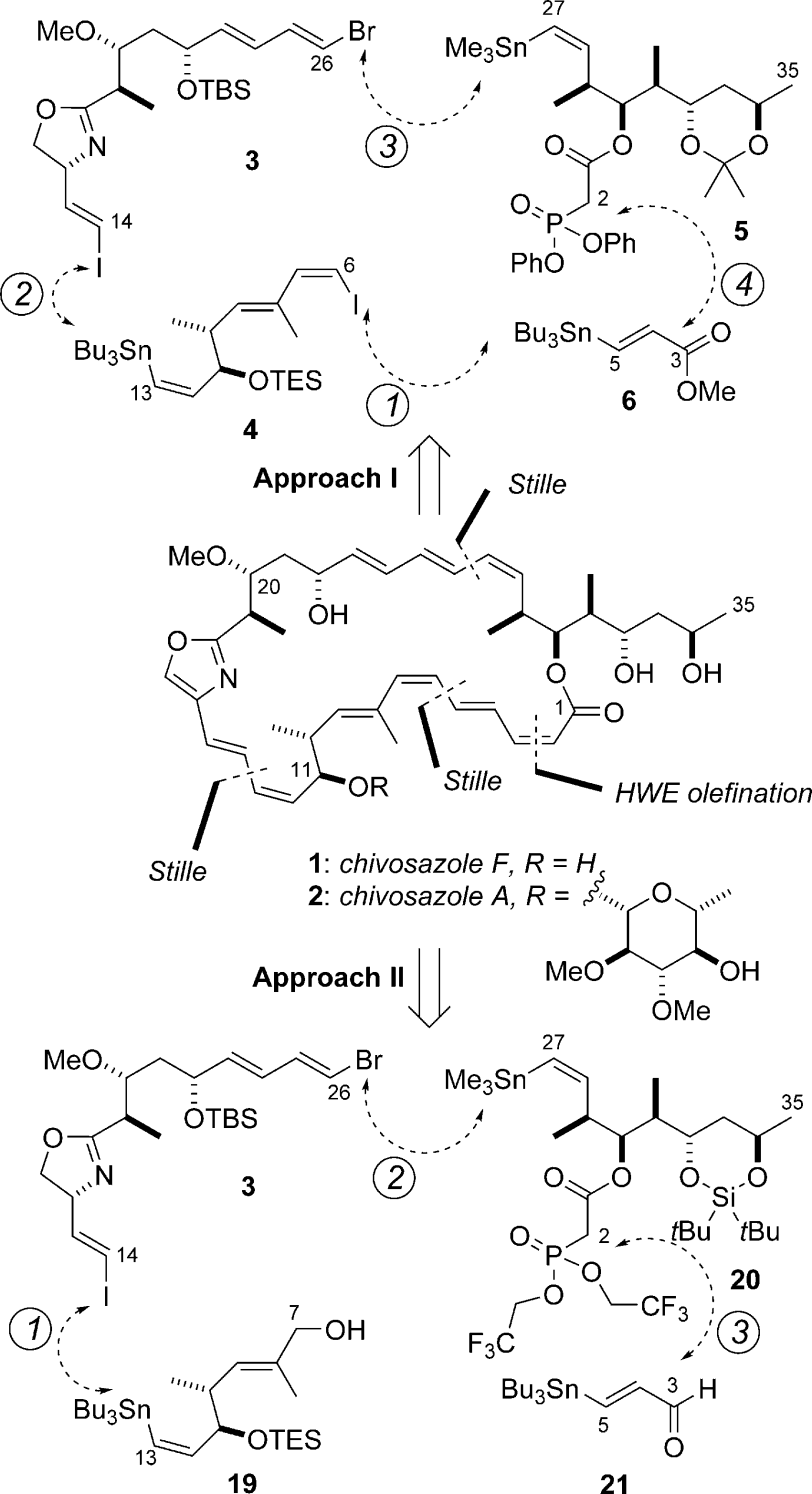
Retrosynthetic analysis of the chivosazoles, highlighting the key fragments for site‐selective Stille cross‐coupling reactions. The same bond disconnections are used in Approaches I and II but their ordering in the forward synthetic sense is different. HWE=Horner–Wadsworth–Emmons, TBS=*tert*‐butyldimethylsilyl, TES=triethylsilyl.

From the perspective of molecular architecture, the chivosazoles display an extraordinary array of features. The signature 31‐membered macrolactone ring features ten stereocenters, an oxazole moiety, and nine alkenes arranged in three distinct conjugated polyene arrays with diagnostic alternating *E*/*Z* geometry.^4^ The six known members of the family differ in the substitution in the 6‐deoxyglucopyranoside appended at C11 and the C20 position, with chivosazole F (**1**) corresponding to the aglycon of chivosazole A (**2**). Despite, or possibly because of, their complex and delicate nature, there have been limited synthetic efforts reported toward the chivosazoles. Notably, the first total synthesis of chivosazole F was achieved by Kalesse's group,5a,5b which served to validate their earlier configurational assignment.^4^ As part of our interest in actin‐binding macrolides,^6^ we also identified the chivosazoles as important target molecules and previously described an efficient aldol‐based construction of two fragments.^7^ By pursuing a unified strategy of site‐selective Stille cross‐coupling reactions to rapidly install the requisite stereodefined polyene motifs under suitably mild conditions, we now report a concise and highly convergent total synthesis of chivosazole F (**1**).

Building on the lessons learned from our earlier synthetic efforts,^7^ we proposed some major retrosynthetic disconnections and key fragments for assembling chivosazole F (Scheme 1). An important consideration was how to best handle the extremely labile and isomerization‐prone (2*Z*,4*E*,6*Z*,8*E*)‐tetraenoate motif contained within the southern hemisphere.5a, 7a With this in mind, we first envisaged that the macrocyclization step would involve an intramolecular Horner–Wadsworth–Emmons‐type olefination to form the tetraenoate system, as outlined in Approach I. This would avoid manipulating a vulnerable open‐chain tetraenoate in favor of a potentially more stable (4*E*,6*Z*,8*E*)‐trienoate moiety. Through judicious tuning of the reactivity of the termini of each of the building blocks (**3**–**6**), it was reasoned that a carefully designed sequence of site‐selective Stille cross‐coupling reactions^8^ would beneficially minimize the need for the manipulation of sensitive intermediates.

Following Approach I, the linchpin fragment **3** was required with a vinyl iodide at C14 and vinyl bromide at C26. A modification to our existing synthetic route (Scheme 2 A),7b starting from the known bromodienal **7^[^** ^9]^ and the chiral methyl ketone **8**,^10^ was used to prepare carboxylic acid **9** with efficient control (>20:1 d.r.) over the installation of the C20 and C22 oxymethine stereocenters. This entailed a tandem boron‐mediated aldol addition^11^, ^12^ [(−)‐Ipc_2_BCl, Et_3_N] and Sm‐promoted reduction^13^ (SmI_2_, EtCHO) sequence, proceeding via the alcohol intermediates **10** and **11** (53 %, 7 steps). The amine **12** 14 was next coupled (EDC, HOBt) with **9** and the resulting amide cyclized to the oxazoline **3** (DAST, 73 %).^15^ At this point, the lack of an electron‐withdrawing substituent on the oxazoline ring presented a potential problem, since the oxidation of unactivated oxazolines to the corresponding oxazoles can be challenging. While this transformation can be accomplished using MnO_2_,^16^ the vinyl iodide in **3** proved incompatible with these conditions, thus dictating that the installation of the oxazole ring should be performed after fragment assembly.

**Scheme 2:**
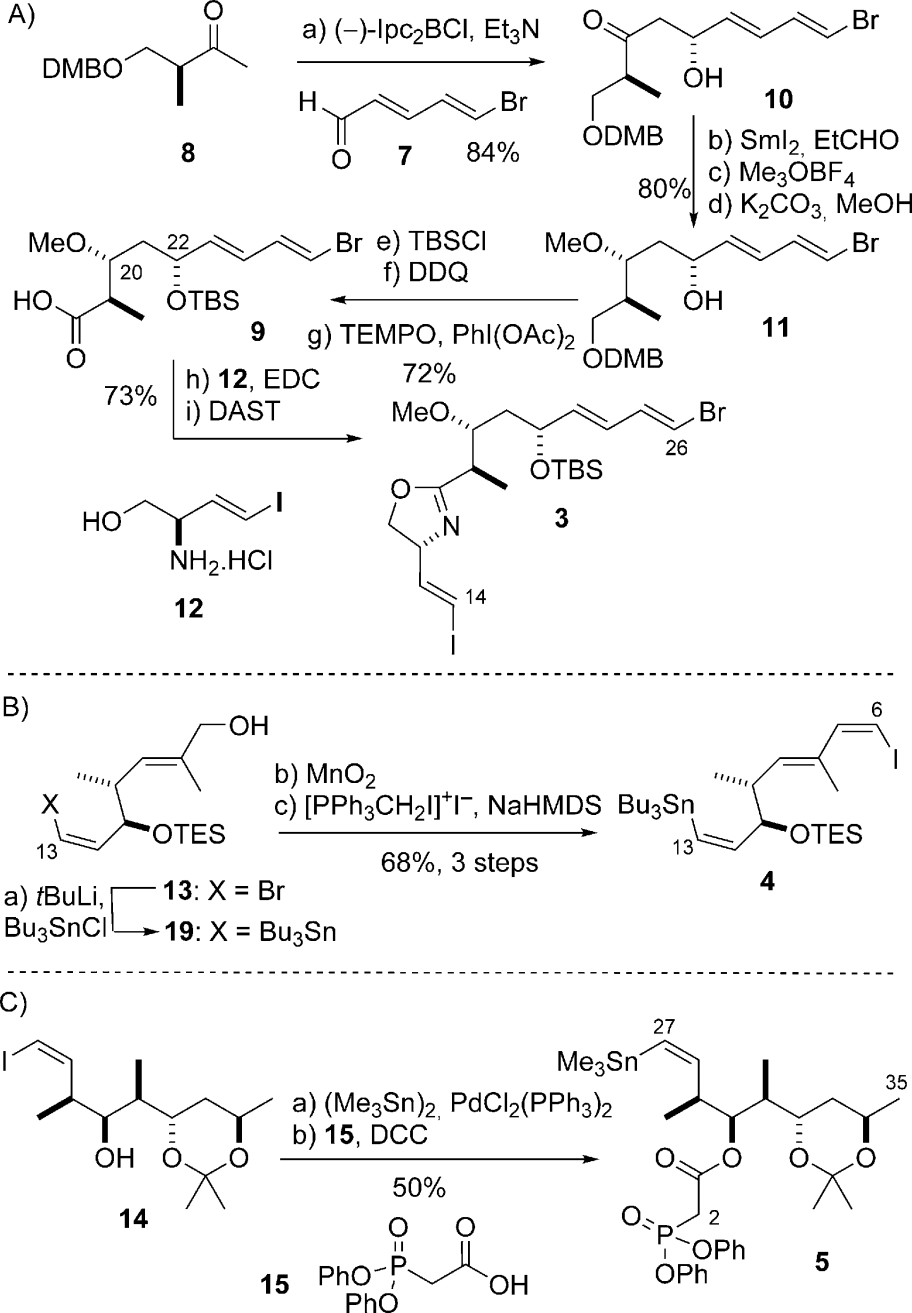
A) Preparation of *bis*‐vinyl halide **3**. Reagents and conditions: a) (−)‐Ipc_2_BCl, Et_3_N, Et_2_O, 0 °C; **7**, −78 °C, 84 %, d.r. >20:1; b) SmI_2_, EtCHO, THF, −20 °C, 96 %, d.r. >20:1; c) Me_3_OBF_4_, Proton Sponge, CH_2_Cl_2_, 0 °C, 92 %; d) K_2_CO_3_, MeOH, 91 %; e) TBSCl, imidazole, CH_2_Cl_2_, 96 %; f) DDQ, pH 7 buffer, CH_2_Cl_2_, 79 %; g) TEMPO, PhI(OAc)_2_, MeCN, H_2_O, 95 %; h) EDC, *i*Pr_2_NEt, HOBt, **12**, CH_2_Cl_2_, 98 %; i) DAST, CH_2_Cl_2_ −78 °C, 75 %. B) Preparation of vinyl stannane **4**. Reagents and conditions: a) *t*BuLi, Et_2_O −78 °C; Bu_3_SnCl, 68 %; b) MnO_2_, CH_2_Cl_2_; c) [PPh_3_CH_2_I]^+^I^−^, NaHMDS, THF, −78 °C, 99 % (2 steps). C) Preparation of vinyl stannane **5**. Reagents and conditions: a) (Me_3_Sn)_2_, PdCl_2_(PPh_3_)_2_, Li_2_CO_3_, THF, 40 °C, 65 %; b) **15**, DCC, CH_2_Cl_2_, 77 %. DAST=diethylaminosulfur trifluoride, DCC=*N*,*N*‐dicyclohexyl carbodiimide, DDQ=2,3‐dichloro‐5,6‐dicyano‐1,4‐benzoquinone, DMB=3,4‐dimethoxybenzyl, EDC=1‐ethyl‐3‐(3‐dimethyl‐aminopropyl) carbodiimide, HMDS=hexamethyldisilazide, HOBt=1‐hydroxybenzotriazole, Ipc=*iso*pinocampheyl, Proton Sponge=1,8‐*bis*(dimethyl‐amino)naphthalene, TEMPO=2,2,6,6‐tetramethylpiperidine 1‐oxyl.

The choice of an iodide at C14 in **3** necessitated the controlled transformation of the C13 bromide in **13** 7a (Scheme 2 B) into the corresponding stannane. This was achieved through lithium–halogen exchange at −78 °C and stannylation (*t*BuLi, Bu_3_SnCl) to afford **19**. Oxidation (MnO_2_) and Stork–Zhao olefination^17^ of the resulting aldehyde then gave the (*Z*)‐vinyl iodide **4** (*Z*/*E*>20:1, 68 %, 3 steps), which corresponds to the second linchpin fragment envisaged in Approach I. A (*Z*)‐vinyl stannane was also required in the third fragment (**5**; Scheme 2 C) and this was readily accessed from **14** 7a using a Pd‐catalyzed stannylation [(Me_3_Sn)_2_, PdCl_2_(PPh_3_)_2_].^18^ An Ando‐type phosphonate^19^ was then appended at the C30 hydroxy group through esterification (**15**, DCC) to afford **5**.

With the three key fragments in hand, the planned Stille cross‐coupling chemistry in Approach I was explored (Scheme 3). Based on the Pd/Cu‐promoted conditions employed in our earlier work,^7^, ^20^ we envisaged the formation, in turn, of the C5−C6, C13−C14, and C26−C27 bonds with the controlled installation of a diene with alternating *E*/*Z* geometry. We elected to initiate this demanding fragment assembly process with the acrylate derivative **6**,^21^ which has a a β‐tributylstannyl substituent. It was anticipated that the (*E*)‐vinyl stannane in **6** would be more reactive for steric reasons than the (*Z*)‐vinyl stannane in **4**, and that the C14 iodide in **3** would be more reactive than the C26 bromide, thereby facilitating the site‐selective construction of the C13−C14 bond. In practice, the first Pd/Cu‐mediated Stille coupling reaction of **4** and **6** was found to be completely selective for the (*E*)‐vinyl stannane in **6** but sensitive to isomerization of the (6*Z*)‐alkene7a in the product **16**. However, this troublesome loss of stereointegrity could be suppressed by the use of *t*Bu_3_P as a ligand for Pd,^22^ which led exclusively to the isolation of **16** (72 %). Under these modified conditions (10 mol % Pd_2_dba_3_, *t*Bu_3_P, CuTC, [Ph_2_PO_2_][NBu_4_], DMF), the second coupling reaction between **16** and the linchpin **3** proceeded smoothly at 0 °C to give **17** (71 %), with complete site selectivity for the C14 iodide over the C26 bromide. Using these same mild conditions, the C27–C35 fragment **5** was finally appended to **17** to afford the desired complex polyene **18** (83 %). The successful orchestration of these three separate fragment couplings, with complete control over the desired alkene geometry, led us to next attempt a one‐pot process to assemble the full backbone of the chivosazoles. In the event, careful monitoring of the reaction and addition of each building block in turn (i. **6**, ii. **4**, iii. **3**, iv. **5**) upon completion of the preceding coupling step, afforded **18** in 56 % yield, which is higher than that achieved when running the sequence as three separate operations (42 % overall). Remarkably, this choreographed reaction sequence efficiently assembles the highly elaborate polyene **18** with alternating *E*/*Z* geometry from four coupling partners in a single flask, under especially mild conditions, and minimizes the need for the isolation and chromatographic purification of sensitive intermediates.

**Scheme 3:**
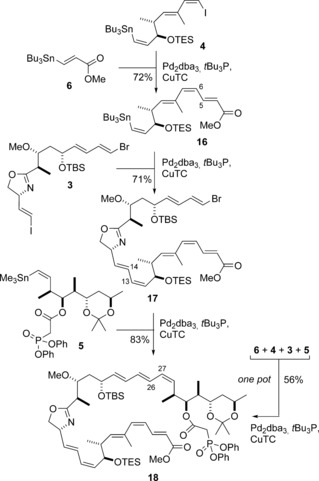
Site‐selective Stille‐type fragment coupling reactions to form the chivosazole backbone by following Approach I. Reagents and conditions: Pd_2_dba_3_, *t*Bu_3_P, CuTC, [Ph_2_PO_2_][NBu_4_], DMF 0 °C. dba=dibenzylidineacetone, DMF=*N*,*N*‐dimethylformamide, TC=thiophene‐2‐carboxylate.

At this advanced stage, it was found that the southern (4*E*,6*Z*,8*E*)‐triene motif in **18** was highly susceptible to alkene isomerization under the conditions examined for elaboration into the chivosazole macrocycle.^23^ While this was frustrating, the feasibility of such an adventurous fragment‐assembly strategy had been validated, and it was considered open to refinement through judicious re‐ordering of the key steps. Conservatively, our revised approach (Approach II; Scheme 1) used the same Stille‐type bond disconnections as in Approach I. This analysis led back to four building blocks (**3**, **19**, **20**, and **21**) and effectively rotated the order of fragment coupling in a clockwise fashion. We now envisaged the use of the alcohol **19** instead of **4** as the southern fragment, which would be coupled to the *bis*‐halide **3** used before. Exploratory studies led us to proceed with a revised north‐eastern fragment **20**, which possesses a silylene protecting group and a Still–Gennari‐type phosphonate.^24^ These modifications were intended to provide improved selectivity for installing the (2*Z*,4*E*)‐dienoate and to facilitate the final global deprotection step.

Adopting this revised plan (Scheme 4), **22** 7b was first converted via **23** into the triol **24**. This was followed by site‐specific silylation (*t*Bu_2_Si(OTf)_2_) of the C32 and C34 hydroxy groups, Pd‐catalysed stannylation [(Me_3_Sn)_2_, Pd(PPh_3_)_2_Cl_2_],^18^ and esterification of **25** (**26**, TCBC) to afford the phosphonate **20**. Returning to the fragment‐assembly phase of the polyene construction (Scheme 5), the two vinyl stannanes **19** and **20** were then sequentially coupled to the *bis*‐halide **3** under Fürstner‐type conditions20a (Pd(PPh_3_)_4_, CuTC, [Ph_2_PO_2_][NBu_4_], DMF) to afford **27** (83 %) and **28** (88 %), respectively. Once again, it was demonstrated that these two Stille coupling reactions could be carried out in a highly efficient, one‐pot tandem process to give **28** (80 %).

**Scheme 4:**
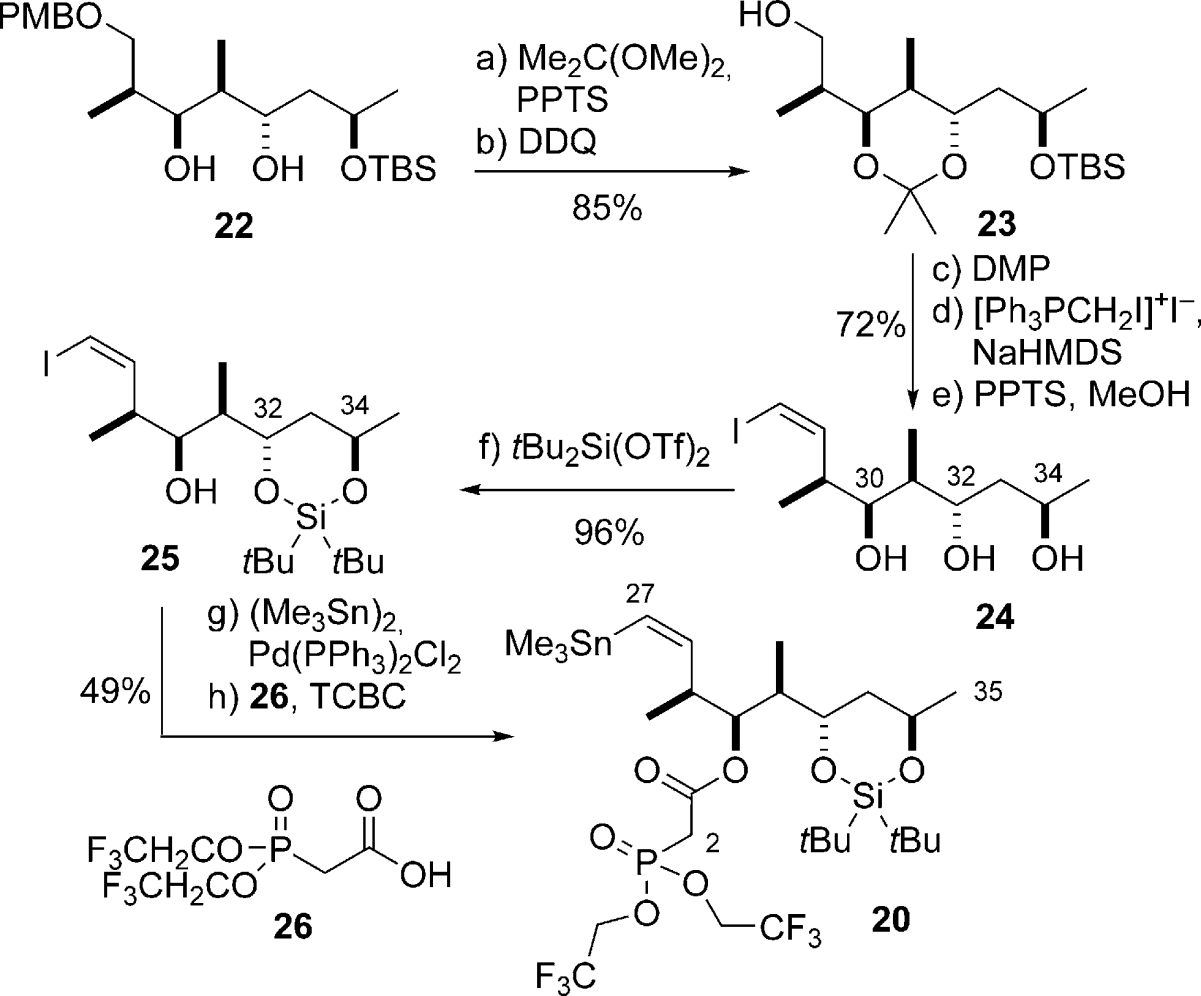
Preparation of the revised C27–C35 fragment **20**. Reagents and conditions: a) Me_2_C(OMe)_2_, PPTS, CH_2_Cl_2_, 93 %; b) DDQ, pH 7 buffer, CH_2_Cl_2_, 91 %; c) DMP, NaHCO_3_, CH_2_Cl_2_, 86 %, d) [PPh_3_CH_2_I]^+^I^−^, NaHMDS, −78 °C, 93 %; e) PPTS, MeOH, 90 %; f) *t*Bu_2_Si(OTf)_2_, 2,6‐lutidine, CH_2_Cl_2_ −78 °C, 96 %; g) (Me_3_Sn)_2_, PdCl_2_(PPh_3_)_2_, Li_2_CO_3_, THF, 40 °C, 65 %; h) **26**, DCC, CH_2_Cl_2_, 76 %. DMAP=*N*,*N*‐dimethyl‐4‐aminopyridine, DMP=Dess–Martin periodinane, PPTS=pyridinium *para*‐toluenesulfonate, Tf=trifluoromethanesulfonyl, TCBC=2,4,6‐trichlorobenzoyl chloride.

**Scheme 5:**
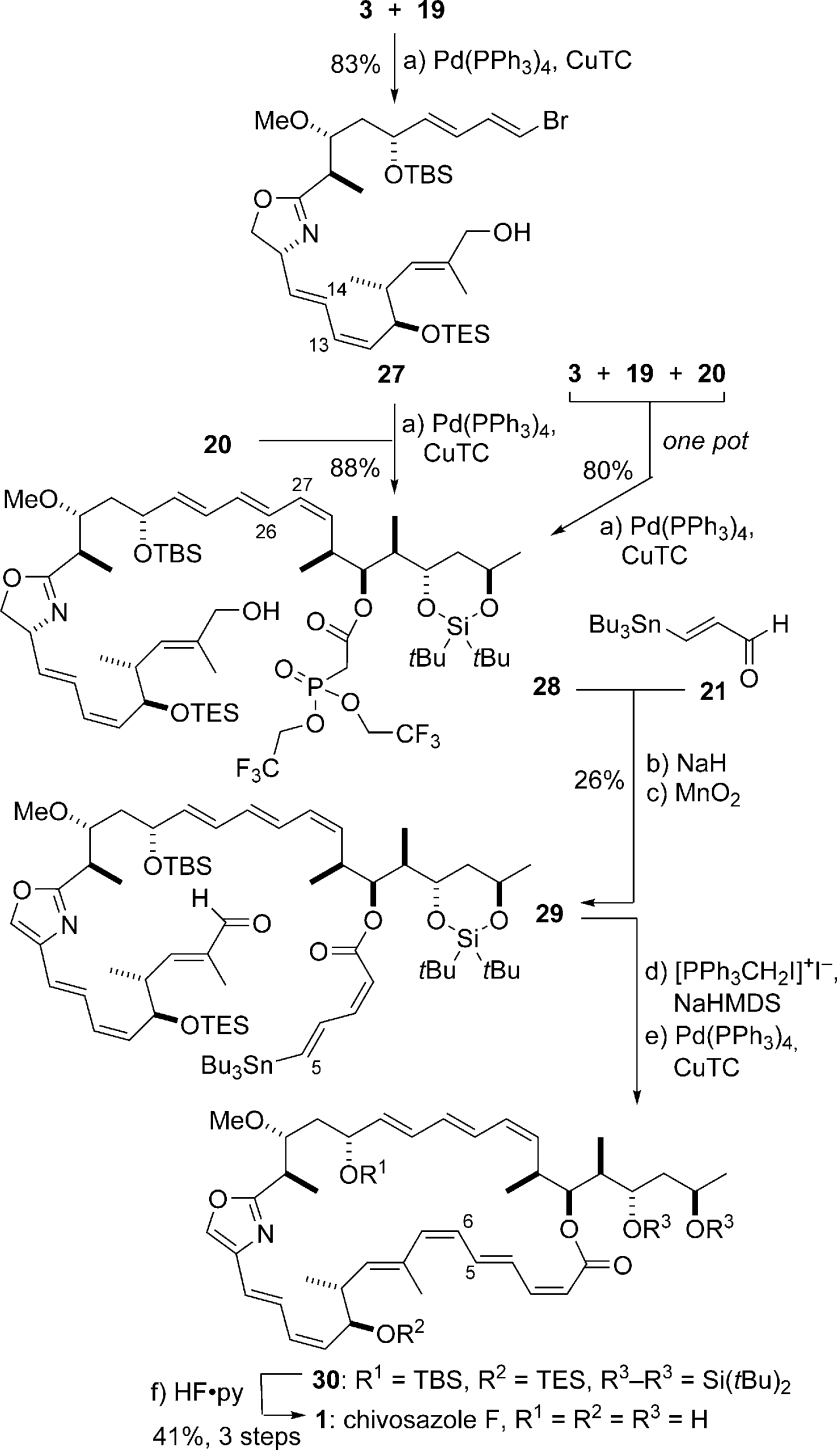
Completion of the total synthesis of chivosazole F (**1**). Reagents and conditions: a) Pd(PPh_3_)_4_, CuTC, [Ph_2_PO_2_][NBu_4_], DMF 0 °C; b) **21**, NaH, THF, −78 °C, 80 %, 2.5:1 *Z*/*E*; c) MnO_2_, PhH, 33 %; d) [PPh_3_CH_2_I]^+^I^−^, NaHMDS, THF, −78 °C; e) Pd(PPh_3_)_4_, CuTC, [Ph_2_PO_2_][NBu_4_], DMF 0 °C; f) HF⋅pyridine, pyridine, THF, 41 % (3 steps).

We were now poised to explore the planned endgame in Approach II (Scheme 5). This first entailed the introduction of the required (2*Z*,4*E*)‐dienoate appendage with a δ‐tributylstannyl substituent in readiness for the penultimate Stille macrocyclization step.^25^ A Still–Gennari HWE olefination of the complex phosphonate **28** with the (*E*)‐enal **21** 26 (NaH, −78 °C)^27^ gave a mixture of two alkenes (2.5:1 *Z*/*E*) in favor of the desired (2*Z*,4*E*)‐dienoate. MnO_2_‐mediated double oxidation then afforded the oxazole aldehyde **29**, and accomplished the challenging aromatization reaction on a delicate substrate. After chromatographic removal of the minor double‐bond isomer to provide the required (2*Z*,4*E*)‐dienoate, Stork–Zhao olefination gave the *seco* precursor for the final intramolecular Stille reaction to close the 31‐membered macrocycle. Gratifyingly, this crucial cyclization step gave the macrolactone **30** with retention of all the polyene stereochemistry. Finally, HF⋅pyridine‐mediated removal of the protecting groups, all silicon based, afforded chivosazole F (**1**). To our satisfaction, all ^1^H and ^13^C NMR spectroscopic data for this synthetic material correlated with those reported for natural chivosazole F.5a

In conclusion, we have completed an expedient total synthesis of the actin‐binding polyene macrolide chivosazole F (20 steps, 2.5 % overall yield) through a highly convergent route based on an orchestrated sequence of Stille cross‐coupling reactions for fragment union and macrocycle formation. Notably, such multi‐component Stille couplings constitute a versatile, mild, and powerful method to rapidly build up molecular complexity in polyketide natural products, and can be further enhanced by judicious telescoping into one‐pot processes.^28^ This route should potentially be applicable to the synthesis of useful quantities of these otherwise scarce anticancer agents, along with designed analogues.

## Conflict of interest

The authors declare no conflict of interest.

*Dedicated to Professor Gilbert Stork on the occasion of his 95* ^*th*^ *birthday*

## Supporting information

As a service to our authors and readers, this journal provides supporting information supplied by the authors. Such materials are peer reviewed and may be re‐organized for online delivery, but are not copy‐edited or typeset. Technical support issues arising from supporting information (other than missing files) should be addressed to the authors.

Supplementary
