# Supplementary material for: An Expedient Total Synthesis of Chivosazole F: an Actin‐Binding Antimitotic Macrolide from the Myxobacterium Sorangium Cellulosum
Source: Angew Chem Int Ed Engl. 2016 Nov 29;56(2):645–9. doi: 10.1002/anie.201610636 (PMC6680201; doi:10.1002/anie.201610636)

Angewandte Chemie
Eine Zeitschrift der Gesellschaft Deutscher Chemiker

Supporting Information

An Expedient Total Synthesis of Chivosazole F: an Actin-Binding Antimitotic Macrolide from the Myxobacterium *Sorangium Cellulosum*

*Simon Williams, Jialu Jin, S. B. Jennifer Kan, Mungyuen Li, Lisa J. Gibson, and Ian Paterson**

anie_201610636_sm_miscellaneous_information.pdf

Supporting Information

Table of Contents

1 General and analytical procedures *1*

2 Detailed experimental procedures *3*

*a. North-eastern fragment **3*** *3*

*b. Southern fragment **4*** *8*

*c. North-western fragment **5*** *9*

*d. Revised north-western fragment **20*** *10*

e. Fragment coupling strategy 1 *13*

f. Fragment coupling strategy 2 *15*

g. Endgame *17*

3 NMR data comparison for natural and synthetic chivosazole A *20*

4 References *21*

5 ^{1}H and ^{13}C NMR spectra. *22*

1. General and analytical procedures

Reactions were carried out under an atmosphere of argon using oven dried glassware and standard techniques for handling air sensitive chemicals, unless the reaction contained aqueous reagents or unless otherwise stated.

Reagents were purified using standard laboratory procedures, benzene, toluene, CH_2Cl_2, and acetonitrile were distilled from CaH_2 and stored under an atmosphere of argon. THF and Et_2O were distilled from potassium or sodium wire / benzophenone ketyl radical and stored under argon. Solvents used for extraction and chromatography were distilled. 2,6-lutidine, di-isopropyl ethylamine, triethylamine and HMPA were distilled from CaH_2 and stored over CaH_2 under an atmosphere of argon. DMF was distilled from $MgSO_4$ and stored over 4Å molecular sieves, DMSO was distilled from and stored over 4Å molecular sieves. Oxallyl chloride was distilled. DDQ was recrystallised from $CHCl_3$, proton sponge was recrystallised from ethanol. All other chemicals were used as received from the manufacturer unless otherwise stated.

Aqueous solutions of ammonium chloride (NH_4Cl), sodium bicarbonate ($NaHCO_3$), sodium thiosulfate ($Na_2S_2O_3$), brine (NaCl) and sodium / potassium (Na/K) tartrate were saturated. Buffer solutions were prepared as directed from stock tablets.

Petroleum ether, boiling point 40 – 60 °C is abbreviated to PE

Purification by flash column chromatography was carried out using Kieselgel 60 (230-400 mesh) and a positive solvent pressure. Preparative thin layer chromatography used Merck Kieselgel 60 F254 plates.

Analytical procedures:

TLC was carried out using Merck Kieselgel 60 F254 plates which were visualised using UV light (254 nm) and stained using potassium permanganate, anisaldehyde or phosphomolybdic acid / $Ce_2(SO_4)_3$ dips.

NMR spectra were recorded using the following machines: Bruker Avance TXO cryoprobe (700 MHz), Avance DCH cryoprobe (500 MHz), Avance 500 BB (500 MHz), Avance TCI cryoprobe (500 MHz), Avance 400 DRX (400 MHz). ^{1}H NMR spectra were recorded at 298 K using an internal deuterium lock for $CDCl_3$ (δ_H = 7.26) or MeOD (δ_H = 3.31 ppm). ^{1}H NMR data are presented as: chemical shift δ (in ppm, relative to TMS (δ_{TMS} = 0), integration, multiplicity (s = singlet, d = doublet, t = triplet, q = quartet, m = multiplet, br = broad,) and coupling constants (*J* in Hz). Signals are assigned according to the numbering scheme for chivosazole F figure 1 unless otherwise indicated. Substituents are denoted by the backbone carbon they are attached to. Assignments have been made based on the 1D data presented along with a range of 2D spectra, and comparison with fully assigned spectra for similar compounds.

^{13}C NMR spectra were recorded at 298 K with proton decoupling and an internal deuterium lock for $CDCl_3$ (δ_C = 77.0 ppm) or MeOD (δ_C = 49.0 ppm). Data are listed by chemical shift (δ / ppm) relative to TMS (δ_{TMS} = 0). Multiplicity and coupling constants are listed where coupling to a heteroatom is observed.

Fourier transform IR spectroscopy (FT-IR) was carried out using a Perkin-Elmer Spectrum-One spectrometer, and spectra were recorded as a thin film. Wavelengths of maximum absorption (υ_{max}) are reported in wavenumbers (cm^{-1}).

Optical rotations were measured using a Perkin-Elmer 241 polarimeter at the sodium D line (589 nm) and are reported as $[\alpha]_D^{20}$, concentration (*c* in g / 100 mL) and solvent.

High resolution mass spectroscopy (HRMS) was carried out by the EPSRC National Mass Spectrometry facility (Swansea, UK) or the departmental Mass spectrometry service (University Chemical Laboratories, Cambridge) using electrospray ionisation (ESI) or atmospheric pressure chemical ionisation (APCI). The parent ion $[M+NH_4]^+$, $[M+Na]^+$ or $[M+H]^+$ is quoted.

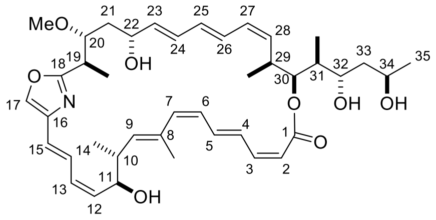

Figure 1 Atom numbering for chivosazole F

2. Detailed experimental procedures

a. North-western fragment 3

Aldol adduct 10

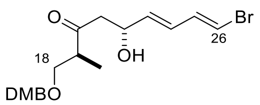

To a solution of (–)-Ipc$_2$BCl (1.86 g, 5.80 mmol) and Et$_3$N (0.93 mL, 6.67 mmol) in Et$_2$O (9.0 mL) at 0 °C was added a solution of ketone **8**[1] (1.30 g, 5.80 mmol) in Et$_2$O (6.0 mL). The reaction mixture was stirred for 30 min then cooled to –78 °C and a solution of aldehyde **7**[2] (0.65 g, 4.06 mmol) in Et$_2$O/CH$_2$Cl$_2$ (1:1, 6.0 mL) was added. The reaction mixture was stirred for 30 min at –78 °C then warmed to –20 °C for 16 h, before being quenched with pH 7 buffer (12 mL) at 0 °C and stirred for 30 min. The reaction mixture was extracted with Et$_2$O (3 x 15 mL), and the combined organic extracts were washed with brine (20 mL) and stirred over silica gel (15 g) for 30 min. The resulting slurry was filtered, the filtrate was concentrated *in vacuo* and purified by flash column chromatography (EtOAc/PE 1:10 → 1:5) to afford aldol adduct **10** (1.23 g, 3.41 mmol, 84%, >95:5 dr) as a yellow oil.

R$_f$ 0.2 (EtOAc/hexane 1:2); $[\alpha]_D^{20}$ = +25.0 (*c* 2.0, CHCl$_3$); **IR** ν_{max} = 3452, 2937, 1709, 1516; **^{1}H NMR** (500 MHz, CDCl$_3$) δ 6.79 − 7.61 (3H, m, ArH), 6.58 (1H, dd, J = 13.4, 10.9 Hz, H25), 6.22 (1H, d, J = 13.4 Hz, H26), 6.09 (1H, dd, J = 15.4, 10.9 Hz, H24), 5.62 (1H, dd, J = 15.4, 5.5 Hz, H23), 4.56 − 4.49 (1H, m, H22), 4.34 (2H, s, OC<u>H</u>$_2$Ar), 3.80 (3H, s, ArOMe), 3.79 (3H, s, ArOMe), 3.52 (1H, dd, J = 9.0, 9.0 Hz, H18a), 3.41 (1H, dd, J = 9.0, 5.2 Hz, H18b), 3.33 (1H, d, J = 3.8 Hz, OH), 2.86 − 2.76 (1H, m, H19), 2.67 − 2.56 (2H, m, H21), 0.99 (3H, d, J = 7.6 Hz, Me19); **^{13}C NMR** (125 MHz, CDCl$_3$) δ 213.4, 149.4, 149.1, 137.1, 135.7, 130.7, 127.7, 120.7, 111.4, 111.3, 109.5 73.6, 72.2, 67.8, 56.3, 56.2, 48.8, 47.2, 13.5; **HRMS** (ES$^+$) calc for $C_{19}H_{29}NBrO_5$ $[M+NH_4]^+$ 430.1224, found 430.1228.

Alcohol S1

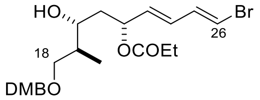

To a solution of propionaldehyde (0.120 mL, 1.61 mmol) in THF (1.5 mL) at –20 °C was added samarium diiodide (1.35 mL, 0.10 M solution in THF, 0.135 mmol) followed by a solution of aldol adduct **10** (120 mg, 0.315 mmol) in THF (5 mL). The reaction mixture was stirred for 45 min, before being quenched with NaHCO$_3$ (5 mL), extracted with Et$_2$O (3 x 5 mL), and the combined organic extracts were dried (Na$_2$SO$_4$) and concentrated *in vacuo*. Purification by flash chromatography (EtOAc/PE 1:7) afforded alcohol **S1** (132 mg, 0.302 mmol, 96%, >95:5 dr) as a colourless oil.

R$_f$ 0.2 (EtOAc/hexane 1:2); $[\alpha]_D^{20}$ = +15.4 (*c* 1.0, CHCl$_3$); **IR** ν_{max} = 3499, 2941, 1733, 1595, 1516; **^{1}H NMR** (500 MHz, CDCl$_3$) δ 6.85 − 6.78 (3H, m, ArH), 6.63 (1H, dd, J = 13.9, 11.1 Hz, H25), 6.31 (1H, d, J = 13.9 Hz, H26), 6.14 (1H, dd, J = 15.6, 10.5 Hz, H24), 5.67 (1H, dd, J = 15.6, 6.6 Hz, H23), 5.56 – 5.49 (1H, m, H22), 4.45 – 4.37 (2H, m, OC<u>H</u>$_2$Ar), 3.85 (3H, s, ArOMe), 3.84 (3H, s, ArOMe), 3.51 (1H, dd, J = 9.1, 4.6 Hz, H18a), 3.51 − 3.46 (1H, m, H20), 3.42 (1H, dd, J = 9.1, 6.9 Hz, H18b), 3.38 (1H, d, J = 4.0 Hz, OH), 2.31 (2H, q, J = 7.6 Hz, CO$_2$C<u>H</u>$_2$CH$_3$), 1.84 − 1.78 (1H, m, H21a), 1.79 − 1.73 (1H, m, H19), 1.59 (1H,

ddd, J = 13.8. 10.5, 3.0 Hz, H21b), 1.11 (3H, t, J = 7.8 Hz, $CO_2CH_2C\underline{H}_3$), 0.89 (3H, d, J = 7.1 Hz, Me19); **^{13}C NMR** (125 MHz, $CDCl_3$) δ 174.5, 149.4, 149.1, 136.9, 133.3, 130.8, 129.4, 120.7, 111.4, 111.3, 110.3, 74.2, 73.7, 71.4, 71.2, 56.3, 56.2, 40.4, 39.1, 28.1, 14.3, 9.5; **HRMS** (ES^+) calc for $C_{22}H_{35}NBrO_6$ $[M+NH_4]^+$ 488.1640, found 488.1642.

Methyl ether S2

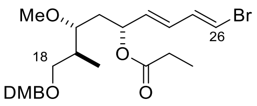

To a solution of alcohol **S1** (150 mg, 0.341 mmol) in CH_2Cl_2 (4.0 mL) at 0 °C was added Proton Sponge™ (0.589 g, 2.75 mmol) followed by trimethyloxonium tetrafluoroborate (0.407 g, 2.75 mmol). The reaction mixture was stirred for 1 h, before being quenched with $NaHCO_3$ (5 mL), filtered through Celite®, and extracted with CH_2Cl_2 (2 x 5 mL). The combined organic extracts were washed with aqueous citric acid (10 mL, 1.0 M), dried ($MgSO_4$) and concentrated *in vacuo*. Purification by flash chromatography (EtOAc/PE 1:7 → 1:4) afforded methyl ether **S2** (142 mg, 0.314 mmol, 92%) as a colourless oil.

R_f 0.4 (EtOAc/hexane 1:2) $[\alpha]_D^{20}$ = +15.3 (*c* 2.0, $CHCl_3$); **IR** ν_{max} = 2941, 1736, 1516; **^{1}H NMR** (500 MHz, $CDCl_3$) δ 6.89 − 6.81 (3H, m, Ar$\underline{H}$), 6.65 (1H, dd, J = 13.8, 11.0 Hz, H25), 6.32 (1H, d, J = 13.8 Hz, H26), 6.15 (1H, dd, J = 15.6, 10.3 Hz, H24), 5.66 (1H, dd, J = 15.6, 6.6 Hz, H23), 5.49 – 5.44 (1H, m, H22), 4.43 (2H, m, $OC\underline{H}_2Ar$), 3.88 (3H, s, ArOMe), 3.87 (3H, s, ArOMe), 3.34 (2H, d, J = 6.4 Hz, H18a, H18b), 3.29 (3H, s, OMe20), 3.29 – 3.27 (1H, m, H20), 2.31 (2H, q, J = 7.6 Hz, $CO_2C\underline{H}_2CH_3$), 2.19 – 2.12 (1H, m, H19), 1.70 (1H, ddd, J = 14.7, 9.9, 2.3 Hz, H21a), 1.59 (1H, ddd, J = 14.7, 9.9, 3.3 Hz, H21b), 1.13 (3H, t, J = 7.5 Hz, $CO_2CH_2C\underline{H}_3$), 0.89 (3H, d, J = 6.9 Hz, Me19); **^{13}C NMR** (125 MHz, $CDCl_3$) δ 173.6, 149.0, 148.6, 136.5, 133.0, 131.0, 129.1, 120.1, 111.0, 110.9, 109.8, 78.2, 73.0, 72.2, 70.9, 57.8, 55.9, 55.8, 35.7, 35.6, 27.8, 12.0, 9.2; **HRMS** (ES^+) calc for $C_{23}H_{33}BrO_6Na$ $[M+Na]^+$ 502.1797, found 502.1799.

Alcohol 11

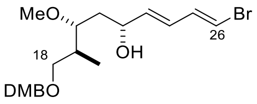

To a solution of ester **S2** (400 mg, 0.882 mmol) in MeOH (7.7 mL) was added K_2CO_3 (173 mg, 1.25 mmol). The reaction mixture was stirred for 16 h, before being quenched with water (8 mL), extracted with CH_2Cl_2 (3 x 10 mL), and the combined organic extracts dried (Na_2SO_4) and concentrated *in vacuo*. Purification by flash chromatography (EtOAc/PE 1:5) yielded alcohol **11** (344 mg, 0.865 mmol, 91%) as a colourless oil.

R_f 0.3 (EtOAc/hexane 1:2); $[\alpha]_D^{20}$ = +18.6 (*c* 1.5, $CHCl_3$); **IR** ν_{max} = 3473, 2937, 1590, 1516; **^{1}H NMR** (400 MHz, $CDCl_3$) δ 6.87 – 6.81 (3H, m, ArH), 6.69 (1H, dd, J = 13.2, 11.0 Hz, H25), 6.28 (1H, d, J = 13.2 Hz, H26), 6.19 (1H, dd, J = 15.3, 11.0 Hz, H24), 5.73 (1H, dd, J = 15.3, 5.2 Hz, H23), 4.41 (2H, s, $OC\underline{H}_2Ar$), 4.40 – 4.33 (1H, m, H22), 3.87 (3H, s, ArOMe), 3.86 (3H, s, ArOMe), 3.57 – 3.51 (1H, m, H20), 3.36 – 3.33 (2H, m, H18a, H18b), 3.32 (3H, s, OMe20), 3.16 (1H, d, J = 4.2 Hz, OH), 2.24 – 2.16 (1H, m, H19), 1.69 (1H, ddd, J = 14.6, 8.6, 3.1 Hz, H21a), 1.56 (1H, ddd, J = 14.6, 8.1, 3.1 Hz, H21b), 0.88 (3H, d, J = 6.9 Hz, Me19); **^{13}C NMR** (100 MHz, $CDCl_3$) δ 149.4, 149.0, 138.0, 137.3, 131.3, 127.1, 120.6, 111.5, 111.3, 108.8, 80.2, 73.5, 72.6, 69.7, 57.5, 56.3, 56.2, 35.9, 35.4, 12.4; **HRMS** (ES^+) calc for $C_{20}H_{33}INO_5$ $[M+NH_4]^+$ 446.1534, found 446.1537.

TBS ether S3

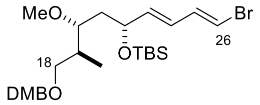

To a solution of alcohol **11** (200 mg, 0.503 mmol) in CH_2Cl_2 (10 mL) was added imidazole (36.0 mg, 0.554 mmol). After stirring for 15 min, TBSCl (82.0 mg, 0.554 mmol) was added. The reaction mixture was stirred for 2 h, before being quenched with $NaHCO_3$ solution (2 mL), extracted with CH_2Cl_2 (3 x 5 mL), and the combined organic extracts dried ($MgSO_4$) and concentrated *in vacuo*. Purification by flash chromatography (EtOAc/PE 1:10 → 1:3) afforded TBS ether **S3** (247 mg, 0.483 mmol, 96%) as a colourless oil.

R$_f$ 0.7 (EtOAc/hexane 1:2); $[\alpha]_D^{20}$ = +20.5 (*c* 1.5, $CHCl_3$); **IR** ν_{max} = 2956, 2850, 1514; **^{1}H NMR** (400 MHz, $CDCl_3$) δ 6.89 – 6.79 (3H, m, ArH), 6.65 (1H, dd, J = 13.9, 10.6 Hz, H25), 6.24 (1H, d, J = 13.9 Hz, H26), 6.05 (1H, dd, J = 15.5, 10.6 Hz, H24), 5.68 (1H, dd, J = 15.5, 6.5 Hz, H23), 4.43 (1H, d, J = 11.6 Hz, $OC\underline{H_a}H_b$ Ar), 4.39 (1H, d, J = 11.6 Hz, $OCH_a\underline{H_b}$ Ar), 4.33 – 4.27 (1H, m, H22), 3.86 (3H, s, Ar(OMe)), 3.85 (3H, s, Ar(OMe)), 3.52 – 3.46 (1H, m, H20), 3.30 (1H, dd, J = 9.1, 6.7 Hz, H18a), 3.30 (3H, s, OMe20), 3.23 (1H, dd, J = 9.0, 6.7 Hz, H18b), 2.27 – 2.19 (1H, m, H19), 1.44 – 1.38 (2H, m, H21a, H21b), 0.88 (3H, d, J = 6.8 Hz, Me19), 0.87 (9H, s, Si*t*<u>Bu</u>Me$_2$), 0.04 (3H, s, SitBu<u>Me$_2$</u>), 0.01 (3H, s, SitBu<u>Me$_2$</u>); **^{13}C NMR** (125 MHz, $CDCl_3$) δ 149.4, 148.9, 139.4, 137.4, 131.5, 126.7, 120.5, 111.3, 111.2, 108.6, 78.2, 73.5, 73.0, 70.0, 57.0, 56.3, 56.2, 39.5, 34.9, 26.3, 18.5, 11.8, –3.5, –4.5; **HRMS** (ES$^+$) calc for $C_{26}H_{47}BrNO_5Si$ $[M+NH_4]^+$ 560.2400, found 560.2401.

Alcohol S4

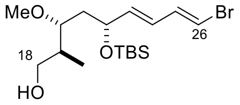

To a solution of DMB ether **S5** (1.00 g, 1.95 mmol) in CH_2Cl_2 (85 mL) at 0 °C was added pH 7 buffer (20 mL) followed by DDQ (0.460 g, 2.03 mmol). The reaction mixture was warmed to rt over 15 min and stirred for a further 1 h, before being quenched with $NaHCO_3$ solution (70 mL). The mixture was extracted with CH_2Cl_2 (3 x 30 mL), the combined organic extracts were dried (Na_2SO_4) and concentrated *in vacuo*. Purification by flash chromatography (EtOAc/PE 1:10) yielded alcohol **S4** (607 mg, 1.54 mmol, 79%) as a yellow oil.

R$_f$ 0.5 (EtOAc/hexane 1:2); $[\alpha]_D^{20}$ = +22.5 (*c* 1.5, $CHCl_3$); **IR** ν_{max} = 3400, 2931, 2865, 1469; **^{1}H NMR** (500 MHz, $CDCl_3$) δ 6.70 (1H, dd, J = 12.0, 12.0 Hz, H25), 6.30 (1H, d, J = 12.0 Hz, H26), 6.09 (1H, dd, J = 15.3, 10.9 Hz, H24), 5.71 (1H, dd, J = 15.3, 6.6 Hz, H23), 4.32 (1H, ddd, J = 6.6, 6.3, 6.3 Hz, H22), 3.67 – 3.60 (1H, m, H18a), 3.59 – 3.52 (1H, m, H18b), 3.48 – 3.43 (1H, m, H20), 3.39 (3H, s, OMe20), 2.28 – 2.24 (1H, m, OH), 2.00 – 1.96 (1H, m, H19), 1.60 – 1.57 (2H, m, H21a, H21b), 0.92 (3H, d, J = 7.0 Hz, Me19), 0.91 (9H, s, Si*t*<u>Bu</u>Me$_2$), 0.09 (3H, s, Si*t*Bu<u>Me$_2$</u>), 0.03 (3H, s, Si*t*Bu<u>Me$_2$</u>); **^{13}C NMR** (125 MHz, $CDCl_3$) δ 138.4, 136.8, 126.7, 108.5, 80.6, 70.1, 65.8, 57.4, 40.2, 37.5, 25.9, 18.1, 12.5, –3.9, –4.8; **HRMS** (ES$^+$) calc for $C_{17}H_{33}BrO_3SiNa$ $[M+Na]^+$ 415.1275, found 415.1275.

Carboxylic acid 9

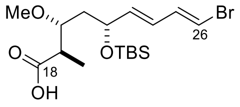

To a solution of alcohol **S4** (100 mg, 0.254 mmol) in $MeCN/H_2O$ (1:1, 5 mL) was added TEMPO (51.3 mg, 0.330 mmol) and $PhI(OAc)_2$ (413 mg, 1.28 mmol). The reaction mixture was stirred for 2 h, before being diluted with $EtOAc/H_2O$ (1:1, 10 mL), extracted with EtOAc (3 x 5 mL) and the combined organic extracts dried (Na_2SO_4) and concentrated *in vacuo*.

Purification by flash chromatography (PE then CH_2Cl_2/MeOH 9:1) yielded acid **9** (98.0 mg, 0.241 mmol, 95%) as a yellow oil.

R_f 0.3 (EtOAc/hexane 1:2); $[\alpha]_D^{20}$ = +16.0 (*c* 1.5, $CHCl_3$); **IR** ν_{max} = 2941, 1726, 1380; **^{1}H NMR** (500 MHz, $CDCl_3$) δ 6.67 (1H, dd, J = 13.8, 10.8 Hz, H25), 6.27 (1H, d, J = 13.8 Hz, H26), 6.08 (1H, dd, J = 15.3, 10.8 Hz, H24), 5.69 (1H, dd, J = 15.3, 6.9 Hz, H23), 4.35 – 4.28 (1H, m, H22), 3.80 – 3.74 (1H, m, H20), 3.38 (3H, s, OMe20), 2.95 – 2.86 (1H, m, H19), 1.63 – 1.48 (2H, m, H21a, H21b), 1.10 (3H, d, J = 7.0 Hz, Me19), 0.89 (9H, s, Si*t*BuMe$_2$), 0.07 (3H, s, Si*t*BuMe$_2$), 0.01 (3H, s, Si*t*BuMe$_2$); **^{13}C NMR** (125 MHz, $CDCl_3$) δ 180.2, 138.7, 137.2, 127.1, 108.9, 78.3, 69.9, 57.5, 41.9, 40.3, 26.3, 18.5, 10.7, −3.5, −4.5; **HRMS** (ES^+) $C_{17}H_{31}BrO_4SiNa$ $[M+Na]^+$ 405.1100, found 405.1102.

Vinyl iodide S5

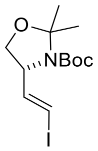

To a solution of DMSO (426 µL, 6.00 mmol) in CH_2Cl_2 (12 mL) at −78 °C was added oxalyl chloride (515 µL, 6.00 mmol). After stirring for 15 min, a solution of Garner's alcohol[3] (925 mg, 4.00 mmol) in CH_2Cl_2 (5.0 mL) was added *via* cannula and the reaction mixture was stirred for a further 1 h. Et_3N (1.67 mL, 12.0 mmol) was added and the reaction mixture was stirred at 0 °C for 30 min, before being quenched with H_2O and extracted with CH_2Cl_2. The organic extracts were dried ($MgSO_4$), concentrated *in vacuo* and the crude aldehyde was used immediately in the subsequent reaction.

To a vigorously stirred solution of $CrCl_2$ (6.65 g, 54.1 mmol) in THF (60 mL) at 0 °C was added the crude aldehyde in THF (30 mL) and the mixture was stirred for 5 min. Iodoform (6.66 g, 16.9 mmol) was added and the reaction mixture was warmed to rt and stirred in the dark for 18 h, before being diluted with H_2O (50 mL) and extracted with Et_2O (3 × 40 mL). The organic extracts were washed with brine, dried ($MgSO_4$) and concentrated *in vacuo*. Purification by flash chromatography (EtOAc/PE/Et_3N 0:1:0.02 → 1:10:0.02) gave vinyl iodide **S5** (791 mg, 2.24 mmol, 56%) as a bright yellow oil.

R_f 0.39 (EtOAc/PE 1:10); $[\alpha]_D^{20}$ = +76.8 (*c* 0.75, $CHCl_3$); **IR** ν_{max} = 2978, 2936, 2873, 1693, 1608; **^{1}H NMR** (500 MHz, $CDCl_3$, 50 °C*) δ_H 6.51 (1H, dd, J = 14.6, 7.6 Hz, H15), 6.31 (1H, brd, J = 14.6 Hz, H14), 4.30 (1H, brs, H16), 4.00 (1H, dd, J = 9.0, 6.3 Hz, H17a), 3.77 (1H, dd, J = 9.0, 2.2 Hz, H17b), 1.60 (3H, s, CMe$_2$), 1.50 (3H, s, CMe$_2$), 1.46 (9H, s, O*t*-Bu); **^{13}C NMR** (125 MHz, $CDCl_3$, 25 °C) δ_C 151.9*, 151.6, 144.3, 143.9*, 94.2, 93.7*, 80.6*, 80.1, 79.0*, 78.2, 67.1, 61.3, 61.1*, 28.4, 27.6*, 26.6, 24.6*, 23.6; **HRMS** (ES^+) calc for $C_{12}H_{20}INO_3$ $[M+H]^+$ 354.0561, found 354.0564.

*Two rotamers are observed for this compound. At 25 °C two sets of carbon signals are resolved but the proton spectrum suffers from significant broadening of peaks. The proton spectrum is thus recorded at elevated temperature to observe a single set of well resolved peaks. Signals for both rotamers are reported for the carbon spectrum with the minor components (approx. 3:2 ratio) denoted *.

Amino alcohol 12

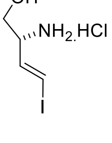

Acetyl chloride (15.0 mL, 212 mmol) was added dropwise to MeOH (150 mL) at 0 °C to prepare a methanolic solution of HCl. A solution of protected amino alcohol **S6** (1.00 g, 2.83 mmol) in MeOH (50 mL) was added dropwise and the reaction stirred at rt for 2 h. Concentration of the reaction mixture *in vacuo* gave the amino alcohol **12** (694 mg, 2.78 mmol, 98%) as a yellow hydrochloride salt.

$[\alpha]_D^{20}$ = +4.4 (*c* 1.43, $CHCl_3$); **IR** ν_{max} = 3354, 2921, 1678, 1598; **^{1}H NMR** (500 MHz, MeOD) δ_H 6.96 (1H, d, J = 15.1 Hz, H14), 6.67 (1H, dd, J = 15.1, 8.1 Hz, H15), 4.89 (3H, brs, OH, NH_2), 3.87 (1H, brs, H16), 3.79 (1H, dd, J = 12.1, 4.1 Hz, H17a), 3.65 (1H, dd, J = 12.1, 7.1 Hz, H17b); **^{13}C NMR** (125 MHz, MeOD) δ_C 140.3, 85.7, 63.1, 58.9; **HRMS** (ES^+) calc for C_4H_8INO $[M+H]^+$ 213.9729, found 213.9726.

Amide S6

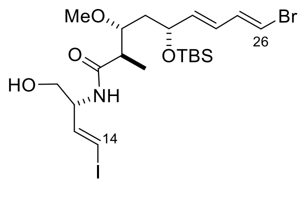

To a solution of acid **9** (200 mg, 0.491 mmol) and hydroxybenzotriazole (72.1 mg, 0.541 mmol) in CH_2Cl_2 (10 mL) at 0 °C was added $^iPr_2NEt$ (90 µL, 0.541 mmol) and EDC (108 µL, 0.541 mmol) and the resulting mixture was stirred for 10 min. Amino alcohol **12** (126 mg, 0.590 mmol) was added and the reaction mixture was allowed to warm to rt and stirred for 1h before being quenched with water (5 mL). The organic layer was separated and the aqueous layer was extracted with CH_2Cl_2 (3 x 5 mL). The combined organic extracts were washed with $NaHCO_3$ (20 mL) and brine (20 mL), dried (Na_2SO_4) and concentrated *in vacuo*. Purification by flash chromatography (EtOAc/PE 1:1) yielded amide **S6** (290 mg, 0.481 mmol, 98%) as a white solid.

R_f 0.2 (EtOAc/hexane 1:3); $[\alpha]_D^{20}$ = −17.6 (*c* 1.5, $CHCl_3$); **IR** ν_{max} = 3307, 2956, 2926, 2870, 1648, 1534; **^{1}H NMR** (400 MHz, $CDCl_3$) δ 6.65 (1H, dd, J = 13.1, 11.0 Hz, H25), 6.54 (1H, dd, J = 14.5, 6.0 Hz, H15), 6.49 (1H, d, J = 8.2 Hz, NH), 6.37 (1H, d, J = 14.5 Hz, H14), 6.28 (1H, d, J = 13.1 Hz, H26), 6.06 (1H, dd, J = 15.7, 11.0 Hz, H24), 5.65 (1H, dd, J = 15.7, 6.0 Hz, H23), 4.54 – 4.49 (1H, m, H16), 4.31 – 4.23 (1H, m, H22), 3.67 – 3.59 (2H, m, H17a, H17b), 3.49 – 3.41 (1H, m, H20), 3.38 (3H, s, OMe20), 2.45 (1H, dt, J = 6.9, 6.6 Hz, H19), 1.69 – 1.51 (2H, m, H21a, H21b), 1.13 (3H, d, J = 6.9 Hz, Me19), 0.87 (9H, s, Si*t*BuMe$_2$), 0.04 (3H, s, Si*t*Bu<u>Me$_2$</u>), 0.02 (3H, s, Si*t*Bu<u>Me$_2$</u>); **^{13}C NMR** (100 MHz, CDCl3) δ 174.9, 143.0, 138.2, 137.1, 127.5, 109.3, 79.9, 79.5, 70.5, 64.6, 59.1, 55.8, 46.0, 41.6, 26.3, 18.6, 14.4, −3.4, −4.3; **HRMS** (ES^+) calc for $C_{21}H_{37}IBrNO_4Si$ $[M+H]^+$ 602.0793, found 602.0779.

***Bis*-halide 3**

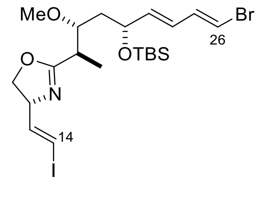

DAST (443 µL, 3.35 mmol) was added dropwise to a solution of amide **S6** (200 mg, 0.332 mmol) in CH_2Cl_2 (4.6 mL) at −78 °C. After stirring for 30 min, the reaction mixture was quenched with K_2CO_3 (695 mg, 5.03 mmol) and allowed to warm to rt, before $NaHCO_3$ was added and the phases separated. The aqueous phase was extracted with CH_2Cl_2 and the combined

organic extracts were washed with brine, dried (Na_2SO_4) and concentrated *in vacuo*. Purification by flash chromatography (EtOAc/PE 1:7) afforded oxazoline **3** (146 mg, 0.249 mmol, 75%) as a pale yellow oil.

R$_f$ 0.7 (1:4 EtOAc/hexane); $[\alpha]_D^{20}$ = +48.4 (*c* 2.0, $CHCl_3$); **IR** ν_{max} = 2928, 2892, 1655, 1469; **^{1}H NMR** (500 MHz, $CDCl_3$) δ 6.64 (1H, dd, J = 13.4, 11.0 Hz, H25), 6.47 (1H, dd, J = 14.5, 6.5 Hz, H15), 6.36 (1H, d, J = 14.5 Hz, H14), 6.24 (1H, d, J = 13.4 Hz, H26), 6.04 (1H, dd, J = 15.6, 11.0 Hz, H24), 5.66 (1H, dd, J = 15.6, 6.4 Hz, H23), 4.56 (1H, ddd, J = 8.3, 6.5, 6.5 Hz, H16), 4.29 – 4.21 (2H, m, H17a, H22), 3.92 (1H, dd, J = 8.3, 8.3 Hz, H17b), 3.70 – 3.65 (1H, m, H20), 3.32 (3H, s, OMe20), 2.95 – 2.88 (1H, m, H19), 1.54 – 1.37 (2H, m, H21a, H21b), 1.09 (3H, d, J = 7.0 Hz, Me19), 0.87 (9H, s, Si*tBu*Me_2), 0.04 (3H, s, Si*t*BuMe_2), −0.02 (3H, s, Si*t*BuMe_2); **^{13}C NMR** (125 MHz, $CDCl_3$) δ 170.1, 145.1, 138.5, 136.9, 126.5, 108.3, 79.1, 77.8, 71.2, 69.7, 69.4, 56.9, 39.5, 34.9, 25.9, 18.1, 10.4, −3.9, −4.9; **HRMS** (ES^+) calc for $C_{21}H_{35}IBrNO_3Si$ [M+H]+ 584.0687, found 584.0681.

b. Southern Fragment 4

Vinyl stannane 19

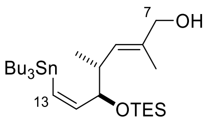

*t*BuLi (1.7 M in hexanes, 16.8 mL, 26.8 mmol) was added dropwise to a solution of vinyl bromide **13**[4] (2.34 g, 6.70 mmol) in Et_2O (120 mL) at −78 °C. After stirring for 5 min, Bu_3SnCl (10.9 mL, 40.2 mmol) was added and the reaction mixture was warmed to rt, quenched with H_2O (100 mL) and extracted with EtOAc (3 × 100 mL). The organic extracts were washed with H_2O (100 mL), dried (Na_2SO_4) and concentrated *in vacuo*. Purification by flash chromatography (1:5:0.02 Et_2O/PE/Et_3N) afforded stannane **19** (2.62 g, 4.55 mmol, 68%) as a colourless oil.

R$_f$ 0.50 (EtOAc/PE 1:5); $[\alpha]_D^{20}$ = +13.4 (*c* 0.98, $CHCl_3$); **IR** ν_{max} = 3380, 2957, 2923, 2875, 1458; **^{1}H NMR** (500 MHz, $CDCl_3$) δ_H 6.42 (1H, dd, J = 13.2, 8.4 Hz, H12), 5.84 (1H, d, J = 13.2 Hz, H13), 5.40 (1H, d, J = 9.8 Hz, H9), 4.00 (2H, br s, H7), 3.77 (1H, dd, J = 8.5, 4.2 Hz, H11), 2.46 (1H, dqd, J = 9.8, 6.9, 4.2 Hz, H10), 1.64 (3H, s, Me8), 1.52 – 1.46 (6H, m, $Sn(CH_2CH_2CH_2CH_3)_3$), 1.36 – 1.29 (6H, m, $Sn(CH_2CH_2CH_2CH_3)_3$), 0.97 (3H, d, J = 6.9 Hz, Me10), 0.97 – 0.88 (24H, m, $Sn(CH_2CH_2CH_2CH_3)_3$, $SiCH_2CH_3$), 0.56 (6H, q, J = 7.8 Hz, $SiCH_2CH_3$); **^{13}C NMR** (125 MHz, $CDCl_3$) δ_C 151.6, 134.4, 128.2, 127.6, 79.8, 69.2, 39.9, 29.2, 27.4, 17.5, 14.1, 13.7, 10.2, 6.9, 5.0; **HRMS** (ES^+) calc for $C_{27}H_{56}O_2Si^{112}Sn$ $[M+H]^+$ 553.3171, found 553.3171.

Vinyl iodide 4

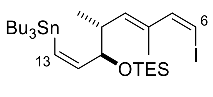

To a solution of alcohol **19** (300 mg, 0.522 mmol) in CH_2Cl_2 (6.0 mL) was added MnO_2 (1.13 g, 13.1 mmol). After stirring for 15 min, the reaction mixture was filtered through a short pad of Celite®. The filtrate was concentrated *in vacuo* to afford the corresponding aldehyde, which was used without further purification.

To a suspension of $(Ph_3PCH_2I)^+I^-$ (712 mg, 1.34 mmol) in THF (18 mL) was added NaHMDS (1M in THF, 1.34 mL, 1.34 mmol). The solution was stirred until a deep orange solution was obtained. The reaction mixture was then cooled to −78 °C, HMPA (383 µL) was added, followed by the crude aldehyde in THF (18 mL). The reaction mixture was allowed to warm to rt over 1

h, before being diluted with hexane, filtered through a short pad of Celite® and concentrated *in vacuo*. The residue was taken up in hexane, washed with water and brine, dried ($MgSO_4$), filtered through Celite® and concentrated *in vacuo* to afford vinyl iodide **4** (356 mg, 0.522 mmol, 99%, single geometrical isomer) as a yellow oil.

R$_f$ 0.35 (PE); $[\alpha]_D^{20}$ = +42.1 (*c* 0.42, $CHCl_3$); **IR** ν_{max} = 2955, 2925, 2874, 1600, 1457; **^{1}H NMR** (500 MHz, $CDCl_3$) δ_H 6.76 (1H, d, J = 8.4 Hz, H6), 6.49 (1H, dd, J = 12.9, 8.5 Hz, H12), 6.09 (1H, d, J = 8.4 Hz, H7), 5.86 (1H, d, J = 12.9 Hz, H13), 5.71 (1H, d, J = 9.8 Hz, H9), 3.82 (1H, dd, J = 8.5, 4.0 Hz, H11), 2.55 (1H, dqd, J = 9.8, 6.9, 3.9 Hz, H10), 1.88 (3H, s, Me8), 1.53 – 1.46 (6H, m, $Sn(CH_2CH_2\underline{CH_2}CH_3)_3$), 1.36 – 1.29 (6H, m, $Sn(CH_2\underline{CH_2}CH_2CH_3)_3$), 1.02 (3H, d, J = 6.9 Hz, Me10), 0.95 – 0.89 (24H, m, $Sn(\underline{CH_2}CH_2CH_2\underline{CH_3})_3$, $Si(CH_2\underline{CH_3})_3$), 0.57 (6H, q, J = 7.8 Hz, $Si(\underline{CH_2}CH_3)_3$); **^{13}C NMR** (125 MHz, $CDCl_3$) δ_C 152.0, 142.6, 136.8, 132.1, 128.0, 79.9, 74.2, 40.7, 29.2, 27.4, 17.0, 15.9, 13.8, 10.4, 7.1, 5.2; **HRMS** (ES$^+$) calc for $C_{28}H_{54}IOSi^{112}Sn$ $[M-H]^+$ 673.2031, found 673.2019.

c. North-eastern fragment 5

Vinyl stannane S7

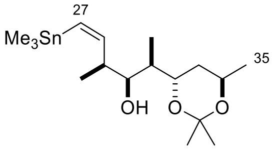

To a solution of vinyl iodide **14**[1] (230 mg, 625 µmol) in THF (10 mL) was added $PdCl_2(PPh_3)_2$ (21.9 mg, 31.3 µmol), Li_2CO_3 (231 mg, 3.12 mmol) and Me_6Sn_2 (1.02 mL, 3.12 mmol). The reaction mixture was stirred at 40 °C for 5 h before it was cooled to rt and concentrated *in vacuo*. Purification by flash chromatography on Florisil® (EtOAc/PE 0:1 → 1:20) provided stannane **S7** (165 mg, 407 µmol, 65%) as a colourless oil.

R$_f$ 0.50 (EtOAc/hexane 1:9); **$[\alpha]_D^{20}$** = +14.3 (*c* 1.03, $CHCl_3$); **IR** ν_{max} 3537, 2974, 2937, 1595, 1459, 1379; **^{1}H NMR** (500 MHz, $CDCl_3$) δ_H 6.36 (1H, dd, *J* = 12.4, 9.5 Hz, H28), 5.94 (1H, d, *J* = 12.3 Hz, H27), 3.99 – 3.92 (1H, m, H34), 3.89 (1H, dt, *J* = 9.8, 6.4 Hz, H32), 3.72 (1H, ddd, *J* = 9.2, 1.7, 1.7 Hz, H30), 2.31 (1H, d, *J* = 1.9 Hz, OH30), 2.19 – 2.08 (1H, m, H29), 1.78 (1H, ddd, *J* = 15.8, 9.8, 6.0 Hz, H33a), 1.69 – 1.61 (1H, m, H31), 1.55 (1H, ddd, *J* = 15.7, 9.5, 6.2 Hz, H33b), 1.34 (3H, s, $C\underline{Me_2}$), 1.34 (3H, s, $C\underline{Me_2}$), 1.20 (3H, d, *J* = 6.3 Hz, H35), 0.92 (3H, d, *J* = 6.7 Hz, Me29), 0.88 (3H, d, *J* = 7.0 Hz, Me31), 0.18 (9H, s, $SnMe_3$); **^{13}C NMR** (125 MHz, $CDCl_3$) δ_C 152.4, 130.7, 100.5, 72.4, 69.4, 63.1, 44.6, 38.6, 38.5, 24.9, 24.5, 21.7, 17.1, 8.8, –8.3; **HRMS** (ES+) calcd for $C_{17}H_{34}NaO_3{}^{120}Sn$ $[M+Na]^+$ 429.1428, found 429.1424.

Phosphonate 5

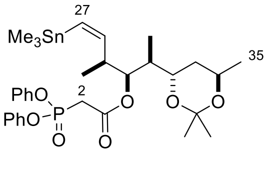

To a solution of alcohol **S7** (100 mg, 247 µmol) and acid **15**[5] (144 mg, 494 µmol) in PhMe (5.0 mL) was added DCC (102 mg, 494 µmol) and the mixture was stirred for 5 min before being concentrated *in vacuo*. Purification by flash chromatography (EtOAc/PE/Et_3N 1:10:0.02) afforded phosphonate **5** (130 mg, 191 µmol, 77%) as a yellow oil.

R_f 0.45 (EtOAc/PE 1:5); $[\alpha]_D^{20}$ = +5.8 (*c* 0.98, $CHCl_3$); **IR** ν_{max} = 2972, 2934, 1737, 1703, 1593; **^{1}H NMR** (500 MHz, $CDCl_3$) δ_H 7.35 – 7.29 (4H, m, ArH), 7.24 – 7.21 (4H, m, ArH), 7.21 – 7.16 (2H, m, ArH), 6.34 (1H, dd, J = 12.4, 9.9 Hz, H28), 5.75 (1H, d, J = 12.4 Hz, H27), 5.36 (1H, dd, J = 9.4, 1.8 Hz, H30), 3.88 (1H, ddq, J = 9.2, 6.1, 6.1 Hz, H34), 3.60 (1H, dt, J = 9.6, 6.1 Hz, H32), 3.24 – 3.11 (2H, m, H2), 2.33 (1H, ddq, J = 9.8, 9.4, 6.9 Hz, H29), 1.79 (1H, dqd, J = 9.5, 6.9, 1.8 Hz, H31), 1.58 (1H, ddd, J = 12.6, 9.6, 6.1 Hz, H33a), 1.41 (1H, ddd, J = 12.6, 9.2, 6.1 Hz, H33b), 1.32 (3H, s, CMe_2), 1.30 (3H, s, CMe_2), 1.10 (3H, d, J = 6.1 Hz, Me35), 1.00 (3H, d, J = 6.9 Hz, Me29), 0.90 (3H, d, J = 6.9 Hz, Me31), 0.18 (9H, s, $SnMe_3$); **^{13}C NMR** (125 MHz, $CDCl_3$) δ_C 163.9 (d, J = 6.5 Hz), 150.9, 149.9 (d, J = 2.6 Hz), 149.8 (d, J = 2.6 Hz), 129.8, 129.8, 129.7, 125.5, 120.8 (d, J = 3.2 Hz), 120.8, d (J = 3.7 Hz), 100.6, 77.2, 66.8, 62.5, 44.2, 39.6, 39.1, 34.7, 33.6, 24.7 (d, J = 20.8 Hz), 21.7, 17.7, 8.3, –8.4; **HRMS** (ES^+) calc for $C_{31}H_{45}O_7P^{112}Sn$ $[M+H]^+$ 681.2004, found 681.1992.

d. Revised North-eastern Fragment 20

Acetonide S8

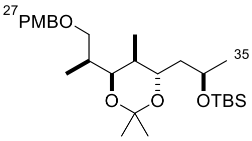

To a solution of diol **22**[1] (6.20 g, 14.1 mmol) in CH_2Cl_2 (200 mL) was added 2,2-dimethoxypropane (85.4 mL, 697 mmol) and pyridinium *p*-toluenesulfonate (360 mg, 1.43 mmol). After stirring for 16 h, the reaction mixture was quenched with $NaHCO_3$ solution (100 mL), extracted with CH_2Cl_2 (2 x 200 mL) and the combined organic extracts were dried ($MgSO_4$) and concentrated *in vacuo*. Purification by flash chromatography (EtOAc/PE 1:9) afforded acetonide **S8** (6.29 g, 13.1 mmol, 93%) as a yellow oil.

R_f 0.81 (EtOAc/hexane 3:7); **[α]** $_D^{20}$ = –13.8 (*c* 0.80, $CHCl_3$); **IR** (thin film) ν_{max} = 2932, 1614, 1514; **^{1}H NMR** (500 MHz, $CDCl_3$) δ_H 7.24 (2H, d, J = 8.6 Hz, ArH), 6.87 (2H, d, J = 8.6 Hz, ArH), 4.40 (2H, s, OCH_2Ar), 3.95 – 3.88 (1H, m, H34), 3.80 (3H, s, $ArOCH_3$), 3.64 (1H, dd, J = 10.8, 4.6 Hz, H30), 3.54 (1H, dd, J = 9.0, 4.2 Hz, H28a), 3.41 – 3.38 (1H, m, H32), 3.38 (1H, dd, J = 9.0, 6.2 Hz, H28b), 1.88 – 1.79 (1H, m, H29), 1.59 – 1.56 (1H, m, H31), 1.56-1.52 (2H, m, H33a, H33b), 1.33 (3H, s, CMe_2), 1.27 (3H, s, CMe_2), 1.14 (3H, d, J = 5.9 Hz, H35), 0.93 (3H, d, J = 6.7 Hz, Me29), 0.88 (9H, s, Si*t*BuMe$_2$), 0.84 (3H, d, J = 7.0 Hz, Me30), 0.06 (3H, s, Si*t*BuMe$_2$), 0.04 (3H, s, Si*t*BuMe$_2$); **^{13}C NMR** (125 MHz, $CDCl_3$) δ_c 159.2, 131.1, 129.1, 113.7, 100.6, 72.8, 72.3, 72.3, 69.9, 65.7, 55.3, 46.2, 38.5, 33.8, 29.7, 25.9, 24.9, 24.0, 18.0, 13.5, 11.1, –3.5, –4.7; **HRMS** (ES+) calc for $C_{27}H_{49}O_5Si$ $[M+H]^+$ 481.3349, found 481.3352.

Alcohol 23

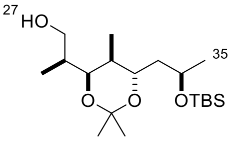

To a solution of PMB ether **S8** (6.20 g, 12.9 mmol) in CH_2Cl_2 (200 mL) and pH 7 buffer solution (40 mL) at 0 °C was added DDQ (3.22 g, 14.2 mmol) in three portions. After stirring for 1 h at 0 °C, the reaction mixture was quenched with pH 7 buffer solution (250 mL, aq.) and the phases separated. The aqueous layer was extracted with CH_2Cl_2 (3 x 100 mL) and the combined organic layers dried ($MgSO_4$) and concentrated *in vacuo*. Purification by flash chromatography (EtOAc/PE 1:9) yielded alcohol **23** (4.20 g, 11.7 mmol, 91%) as a colourless oil.

R$_f$ 0.30 (EtOAc/hexane 1:4); **[α]**$^{20}_{D}$ = −22.9 (*c* 0.96, PhH) ; **IR** ν_{max} = 3496, 2958, 2933, 2858, 1462, 1378; **^{1}H NMR** (500 MHz, $CDCl_3$) δ_H 3.96 – 3.87 (1H, m, H34), 3.67 (1H, dd, J = 10.5, 4.5 Hz, H30), 3.57 (1H, ddd, J = 10.7, 8.1, 1.6 Hz, H32), 3.55 – 3.48 (1H, m, J, H28a), 3.46 – 3.40 (1H, m, H28b), 3.19 (1H, dd, J = 9.6, 1.7 Hz, OH), 1.96 – 1.85 (1H, m, H29), 1.62 – 1.57 (1H, m, H31), 1.57 – 1.52 (2H, m, H33), 1.38 (3H, s, CMe_2), 1.34 (3H, s, CMe_2), 1.14 (3H, d, J = 6.2 Hz, H35), 0.89 (9H, s, Si<u>tBu</u>Me_2), 0.87 (3H, d, J = 6.9 Hz, Me29), 0.76 (3H, d, J = 6.8 Hz, Me31), 0.06 (3H, s, Si*t*Bu<u>Me$_2$</u>), 0.04 (3H, s, Si*t*Bu<u>Me$_2$</u>); **^{13}C NMR** (125 MHz, $CDCl_3$) δ_C 100.7, 75.7, 72.0, 69.1, 65.6, 46.1, 38.8, 35.0, 25.9, 25.1, 24.7, 24.3, 18.0, 12.7, 11.1, −3.5, −4.7; **HRMS** (ES+) calc for $C_{19}H_{40}NaO_4Si$ $[M+Na]^+$ 383.2594, found 383.2583.

Vinyl iodide S9

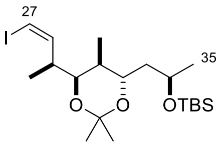

To a stirred solution of alcohol **23** (3.92 g, 10.9 mmol) in DCM (70 mL) was added $NaHCO_3$ (5.4 g, 64 mmol) and Dess-Martin periodinane (13.6 g, 32.2 mmol). The reaction mixture was stirred for 30 min, before being quenched with $NaHCO_3$, extracted with CH_2Cl_2, and the organic extracts were dried ($MgSO_4$) and concentrated *in vacuo*. Purification by flash chromatography on silica gel (EtOAc/PE 1:9) gave the ensuing aldehyde (3.36 g, 9.37 mmol, 86%) as a yellow oil, which was immediately used in the subsequent step.

To a suspension of $(Ph_3PCH_2I)^+I^-$ (12.2 g, 23.0 mmol) in THF (150 mL) was added NaHMDS (1M in THF, 23.0 mL, 23.0 mmol). The solution was stirred until a deep orange solution was obtained. The reaction mixture was then cooled to −78 °C, and the freshly prepared aldehyde (3.33 g, 9.37 mmol) in THF (100 mL) was added. The reaction mixture was warmed to rt over 2 h, before being diluted with hexane, filtered through a short pad of Celite® and concentrated *in vacuo*. Purification by flash chromatography (EtOAc/PE 1:20) afforded vinyl iodide **S9** (4.20 g, 8.71 mmol, 93%, *Z*-isomer only) as a yellow oil.

R$_f$ 0.89 (EtOAc/hexane 1:4); **[α]**$^{20}_{D}$ = +18.2 (*c* 0.88, $CHCl_3$); **IR** ν_{max} = 2960, 2932, 2353, 1461, 1378, 1256; **^{1}H NMR** (500 MHz, $CDCl_3$) δ_H 6.14 (1H, d, J = 7.5 Hz, H27), 6.12 (1H, t, J = 7.4 Hz, H28), 3.96 – 3.88 (1H, m, H34), 3.70 (1H, dd, J = 9.1, 4.7 Hz, H30), 3.43 – 3.36 (1H, m H32), 2.64 – 2.54 (1H, m, H29), 1.65 – 1.57 (1H, m, H31), 1.57 – 1.52 (2H, m, H33), 1.31 (3H, s, CMe_2), 1.29 (3H, s, CMe_2), 1.45 (3H, d, J = 6.2 Hz, H35), 1.14 (3H, d, J = 6.1 Hz, Me29), 0.89 (9H, s, Si<u>tBu</u>Me_2), 0.87 (3H, d, J = 6.3 Hz, Me31), 0.06 (3H, s, Si*t*Bu<u>Me$_2$</u>), 0.04 (3H, s, Si*t*Bu<u>Me$_2$</u>); **^{13}C NMR** (125 MHz, $CDCl_3$) δ_C 144.5, 100.6, 81.3, 72.1, 71.9, 65.6, 46.0, 39.5, 39.0, 25.9, 24.8, 24.7, 24.2, 18.0, 15.6, 11.3, −3.5, −4.7; **HRMS** (ES+) calculated for $C_{20}H_{39}NaIO_3Si$ $[M+Na]^+$ 505.1611, found 505.1613.

Triol 24

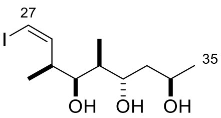

To a solution of TBS ether **S9** (3.55 g, 7.36 mmol) in MeOH (50 mL) was added PPTS (555 mg, 2.21 mmol). The mixture was stirred for 16 h, the volatiles were removed *in vacuo* and the crude product purified by flash column chromatography (EtOAc / PE 1:4) to yield triol **24** (2.17 g, 6.62 mmol, 90%) as an off-white solid.

Rf 0.29 (EtOAc/PE 1:4); $[\alpha]_D^{20}$ = +30.0 (c 0.20, $CHCl_3$); **IR** ν_{max} = 3347, 2966, 2930, 2348, 2326, 1456, 1376, 1260, 1066, 972, 804, 699; ^{1}**H NMR** (500 MHz, $CDCl_3$) δ_H 6.33 (1H, d, J = 7.4 Hz, H27), 6.18 (1H, dd, J = 8.8, 7.4 Hz, H28), 4.24-4.15 (1H, m, H34), 4.05 – 3.97 (1H, m, H32), 3.92 – 3.87 (1H, m, H30), 3.12 (3H, d, J = 5.2 Hz, OH), 2.78 – 2.69 (1H, m, H29), 1.82 (1H, ddd, J = 14.5, 9.6, 3.1 Hz, H33a), 1.77 – 1.70 (1H, m, H31), 1.56 (1H, ddd, J = 14.5, 7.4, 2.4 Hz, H33b), 1.28 (3H, d, J = 6.3 Hz, H35), 1.03 (3H, d, J = 7.1 Hz, Me31), 0.96 (3H, d, J = 6.8 Hz, Me29); ^{13}**C NMR** (125 MHz, $CDCl_3$) δ_C 144.0, 83.3, 75.0, 72.6, 65.9, 43.1, 42.2, 39.3, 23.4, 16.0, 10.7; **HRMS** (ES^+) calc for $C_{11}H_{22}IO_3$ $[M+H]^+$ 329.0608, found 329.0611.

Alcohol 25

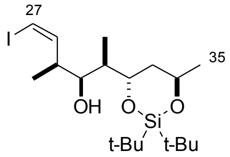

To a solution of triol **24** (500 mg, 1.52 mmol) in CH_2Cl_2 (20 mL) at –78 °C was added 2,6-lutidine (0.88 mL, 7.60 mmol) and Di-*t*-butylsilyl bis(trifluoromethanesulfonate) (0.59 mL, 1.82 mmol) dropwise. The reaction mixture was stirred at –78 °C for 1h, before being quenched with MeOH (5 mL) followed by $NaHCO_3$ solution (20 mL). The residue was extracted with CH_2Cl_2 (3 × 20 mL), and the combined organic extracts were dried ($MgSO_4$) and concentrated in *vacuo*. Purification by flash chromatography (EtOAc/PE 1:20) afforded alcohol **25** (684 mg, 1.46 mmol, 96%) as a colourless oil.

Rf 0.25 (Et_2O/PE 9:1); $[\alpha]_D^{20}$ = +59.5 (c 1.0, $CHCl_3$); **IR** ν_{max} = 3481, 2964, 2932, 2892, 2858, 1474, 1385, 1259, 1134, 978, 896, 865, 825, 797, 730, 648; ^{1}**H NMR** (500 MHz, $CDCl_3$) δ_H 6.29 – 6.23 (2H, m, H27, H28), 4.47 – 4.40 (1H, m, H34), 4.24 (1H, ddd, J = 10.1, 5.4, 1.9 Hz, H32), 3.96 (1H, dd, J = 8.0, 1.7 Hz, H30), 3.00 (1H, brs, OH), 2.74 – 2.64 (1H, m, H29), 2.19 (1H, ddd, J = 14.3, 10.1, 5.9 Hz, H33a), 1.72 – 1.65 (1H, m, H31), 1.52 – 1.47 (1H, m, H33b), 1.30 (3H, d, J = 6.6 Hz, H35), 1.03 (3H, d, J = 7.0 Hz, Me31), 1.00 (18H, s, Si*t*Bu$_2$), 0.95 (3H, d, J = 6.9 Hz, Me29); ^{13}**C NMR** (125 MHz, $CDCl_3$) δ_C 144.8, 82.2, 74.6, 73.2, 67.8, 43.0, 40.7, 38.8, 27.5, 27.4, 23.8, 21.5, 20.8, 16.5, 10.9; **HRMS** (ES^+) calc for $C_{19}H_{38}IO_3Si$ $[M+H]^+$ 469.1629, found 469.1619.

Vinyl stannane S10

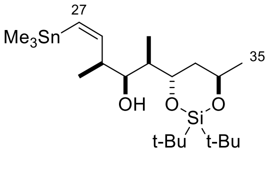

To a stirred solution of vinyl iodide **25** (916 mg, 1.96 mmol) in THF (20 mL) was added $PdCl_2(PPh_3)_2$ (138 mg, 0.20 mmol), Li_2CO_3 (723 mg, 9.78 mmol) and $(Me_3Sn)_2$ (2.03 mL, 9.78 mmol). The reaction mixture was stirred at 40 °C for 3h, cooled to rt and concentrated *in vacuo*. Purification by flash chromatography (EtOAc/PE 1:20) afforded stannane **S10** (644 mg, 1.27 mmol, 65%) as a colourless oil.

Rf 0.58 (Et_2O/PE 1:9); $[\alpha]_D^{20}$ = +65.0 (c 0.1, $CHCl_3$); **IR** ν_{max} = 2962, 2930, 2857, 2360, 1726, 1598, 1474, 1376, 1258, 1133, 1101, 981, 899, 864, 825, 799, 769, 730; ^{1}**H NMR** (500 MHz, $CDCl_3$) δ_H 6.38 (1H, dd, J = 12.3, 9.5 Hz, H28), 5.94 (1H, d, J = 12.4 Hz, H28), 4.39 (1H, dqd , J = 6.1, 2.6 Hz, H34), 4.24 (1H, ddd, *J* = 9.4, 6.4, 2.4 Hz, H32), 3.96 (1H, brd, J = 8.0 Hz, H30),

2.51 (1H, d, J = 1.8 Hz, OH), 2.15 (1H, dqd, J = 9.5, 6.5, 3.1 Hz, H29), 2.11 (1H, ddd, *J* = 14.3, 9.8, 5.7 Hz, H33a), 1.64 (1H, dqd, J = 7.1, 7.1, 1.5 Hz, H31), 1.56 (1H, ddd, J = 14.2, 2.4, 2.4 Hz, H33b), 1.29 (3H, d, J = 6.7 Hz, H35), 1.01 (9H, s, Si*t*Bu$_2$), 0.99 (9H, s, Si*t*Bu$_2$), 0.92 (3H, d, J = 7.1 Hz, Me31), 0.91 (3H, d, *J* = 6.7 Hz, Me29) 0.18 (9H, s, Sn$\underline{Me_3}$); **^{13}C NMR** (125 MHz, $CDCl_3$) δ_C 152.5, 130.4, 73.0, 72.4, 67.6, 44.6, 39.9, 38.7, 27.3, 27.3, 23.7, 21.4, 20.7, 17.4, 9.7, -8.3; **HRMS** (ES$^+$) calc for $C_{22}H_{47}O_3Si^{112}Sn$ $[M+H]^+$ 499.2337, found 499.2337.

Phosphonate 20

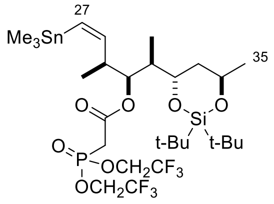

To a solution of alcohol **S10** (410 mg, 811 µmol) and acid **26** (370 mg, 1.22 mmol) in CH_2Cl_2 (20 mL) was added DCC (1 M, 0.89 mL, 890 µmol) and the reaction mixture was stirred for 10 min before being concentrated *in vacuo*. Purification by flash chromatography (EtOAc/PE 1:20) afforded phosphonate **20** (488 mg, 616 µmol, 76%) as a yellow oil.

Rf 0.21 (Et_2O/PE 1:9); $[\alpha]_D^{20}$ = +62.4 (c 1.0, $CHCl_3$); **IR** ν_{max} = 2971, 2860, 1736, 1474, 1386, 1298, 1267, 1174, 1143, 1097, 1071, 963, 900, 826, 769, 648; **^{1}H NMR** (500 MHz, $CDCl_3$) δ_H 6.30 (1H, dd, *J* = 12.3, 9.8 Hz, H28), 5.81 (1H, d, *J* = 12.2 Hz, H27), 5.54 (1H, dd, *J* = 9.6, 1.3 Hz, H30), 4.51 – 4.38 (4H, m, O$C\underline{H_2}CF_3$), 4.39 – 4.32 (1H, m, H34), 3.82 (1H, ddd, *J* = 9.8, 8.9, 3.1 Hz, H32), 3.13 – 2.96 (2H, m, H2), 2.36 – 2.26 (1H, m, H29), 1.87 – 1.76 (2H, m, H31, H33a), 1.67 (1H, ddd, *J* = 14.4, 4.2, 3.6 Hz, H33b), 1.28 (3H, d, *J* = 6.4 Hz, Me35), 1.00 (9H, s, Si$\underline{tBu_a}tBu_b$), 0.99 (3H, d, *J* = 6.5 Hz, Me29), 0.98 (9H, s, Si$tBu_a\underline{tBu_b}$), 0.84 (3H, d, *J* = 7.0 Hz, Me31), 0.18 (9H, s, Sn$\underline{Me_3}$); **^{13}C NMR** (125 MHz, $CDCl_3$) δ_C 164.1 (d, J = 2.8 Hz), 151.0, 129.9, 128.5, 122.3 (qd, J = 278, 6.5 Hz), 122.2 (qd, J = 278, 6.0 Hz), 76.9, 69.9, 67.3, 63.0 – 62.2 (2C, m), 44.3, 40.9, 38.2, 34.1, 27.5, 27.2, 23.8, 20.6 (d 33.5 Hz), 17.7, 9.5, −8.3; **HRMS** (ES$^+$) calc for $C_{28}H_{55}F_6O_7PSi^{112}SnN$ $[M+NH_4]^+$ 802.2434, found 802.2439.

e. Fragment coupling strategy 1 – Approach I

Trienoate 16

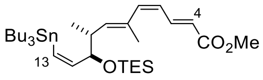

To Pd_2dba_3 (23.5 mg, 25.7 µmol) and tBu_3P (20.8 mg, 103 µmol) was added DMF (2 mL) and the catalyst solution was stirred for 5 min before it was cooled to 0 °C and placed in the dark. A solution of stannane **6** (241 mg, 642 µmol) in DMF (2 mL) was added, followed by a solution of CuTC (73.7 mg, 386 µmol) and $Ph_2PO_2NBu_4$ (593 mg, 1.29 mmol) in DMF (2 mL) and a solution of iodide **4** (175 mg, 257 µmol) in DMF (10 mL). After stirring for 1 h, complete consumption of iodide **4** was observed by TLC. An additional portion of CuTC (3.1 mg, 16.2 µmol) was added and the reaction mixture was stirred for 15 min, before being diluted with PE and quenched with $NaHCO_3$ solution (10 mL). The aqueous phase was extracted with Et_2O/PE (1:1) (2 × 5 mL) and the combined organic extracts were dried ($MgSO_4$) and concentrated *in vacuo*. Purification by flash chromatography on Florisil® (Et_2O/PE 1:10) gave triene **16** (118 mg, 185 µmol, 72%) as a colourless oil.

R$_f$ 0.60 (Et_2O/PE 1:10); $[\alpha]_D^{20}$ = +39.1 (*c* 1.90, $CHCl_3$); **IR** ν_{max} = 2955, 2922, 2874, 1721, 1620; **^{1}H NMR** (500 MHz, $CDCl_3$) δ_H 7.85 (1H, dd, J = 15.2, 12.0 Hz, H5), 6.46 (1H, dd, J = 13.0, 8.4 Hz, H12), 6.23 (1H, d, J = 11.5 Hz, H7), 6.01 (1H, dd, J = 12.2, 11.5 Hz, H6), 5.88 (1H, d, J = 13.0 Hz, H13), 5.85 (1H, d, J = 15.1 Hz, H4), 5.63 (1H, d, J = 9.7 Hz, H9), 3.82 (1H, dd, J = 8.5, 4.0 Hz, H11), 3.74 (3H, s, CO_2Me), 2.50 (1H, dqd, J = 9.7, 6.9, 4.0 Hz, H10), 1.88 (3H, s, Me8), 1.53 – 1.46 (6H, m, $Sn(CH_2CH_2\underline{CH_2}CH_3)_3$), 1.36 – 1.28 (6H, m, $Sn(CH_2\underline{CH_2}CH_2CH_3)_3$), 1.04 (3H, d, J = 6.9 Hz, Me10), 0.95 – 0.86 (24H, m, $Sn(\underline{CH_2}CH_2CH_2\underline{CH_3})_3$, $Si(CH_2\underline{CH_3})_3$), 0.56 (6H, q, J = 7.8 Hz, $Si(\underline{CH_2}CH_3)_3$); **^{13}C NMR** (125 MHz, $CDCl_3$) δ_C 167.7, 151.7, 142.6, 141.6, 139.2, 133.0, 128.0, 124.4, 121.0, 79.8, 51.4, 41.0, 29.2, 27.4, 17.2, 16.8, 13.6, 10.4, 6.8, 5.2; **HRMS** (ES^+) calc for $C_{32}H_{60}O_3Si^{112}Sn$ $[M+H]^+$ 633.3433, found 633.3440.

Heptaene 17

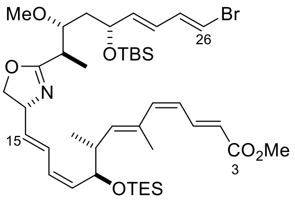

To Pd_2dba_3 (2.2 mg, 2.4 µmol) and *t*Bu_3P (2.0 mg, 9.4 µmol) was added DMF (0.4 mL) and the catalyst solution was stirred for 5 min before it was cooled to 0 °C and kept in the dark. A solution of stannane **16** (15.0 mg, 23.5 µmol) and *bis*-halide **3** (20.6 mg, 35.3 µmol) in DMF (0.8 mL) was added, followed by a solution of CuTC (9.0 mg, 47.0 µmol) and $Ph_2PO_2NBu_4$ (52.4 mg, 118 µmol) in DMF (0.8 mL). After stirring for 2 h, the reaction mixture was diluted with PE and quenched with $NaHCO_3$ solution (5 mL). The aqueous phase was extracted with Et_2O/PE (1:1) (2 × 5 mL) and the combined organic extracts were dried ($MgSO_4$) and concentrated *in vacuo*. Purification by flash chromatography on Florisil® (EtOAc/PE 1:7 → 1:4) and preparative TLC (EtOAc/PE 5:1) gave heptaene **17** (13.5 mg, 16.7 µmol, 71%) as a colourless oil.

R$_f$ 0.72 (EtOAc/PE 1:3); **^{1}H NMR** (500 MHz, $CDCl_3$) δ_H 7.85 (1H, dd, J = 15.3, 12.0 Hz, H5), 6.67 (1H, dd, J = 13.4, 10.8 Hz, H25), 6.41 (1H, dd, J = 15.3, 11.5 Hz, H14), 6.26 (1H, d, J = 13.6 Hz, H26), 6.22 (1H, d, J = 11.5 Hz, H7), 6.07 (1H, dd, J = 15.3, 10.9 Hz, H24), 6.01 (1H, dd, J = 11.6, 11.6 Hz, H6), 5.97 (1H, dd, J = 11.1, 11.1 Hz, H13), 5.85 (1H, d, J = 15.1 Hz, H4), 5.69 (1H, dd, J = 15.2, 6.7 Hz, H23), 5.57 (1H, dd, J = 15.2, 8.5 Hz, H15), 5.53 (1H, d, J = 9.4 Hz, H9), 5.42 (1H, dd, J = 11.1, 8.8 Hz, H12), 4.62 (1H, ddd, J = 8.6, 8.6, 8.6 Hz, H16), 4.42 – 4.36 (2H, m, H11, H17a), 4.31 – 4.27 (1H, m, H22), 3.87 (1H, t, J = 8.6 Hz, H17b), 3.74 (3H, s, CO_2Me), 3.72 – 3.69 (1H, m, H20), 3.37 (3H, s, OMe20), 2.96 – 2.92 (1H, m, H19), 2.61 – 2.54 (1H, m, H10), 1.89 (3H, s, Me8), 1.54 – 1.48 (2H, m, H21a, H21b), 1.13 (3H, d, J = 7.2 Hz, Me19), 0.99 (3H, d, J = 6.9 Hz, Me10), 0.93 – 0.89 (18H, m, Si*t*<u>Bu</u>Me$_2$, $Si(CH_2\underline{CH_3})_3$), 0.54 (6H, q, J = 7.8 Hz, $Si(\underline{CH_2}CH_3)_3$), 0.06 (3H, s, Si*t*Bu<u>Me$_2$</u>), 0.00 (3H, s, Si*t*Bu<u>Me$_2$</u>).

Octaene 18

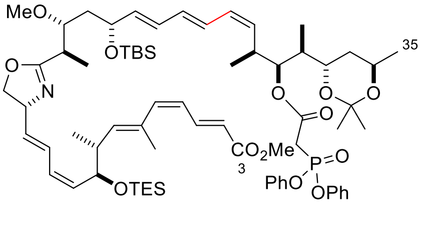

To Pd_2dba_3 (0.9 mg, 0.99 µmol) and *t*Bu_3P (0.8 mg, 4.0 µmol) was added DMF (0.1 mL) and the catalyst solution was stirred for 5 min before it was cooled to 0 °C. A solution of bromide **17** (8.0 mg, 9.9 µmol) and stannane **5** (10.1 mg, 14.9 µmol) in DMF (0.2 mL) was added, followed by a solution of CuTC (3.8 mg, 19.8 µmol) and $Ph_2PO_2NBu_4$ (22 mg, 49.5 µmol) in DMF (0.3 mL). After stirring for 30 min in the dark, the reaction mixture was diluted with PE and quenched with $NaHCO_3$ solution

(2 mL). The aqueous phase was extracted with Et_2O/PE (1:1) and the combined organic extracts were dried ($MgSO_4$) and concentrated *in vacuo*. Purification by flash chromatography on Florisil® (EtOAc/PE 1:3 → 1:2) gave octaene **18** (10.1 mg, 8.2 µmol, 83%) as a pale yellow oil.

R_f 0.26 (EtOAc/PE 1:3); $[\alpha]_D^{20}$ = +5.9 (*c* 1.30, $CHCl_3$); **IR** ν_{max} = 2956, 2878, 2160, 1738, 1655, 1613, 1593; **^{1}H NMR** (500 MHz, $CDCl_3$) δ_H 7.84 (1H, dd, J = 15.2, 12.3 Hz, H5), 7.33 – 7.29 (4H, m, Ph), 7.22 – 7.15 (6H, m, Ph), 6.41 (1H, dd, J = 15.2, 11.2 Hz, H14), 6.40 (1H, dd, J = 14.3, 11.1 Hz, H26), 6.21 (1H, d, J = 11.5 Hz, H7), 6.14 (1H, dd, J = 11.1, 10.5 Hz, H27), 6.12 (1H, dd, J = 14.4, 10.9 Hz, H24), 6.01 (1H, dd, J = 11.5, 11.5 Hz, H6), 5.97 (1H, dd, J = 11.2, 11.2 Hz, H13), 5.97 (1H, dd, J = 14.3, 10.9 Hz, H25), 5.85 (1H, d, J = 15.5 Hz, H4), 5.65 (1H, dd, J = 14.4, 7.1 Hz, H23), 5.58 (1H, dd, J = 15.2, 8.1 Hz, H15), 5.52 (1H, d, J = 9.7 Hz, H9), 5.41 (1H, dd, J = 11.2, 9.4 Hz, H12), 5.34 (1H, dd, J = 10.7, 10.7 Hz, H28), 5.27 (1H, dd, J = 8.2, 2.4 Hz, H30), 4.62 (1H, ddd, J = 8.8, 8.8, 8.8 Hz, H16), 4.39 (1H, dd, J = 9.2, 5.3 Hz, H11), 4.40 – 4.31 (2H, m, H22, H17a) 3.91 – 3.85, m, H34, H17b), 3.74 (3H, s, CO_2Me), 3.73 – 3.69 (1H, m, H20), 3.62 (1H, ddd, J = 9.4, 5.6, 5.6 Hz, H32), 3.37 (3H, s, OMe20), 3.23 – 3.09 (2H, m, H2), 3.01 – 2.96 (1H, m, H29), 2.96 – 2.91 (1H, m, H19), 2.60 – 2.54 (1H, m, H10), 1.89 (3H, s, Me8), 1.86 – 1.80 (1H, m, H31), 1.64 – 1.58 (1H, m, H33a), 1.56 – 1.50 (2H, m, H21a, H21b), 1.45 – 1.40 (1H, m, H33b), 1.32 (3H, s, C$\underline{Me_2}$), 1.29 (3H, s, C$\underline{Me_2}$), 1.13 (3H, d, J = 7.2 Hz, Me19), 1.12 (3H, d, J = 6.1 Hz, H35), 1.00 (3H, d, J = 6.9 Hz, Me10), 0.98 (3H, d, J = 7.1 Hz, Me29), 0.92 – 0.89 (21H, m, Me31, Si$\underline{t\text{Bu}}$$Me_2$ and Si($\underline{CH_2}$$CH_3$)$_3$), 0.53 (6H, q, J = 8.3 Hz, Si($CH_2$$\underline{CH_3}$)$_3$), 0.06 (3H, s, Si*t*Bu$\underline{Me_2}$), 0.01 (3H, s, Si*t*Bu$\underline{Me_2}$); **^{13}C NMR** (125 MHz, $CDCl_3$) δ_C 169.7, 167.7, 164.1 (d, J = 6.4), 150.0 (d, J = 3.6 Hz), 149.9 (d, J = 3.6 Hz), 142.5, 141.6, 139.5, 138.1, 134.6, 134.5, 134.1, 133.6, 133.1, 129.8, 129.7, 129.3, 129.1, 127.7, 127.4, 127.0, 125.5, 125.4, 124.4, 121.0, 120.8, 120.7, 100.5, 78.2, 77.6, 72.3, 72.1, 70.2, 68.0, 66.9, 62.6, 57.1, 51.4, 40.3, 40.0, 39.8, 38.8, 35.2, 35.1, 29.7, 25.9, 24.7 (d, J = 30.0 Hz), 21.6, 18.1, 17.6, 16.9, 16.7, 10.8, 8.6, 6.8, 5.0, −3.7, −4.9; **HRMS** (ES^+) calc for $C_{69}H_{104}O_{13}NPSi_2$ $[M+H]^+$ 1242.6857, found 1242.6858.

One-Pot procedure

To Pd_2dba_3 (2.7 mg, 3.0 µmol) and *t*Bu_3P (2.4 mg, 11.8 µmol) was added DMF (0.5 mL) and the catalyst solution was stirred for 5 min before it was cooled to 0 °C and kept in the dark. A solution of iodide **4** (20 mg, 29.4 µmol) in DMF (0.5 mL) was added, followed by a solution of stannane **6** (14.3 mg, 38.2 µmol), CuTC (7.3 mg, 38.2 µmol) and $Ph_2PO_2NBu_4$ (167 mg, 147 mmol) in DMF (1.0 mL). After stirring for 30 min, complete consumption of iodide **4** was observed by TLC and a solution of *bis*-halide **3** (17.2 mg, 29.4 µmol) in DMF (1.0 mL) was added, followed by a solution of CuTC (7.3 mg, 38.2 µmol) in DMF (1.0 mL). The reaction mixture was stirred until complete consumption of *bis*-halide **3** was observed by TLC (30 min), after which a solution of stannane **5** (20.0 mg, 29.4 µmol) and CuTC (7.3 mg, 38.2 µmol) in DMF (1.0 mL) was added. The reaction mixture was warmed to rt and stirred for a further 30 min, before it was diluted with PE and quenched with $NaHCO_3$ solution (5 mL). The aqueous phase was extracted with Et_2O/PE (1:1) and the combined organic extracts were dried ($MgSO_4$) and concentrated *in vacuo*. Purification by flash chromatography on Florisil® (EtOAc/PE 1:3 → 1:2) gave octaene **18** (20.5 mg, 16.5 µmol, 56%) as a pale yellow oil, as characterised above.

f. Fragment coupling strategy 2 – Approach II

Pentaene 27

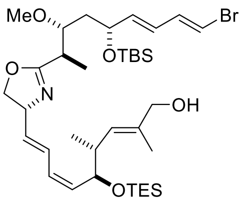

To a solution of $Pd(PPh_3)_4$ (2.5 mg, 2.2 µmol), CuTC (3.5 mg, 11.0 µmol) and $[Ph_2PO_2][NBu_4]$ (20.0 mg, 11.0 µmol) in DMF (0.4 mL) at 0 °C, was added a solution of *bis*-halide **3** (6.0 mg, 10.2 µmol) and stannane **19** (5.0 mg, 8.94 µmol) in DMF (0.4 mL). After stirring at 0 °C in the dark for 2 h, the reaction mixture was quenched with water (1 mL) and extracted with Et_2O (2 × 1 mL) and EtOAc (2 × 1 mL). The organic extracts were dried (Na_2SO_4) and concentrated *in vacuo*. Purification by flash chromatography (EtOAc/PE 1:4) gave pentaene **27** (5.4 mg, 7.43 µmol, 83%) as a pale yellow oil.

R$_f$ 0.25 (EtOAc/PE 1:4); **^{1}H NMR** (500 MHz, $CDCl_3$) δ_H 6.66 (1H, dd, *J* = 13.4, 10.9 Hz, H25), 6.37 (1H, dd, *J* = 15.0, 11.4 Hz, H14), 6.26 (1H, d, *J* = 13.4 Hz, H26), 6.06 (1H, dd, *J* = 15.3, 10.9 Hz, H24), 5.97 (1H, t, *J* = 11.2 Hz, H13), 5.69 (1H, dd, *J* = 15.3, 6.9 Hz, H23), 5.63 (1H, dd, *J* = 15.0, 5.7 Hz, H15), 5.39 (1H, dd, *J* = 11.2, 8.6 Hz, H12), 5.07 (1H, d, *J* =9.8 Hz, H9), 4.64 – 4.70 (1H, m, H16), 4.45 (1H, dd, J = 8.4, 4.2 Hz, H11), 4.33 (1H, dd, *J* = 9.8, 8.4 Hz, H17a), 4.26 – 4.30 (1H, m, H22), 3.94 – 3.97 (2H, m, H7), 3.92 (1H, t, *J* = 8.4 Hz, H17b), 3.69 (1H, ddd, *J* = 8.8, 5.1, 2.6 Hz, H20), 3.35 (3H, s, OMe), 2.90 – 2.97 (1H, m, H19), 2.58 – 2.66 (1H, m, H10), 1.72 (3H, d, *J* = 1.1 Hz, Me8), 1.45 – 1.56 (2H, m, H21), 1.15 (3H, d, *J* = 7.1 Hz, Me19), 0.94 (9H, t, *J* = 8.0 Hz, $Si(CH_2\underline{CH_3})_3$), 0.93 (3H, d, *J* = 6.7 Hz, Me10), 0.89 (9H, s, Si*t*BuMe$_2$), 0.56 (6H, q, *J* = 8.0 Hz, $Si(\underline{CH_2}CH_3)_3$), 0.06 (3H, s, Si*t*BuMe$_2$), 0.00 (3H, s, Si*t*BuMe$_2$); **^{13}C NMR** (125 MHz, $CDCl_3$) δ 170.4, 138.5, 136.9 135.7, 133.8, 132.8, 128.9, 127.2, 126.9, 126.5. 108.3, 78.0, 72.7, 71.7, 69.6, 69.1, 66.0, 57.0, 40.0, 39.8, 35.5, 25.9, 18.1, 14.3, 13.9, 11.1, 6.8, 4.9, −3.8, −4.9; **HRMS** (ES$^+$) calcd for $C_{36}H_{64}{}^{79}BrNO_5Si_2[M+H]^+$ 726.3585, found 726.3579.

Hexaene 28

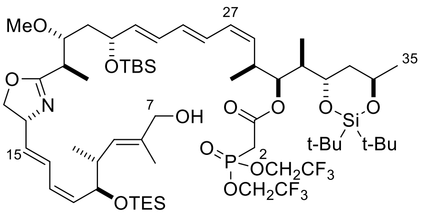

To a solution of $Pd(PPh_3)_4$ (10.5 mg, 9.05 µmol), CuTC (17.2 mg, 90.5 µmol) and $[Ph_2PO_2][NBu_4]$ (100 mg, 218 µmol) in DMF (3.0 mL) at 0 °C was added a solution of vinyl bromide **27** (31.0 mg, 42.5 µmol) and stannane **20** (71.6 mg, 90.5 µmol) in DMF (3.0 mL). After stirring for 2 h in the dark, the reaction mixture was quenched with water (5 mL) and extracted with Et_2O (2 × 5 mL) and EtOAc (2 × 5 mL). The extracts were dried (Na_2SO_4) and concentrated *in vacuo*. Purification by flash chromatography (EtOAc/PE 1:4) gave hexaene **28** (47.5 mg, 37.3 µmol, 88%) as a yellow oil.

One-Pot Procedure

To a solution of $Pd(PPh_3)_4$ (9.9 mg, 8.57 µmol), CuTC (13.7 mg, 71.8 µmol) and $[Ph_2PO_2][NBu_4]$ (33.0 mg, 71.8 µmol) in DMF (1.0 mL) at 0 °C as added a solution of *bis*-halide **3** (20.0 mg, 34.2 µmol) and stannane **19** (19.1 mg, 34.2 µmol) in DMF (1.0 mL). After stirring at 0 °C for 2 h in the dark, a solution of stannane **20** (43.4 mg, 54.8 µmol) in DMF (1.0 mL) was added. The reaction mixture was stirred for another 2 h before being quenched with water (3 mL) and extracted with Et_2O (2 × 2 mL) and EtOAc (2 × 2 mL). The extracts were dried (Na_2SO_4) and concentrated *in vacuo*. Purification by flash chromatography (EtOAc/PE 1:2) gave hexaene **28** (35.0 mg, 27.5 µmol, 80%) as a yellow oil.

Rf 0.4 (PE / EtOAc 6:4); $[\alpha]_D^{20}$ +4.50 (*c* 1.0, $CHCl_3$); **IR** ν_{max} 2930, 1738, 1664, 1462, 1262, 1173, 1072, 964, 838, 805, 777, 744; **^{1}H NMR** (500 MHz, $CDCl_3$) δ_H 6.41 – 6.31 (2H, m, H14, H26), 6.23 – 6.13 (2H, m, H24, H25), 6.03 (1H, dd, J = 11.0, 11.0 Hz, H27), 5.98 (1H, dd, J = 11.2, 11.2 Hz, H13), 5.67 (1H, dd, J = 14.0, 7.4 Hz, H23), 5.64 (1H, dd, J = 15.3, 5.4, H15), 5.42 – 5.36 (2H, m, H12, H30), 5.31 (1H, dd, J = 10.3, 10.3 Hz, H28), 5.06 (1H, d, J = 10.0, H9), 4.69 – 4.62 (1H, m, H16), 4.51 – 4.29 (8H, m, H11, H17a, H22, H34, $(OCH_2CF_3)_2$), 3.95 (2H, s, H7), 3.91 (1H, dd, J = 7.9, 7.9 Hz, H17b), 3.86 (1H, ddd, J = 8.8, 8.8, 2.8, H32), 3.76 – 3.68 (2H, m, H20, OH7), 3.36 (3H, s, OMe20), 3.05 (2H, d, J = 20.6 Hz, H2), 2.99 – 2.87 (2H, m, H19, H29), 2.67 – 2.59 (1H, m, H10), 1.90 – 1.80 (2H, m, H31, H33a), 1.72 (3H, s, Me8), 1.66 – 1.60 (1H, m, H33b), 1.54 – 1.49 (2H, m, H21a, H21b), 1.29 (3H, d, J = 6.5 Hz, H35), 1.15 (3H, d, J = 6.8 Hz, Me19), 1.02 – 0.85 (45H, m, Me10, Me29, Me31, Si(*t*Bu)$_2$, Si*t*BuMe$_2$, $Si(CH_2CH_3)_3$), 0.56 (6H, q, J = 7.9 Hz, $Si(CH_2CH_3)_3$), 0.06 (3H, s, Si*t*BuMe$_2$), 0.00 (3H, s, Si*t*BuMe$_2$); **^{13}C NMR** (125 MHz, $CDCl_3$) 170.5, 164.1 (d, J = 3.8 Hz), 138.2, 135.7, 133.8, 133.7, 133.3, 132.8, 129.3, 129.3, 128.9, 127.2, 27.2, 126.9, 122.5 (qd, J = 277, 8.6 Hz), 122.5 (qd, J = 278, 8.3 Hz), 78.1, 78.1, 72.7, 71.7, 70.0, 69.6, 69.1, 67.3, 66.0, 62.5 (m), 57.1, 40.9, 40.1, 37.7, 35.7, 35.0, 33.8 (d, J = 145 Hz), 27.4, 27.1, 25.9, 23.6, 21.1, 20.7, 18.1, 17.5, 14.3, 13.9, 11.2, 9.5, 6.9, 4.9, −3.8, −4.9; **^{19}F NMR** (470 MHz, $CDCl_3$) δ_F −75.42 (3F, t, J = 8.0 Hz), −75.48 (3F, t, J = 8.0 Hz); **^{31}P NMR** (202 MHz, $CDCl_3$) δ_P 23.81; **HRMS** ESI calc. for $C_{61}H_{107}F_6NO_{12}PSi_3$ $[M+H]^+$ 1274.6737, found 1274.6739.

g. Endgame

Vinyl stannane S11

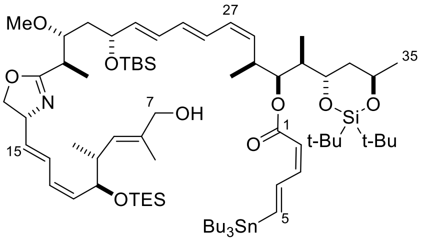

To a solution of phosphonate **28** (20.0 mg, 15.7 µmol) in THF (4.0 mL) at 0 °C was added sodium hydride (6.0 mg, 250 µmol). After 30 min, the reaction mixture was cooled to –78 °C and aldehyde **21** (20.0 mg, 58.8 µmol) in THF (1 mL) was added. After stirring for 48 h, the reaction mixture was quenched with water (5 mL) and extracted with EtOAc (3 × 10 mL). The organic layers were dried (Na_2SO_4) and concentrated *in vacuo*. Purification by flash chromatography (Et_2O/PE 1:9) gave vinyl stannane **S11** (17.1 mg, 12.6 µmol, 80%) as a colourless oil as a 2.5:1 mixture of 2*Z*:2*E* isomers.

Rf 0.8 (PE / EtOAc 6:4); $[\alpha]_D^{20}$ +9.97 (*c* 1.1, $CHCl_3$); **IR** ν_{max} 2929, 1716, 1663, 1619, 1461, 1376, 1250, 1191, 1143, 1088, 1007, 837, 806, 776; **^{1}H NMR** (500 MHz, $CDCl_3$) δ_H 7.79 (0.66H, ddd, J =18.8, 10.6, 1.0 Hz, H4), 7.14 (0.33H, dd, J = 15.3, 10.3 Hz, H3*), 6.79 (0.33H, d, J = 18.7 Hz, H5*), 6.69 (0.66H, d, J = 18.7 Hz, H5), 6.63 (0.33H, dd, J = 18.5, 10.3 Hz, H4*), 6.45 – 6.33 (2.66H, m, H3, H14, H26), 6.20 – 6.08 (2H, m, H24, H25), 6.05 – 5.94 (2H, m, H13, H27), 5.79 (0.33H, d, J = 15.4 Hz, H2*), 5.67 – 5.59 (2H, m, H15, H23), 5.51 (0.66H, d, J = 11.4 Hz, H2), 5.46 (0.33H, dd, J = 10.5, 10.5 Hz, H28*), 5.42 (0.66H, dd, J = 10.5 Hz, H28), 5.39 (1H, dd, J = 11.1, 8.5 Hz, H12), 5.29 – 5.24 (1H, m, H30, H30*), 5.06 (1H, dd, J = 10.0, 1.1 Hz, H9), 4.66 (1H, m, H16), 4.45 (1H, ddd, J = 8.3, 4.4, 1.1 Hz, H11), 4.39 (1H, dqd, J = 6.4, 6.4, 2.3 Hz, H34), 4.35 – 4.29 (1H, m, H22), 4.32 (1H, dd, J = 10.2, 8.4 Hz, H17a), 4.00 – 3.90 (1H, m, H32), 3.95 (2H, s, H7), 3.91 (1H, dd, J = 7.8, 7.8 Hz, H17b), 3.79 –

3.67 (2H, m, H20, OH7), 3.36 (3H, s, OMe20), 2.98 – 2.90 (1H, m, H29), 2.90 (1H, qd, J = 6.9, 5.5 Hz, H19), 2.63 (1H, dqd, J = 9.6, 6.8, 3.8 Hz, H10), 1.96 – 1.83 (2H, m, H31, H33a), 1.72 (3H, d, J = 1.1 Hz, Me8), 1.56 – 1.46 (9H, m, H21a, H21b, H33b, $Sn(CH_2CH_2\underline{CH_2}CH_3)_3$), 1.36 – 1.23 (9H, m, H35, $Sn(CH_2\underline{CH_2}CH_2CH_3)_3$), 1.15 (3H, d, J = 7.1 Hz, Me19), 1.02 – 0.86 (60H, Me10, Me29, Me31, $Sn(\underline{CH_2}CH_2CH_2\underline{CH_3})_3$, $Si(\underline{tBu})_2$, $Si\underline{tBu}Me_2$, $Si(CH_2\underline{CH_3})_3$), 0.56 (6H, q, J = 7.9 Hz, $Si(CH_2\underline{CH_3})_3$), 0.07 (3H, s, $SitBu\underline{Me_2}$), 0.01 (3H, s, $SitBu\underline{Me_2}$); **^{13}C NMR** (125 MHz, $CDCl_3$) 178.6, 167.0*, 165.5, 147.3, 146.8*, 146.4, 146.2*, 144.4*, 142.8, 137.2*, 137.4, 135.7, 134.0, 133. 132.8, 132.7, 129.8, 129.0, 128.9, 127.8, 127.6*, 127.2, 126.9, 120.2*, 116.2, 78.2, 76.0, 75.4, 72.7, 71.7, 70.2, 69.6*, 69.4, 69.1, 67.6, 66.0, 57.2, 41.6*, 41.4, 40.1, 40.1, 37.3, 35.8, 35.5*, 35.2, 29.1, 29.0*, 27.4, 27.3, 27.1, 25.9, 23.6, 21.3, 20.7, 18.1, 18.0*, 17.8, 14.3, 14.1, 13.9, 13.7, 11.4, 9.7, 9.6*, 6.9, 4.9, -3.6, -4.8; **HRMS** ESI calc. for $C_{72}H_{132}NO_9Si_3{}^{112}Sn$ $[M+H]^+$ 1350.8253, found 1350.8276.

*Identifiable resonances for the minor Δ2-*E* isomer.

Aldehyde 29

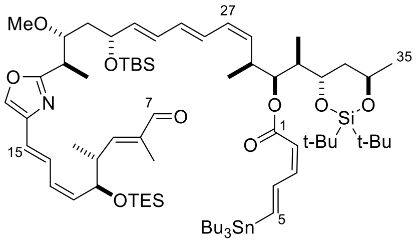

Oxazoline **S11** (8.0 mg, 5.9 µmol) was dissolved in benzene (1.0 mL) and 4Å molecular sieves (50 mg) were added. Activated manganese dioxide (5 x 30 mg portions) was added every 30 min until the reaction was judged complete by TLC analysis (2.5 h total). The reaction mixture was filtered through Celite and concentrated *in vacuo*. Purification by preparative thin layer chromatography (PE / EtOAc, 10:1) afforded the pure *Z*-isomer **29** (1.6 mg, 1.2 µmol, 20%) along with a 1:1 mixture of *E* and *Z* isomers (1.0 mg, 0.75 µmol, 13%).

Rf 0.7 (PE / EtOAc 4:1); $[\alpha]_D^{20}$ 5.90 (*c* 0.16, $CHCl_3$); **IR** ν_{max} 2927, 1716, 1691, 1457, 1377, 1246, 1093, 1038, 1006, 826, 742; **^{1}H NMR** (500 MHz, $CDCl_3$) δ_H 9.42 (1H, s, H7), 7.81 (1H, dd, J = 18.8, 10.6 Hz, H4), 7.52 (1H, s, H17), 7.12 (1H, dd, J = 15.0, 11.8 Hz, H14), 6.71 (1H, d, J = 19.0 Hz, H5), 6.51 (1H, d, J = 10.0 Hz, H9), 6.47 – 6.38 (2H, m, H3, H26), 6.38 (1H, d, J = 15.2 Hz, H15), 6.21 – 6.09 (3H, m, H13, H24, H25), 6.01 (1H, dd, J = 11.1, 11.1 Hz, H27), 5.61 (1H, dd, J = 14.3, 7.4 Hz, H23), 5.52 (1H, d, J = 11.5 Hz, H2), 5.48 – 5.38 (2H, m, H12, H28), 5.29 (1H, dd, J = 7.1, 4.1 Hz, H30), 4.66 (1H, dd, J = 8.9, 4.5 Hz, H11), 4.41 (1H, dqd, J = 6.6, 6.6, 1.5 Hz, H34), 4.33 (1H, ddd, J = 9.5, 9.5, 2.5 Hz, H22), 3.97 (1H, ddd, J = 8.2, 8.2, 1.5 Hz, H32), 3.86 – 3.76 (1H, m, H20), 3.42 – 3.35 (1H, m, H19), 3.40 (3H, s, OMe20), 2.94 (1H, ddq, J = 9.8, 6.8, 6.8 Hz, H29), 2.89 – 2.80 (1H, m, H10), 1.97 – 1.84 (2H, m, H31, H33a), 1.76 (3H, s, Me8), 1.57 – 1.48 (9H, m, H21a, H21b, H33b, $Sn(CH_2CH_2\underline{CH_2}CH_3)_3$), 1.376 – 1.28 (12H, m, H35, Me10, $Sn(CH_2\underline{CH_2}CH_2CH_3)_3$), 1.10 (3H, d, J = 7.1 Hz, Me19), 1.04 – 0.86 (57H, Me29, Me31, $Sn(\underline{CH_2}CH_2CH_2\underline{CH_3})_3$, $Si(\underline{tBu})_2$, $Si\underline{tBu}Me_2$, $Si(CH_2\underline{CH_3})_3$), 0.59 (6H, q, J = 8.0 Hz, $Si(CH_2\underline{CH_3})_3$), 0.08 (3H, s, $SitBu\underline{Me_2}$), 0.02 (3H, s, $SitBu\underline{Me_2}$); **^{13}C NMR** (125 MHz, $CDCl_3$) 195.6, 166.0, 165.5, 156.8, 147.4, 146.4, 142.8, 139.3, 138.7, 137.3, 135.4, 134.1, 133.8, 132.6, 129.8, 129.1, 128.9, 127.9, 124.9, 122.5, 116.2, 79.1, 75.3, 71.8, 70.3, 69.4, 67.6, 57.4, 41.4, 41.1, 40.2, 37.3, 36.0, 35.2, 29.1, 27.4, 27.3, 27.1, 25.9, 23.5, 21.3, 20.7, 18.1, 17.8, 16.1, 13.7, 11.5, 9.8, 9.7, 9.5, 6.8, 5.0, −3.7, −4.8; **HRMS** ESI calc. for $C_{72}H_{128}NO_9Si^{122}Sn$ $[M+H]^+$ 1346.7940 found 1346.7935.

Chivosazole F (**1**)

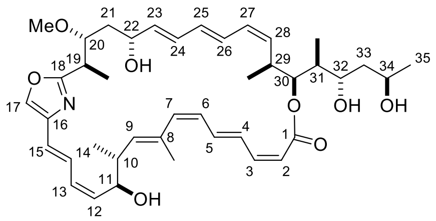

To a suspension of $[PPh_3CH_2I][I]$ (10.0 mg, 18.9 µmol) in THF (300 µL) was added sodium hexamthyldisilazide solution (0.63 M in THF, 30.0 µL, 18.9 µmol) to form a clear, orange solution. The solution was cooled to −78 °C and a solution of aldehyde **29** (1.6 mg, 1.2 µmol) in THF (300 + 100 µL) was added *via* cannula. The reaction mixture was stirred at −78 °C for 1 h before being diluted with hexane (1 mL) and warmed to rt. The reaction mixture was filtered through a short plug of silica gel and concentrated *in vacuo* to yield the vinyl iodide which was used directly without further purification.

A stock solution of $Pd(PPh_3)_4$ (3.2 mg, 2.7 µmol), CuTC (5.0 mg, 26.2 µmol) and $[Ph_2PO_2][NBu_4]$ (12.5 mg, 27.2 µmol) was prepared in DMF (1.0 mL). A portion of this solution (100 µL) was transferred to a flask, diluted with DMF (2.0 mL), cooled to 0 °C and placed in the dark. A solution of the crude vinyl iodide in DMF (2.0 mL) was added to the catalyst solution over 2.5 h *via* syringe pump and the reaction mixture stirred for 30 min following completion of the addition. The reaction mixture was filtered through a plug of silica (eluting with Et_2O), concentrated *in vacuo* and filtered again through a plug of silica (eluting with PE / Et_2O, 4:1). The solution of crude macrocycle was concentrated *in vacuo*, transferred to a plastic reaction vessel and submitted directly to the subsequent deprotection.

A stock solution of HF·pyridine was prepared from HF·pyridine (100 µL), pyridine (150 µL) and THF (150 µL) and stirred for 30 min at rt. A portion of this solution (100 µL) was added to the crude macrocycle and the reaction stirred overnight at rt. The reaction mixture was carefully quenched with $NaHCO_3$ solution (100 µL) and filtered through a plug of Celite (washing with EtOAc). The resulting mixture was concentrated *in vacuo* and purified by preparative thin layer chromatography (EtOAc / PE / MeOH, 6:3:1) to afford chivosazole F as a white amorphous solid (340 µg, 0.49 µmol, 41%).

$[\alpha]_D^{20}$ = – 3.3 (*c* 0.03, MeOH), *cf lit*[6] $[\alpha]_D^{20}$ = – 5.0 (*c* 0.2, MeOH); **^{1}H NMR** (700 MHz, CD_3OD) δ_H See **Table S1**; **^{13}C NMR** (175 MHz, CD_3OD) See **Table S1**; **HRMS** ESI calc. for $C_{41}H_{57}NO_8Na$ $[M+Na]^+$ 714.3982, found 714.3969.

3. Comparison of NMR Data for natural and synthetic chivosazole F

Table S1: Comparison of NMR data for natural[6] and synthetic chivosazole F (CD_3OD)

Carbon	*δ_C Natural / ppm 100 MHz*	δ_C Synthetic / ppm 175 MHz	*δ_H Natural / ppm 400 MHz*	*mult*	*J / Hz*	δ_H Synthetic / ppm 700 MHz	mult	J / Hz
1	*168.9*	168.9						
2	*117.9*	117.9	*5.43*	*d*	*11.5*	5.43	d	11.4
3	*145.4*	145.3	*6.51*	*dd*	*11.5, 11.5*	6.51	dd	11.7, 11.7
4	*130.6*	130.6	*7.07*	*dd*	*14.9, 11.8*	7.07	dd	14.9, 11.7
5	*139.8*	139.8	*6.88*	*dd*	*14.9, 11.0*	6.88	dd	14.9, 11.2
6	*129.0*	129.0	*5.99 – 5.92*	*m*		5.91	dd	11.3, 11.3
7	*139.9*	139.9	*5.85*	*d*	*11.3*	5.85	d	11.3
8	*134.3*	134.3						
9	*136.3*	136.3	*5.07*	*d*	*9.0*	5.08	d	8.9
10	*40.4*	40.4	*2.88 – 2.79*	*m*		2.83	ddq	9.0, 6.8, 6.8
11	*70.5*	70.5	*4.73*	*dd*	*9.0, 5.8*	4.73	dd	9.0, 5.8
12	*132.4*	132.4	*5.49*	*dd*	*10.1, 10.1*	5.50	dd	10.2, 10.2
13	*131.3*	131.3	*6.22*	*dd*	*11.2, 11.2*	6.22	dd	11.3, 11.3
14	*127.3*	127.3	*7.17*	*dd*	*15.2, 11.8*	7.17	dd	15.2, 11.7
15	*122.0*	122.0	*6.37*	*d*	*15.2*	6.37	d	15.2
16	*140.1*	140.1						
17	*137.7*	137.6	*7.73*	*s*		7.73	s	
18	*167.4*	167.4						
19	*36.4*	36.4	*3.54 – 3.48*	*m*		3.50	qd	6.9, 3.7
20	*80.0*	80.0	*3.94*	*ddd*	*10.4, 3.5, 1.4*	3.94	ddd	10.7, 3.8, 1.8
21	*39.6*	39.6	*1.71 – 1.65*	*m*		1.66	m	
			1.10 – 1.03	*m*		1.07	m	
22	*68.0*	68.1	*4.35*	*brd*	*9.3*	4.35	brd	10.5
23	*138.7*	138.7	*5.78*	*dd*	*15.2, 3.6*	5.77	dd	15.2, 3.6
24	*129.2*	129.2	*6.43 – 6.34*	*m*		6.39	ddd	15.0, 11.0, 1.8
25	*134.4*	134.4	*6.18*	*dd*	*14.7, 10.8*	6.18	dd	14.6, 10.8
26	*128.6*	128.6	*6.56*	*dd*	*14.7, 11.4*	6.55	dd	14.6, 11.4
27	*130.2*	130.2	*5.92 – 5.88*	*m*		5.96	dd	11.0, 11.0
28	*135.5*	135.5	*5.18*	*dd*	*10.6, 10.6*	5.17	dd	10.4, 10.4
29	*35.7*	35.7	*3.20-3.12*	*m*		3.16	ddq	10.1, 10.1, 6.3
30	*78.1*	78.1	*5.26*	*dd*	*10.3, 1.0*	5.26	dd	10.3, 1.0
31	*41.8*	41.8	*1.83 – 1.75*	*m*		1.80	m	
32	*70.3*	70.3	*3.47 – 3.43*	*m*		3.4	ddd	14.2, 6.7, 4.7
33	*44.7*	44.7	*1.65 – 1.60*	*m*		1.6	m	
			1.42 – 1.33	*m*		1.4	m	
34	*65.2*	65.2	*4.02*	*dqd*	*12.2[a], 6.2, 2.3*	4.02	dqd	9.2[a], 6.4, 2.3
35	*24.5*	24.4	*1.18*	*d*	*6.2*	1.18	d	6.3
Me 10	*14.3*	14.3	*1.05*	*d*	*6.8*	1.05	d	6.8
Me 19	*10.8*	10.9	*1.36*	*d*	*7.1*	1.36	d	7.1
Me 29	*17.8*	17.8	*1.02*	*d*	*6.7*	1.02	d	6.7
Me 31	*10.4*	10.4	*0.99*	*d*	*6.9*	0.99	d	6.9
Me 8	*17.2*	17.2	*1.91*	*s*		1.89	s	
OMe 20	*58.2*	58.2	*3.50*	*s*		3.49	s	

Full data and NMR spectra for an authentic sample of natural chivosazole F was disclosed by Kalesse when reporting his synthesis of chivosazole F and this data has been used for comparison.[6] [a]Upon inspection, the multiplets appear identical, however, we believe that the coupling constants may have been determined erroneously. Applying Hoye's protocol[8] gives coupling constants of *c*. 9, 6 and 2 Hz as recorded.

4 References

(1) Paterson, I.; Gibson, L. J.; Kan, S. B. J. *Org. Lett.* **2010**, *12*, 5530–5533.

(2) Soullez, D.; Plé, G.; Duhamel, L. *J. Chem. Soc. Perkin Trans. 1* **1997**.

(3) Garner, P.; Park, J. M. *Org. Synth.* **1992**, *70*, 18.

(4) Paterson, I.; Kan, S. B. J.; Gibson, L. J. *Org. Lett.* **2010**, *12*, 3724–3727.

(5) Ghosh, A. K.; Wang, Y.; Kim, J. T. *J. Org. Chem.* **2001**, *66*, 8973–8982.

(6) Brodmann, T.; Janssen, D.; Kalesse, M. *J. Am. Chem. Soc.* **2010**, *132*, 13610–13611.

(7) Jansen, R.; Irschik, H.; Reichenbach, H.; Höfle, G. *Liebigs Ann.* **1997**, *1997*, 1725–1732.

(8) Hoye, T. R.; Zhao, H. *J. Org. Chem.* **2002**, *67*, 4014–4016.

5. ^{1}H and ^{13}C NMR spectra

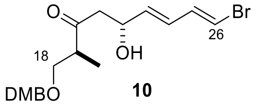

10

^{1}H NMR (500 MHz, $CDCl_3$)

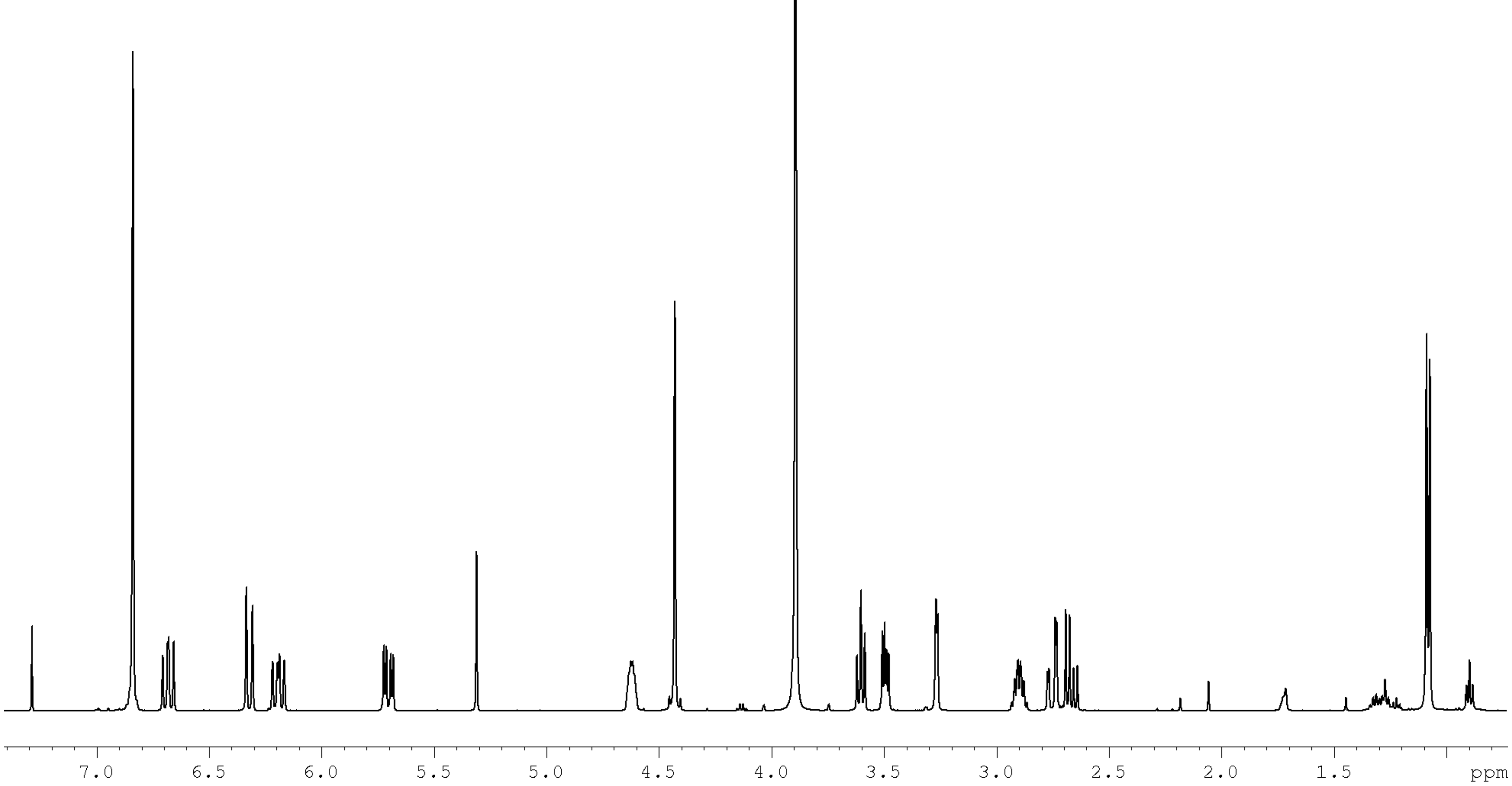

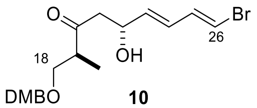

10

^{13}C NMR (125 MHz, $CDCl_3$)

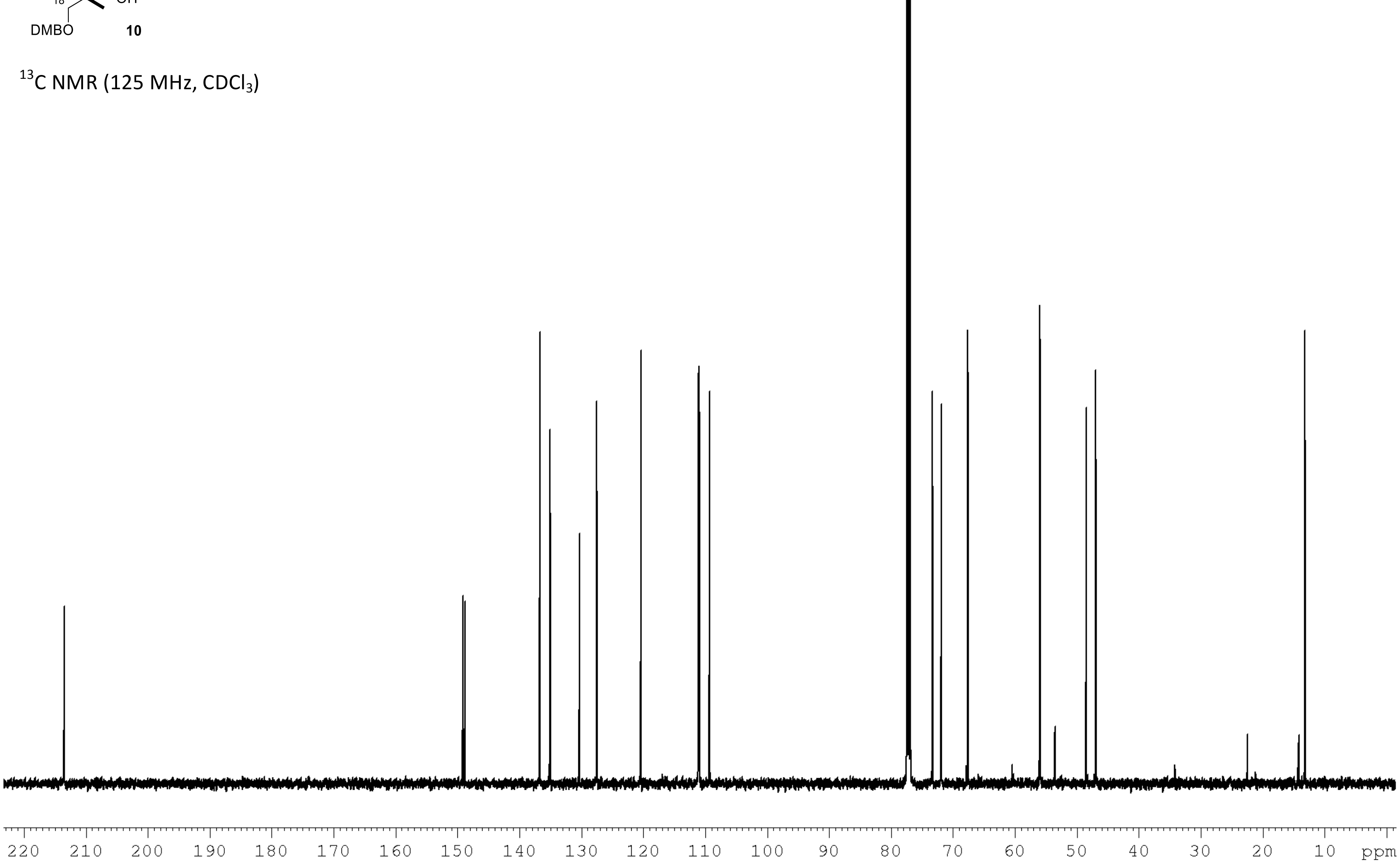

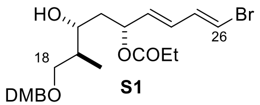

S1

^{1}H NMR (500 MHz, $CDCl_3$)

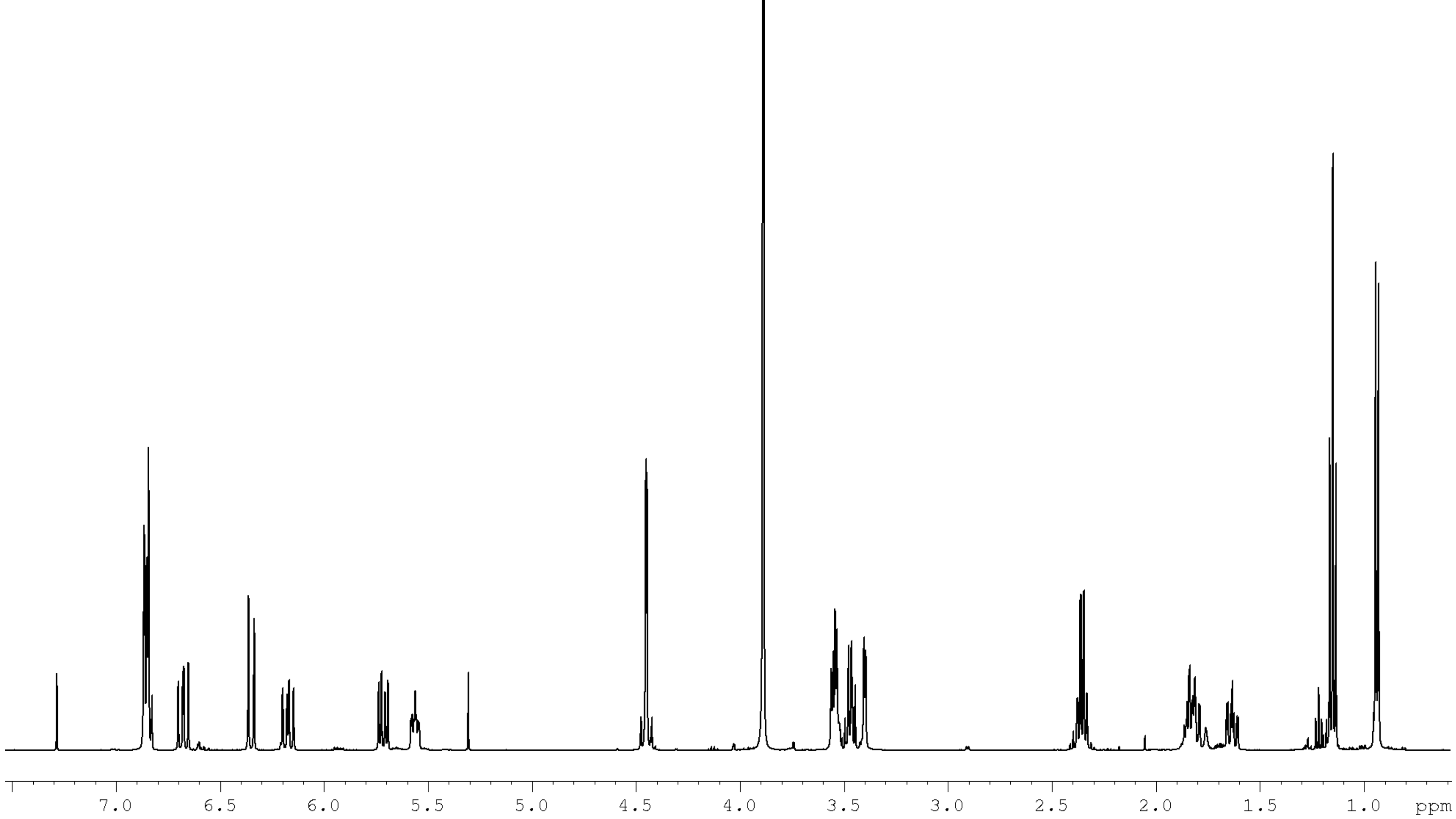

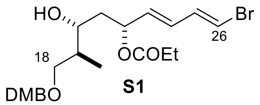

S1

^{13}C NMR (125 MHz, $CDCl_3$)

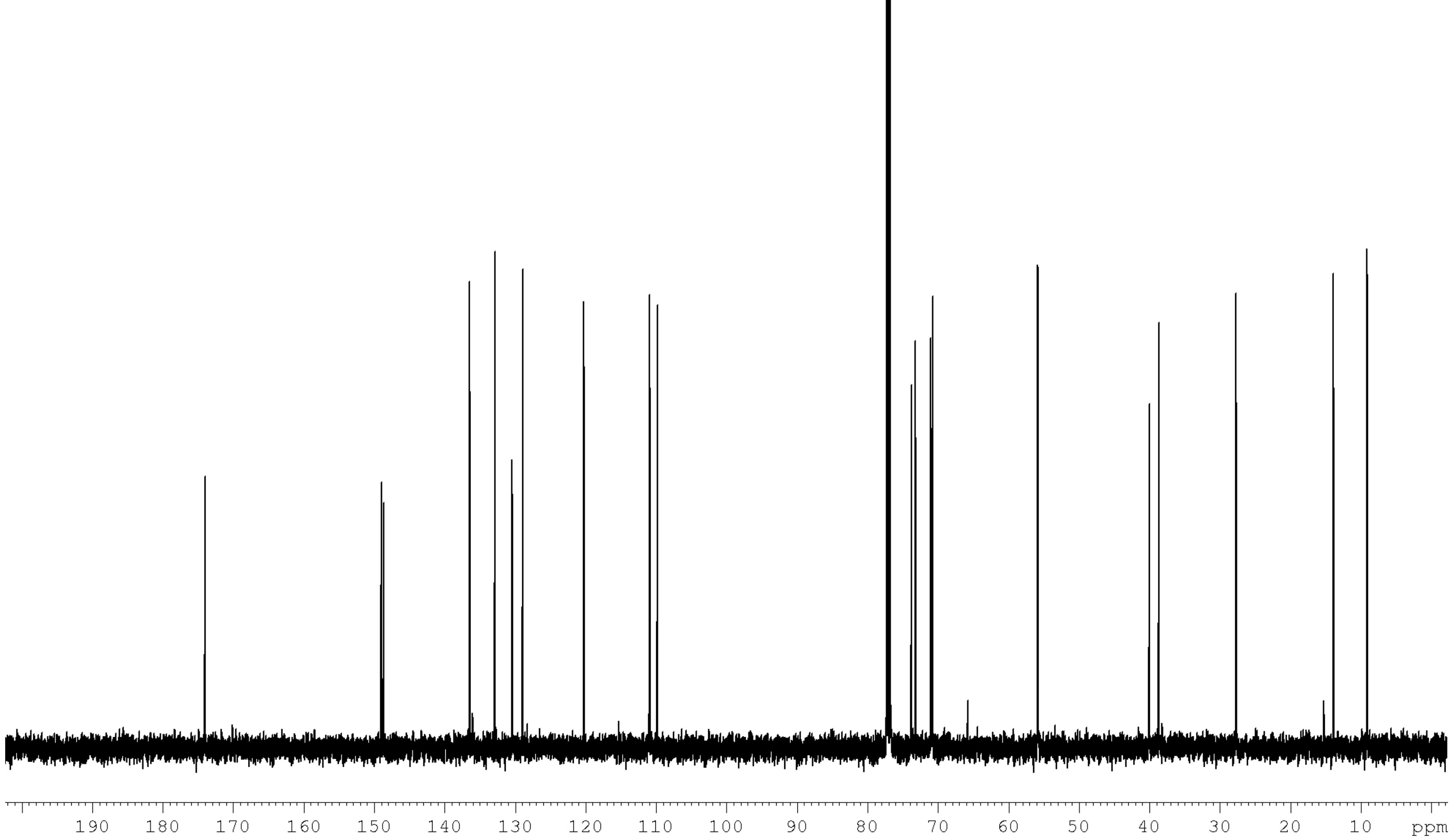

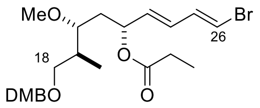

S2

^{1}H NMR (500 MHz, CDCl$_3$)

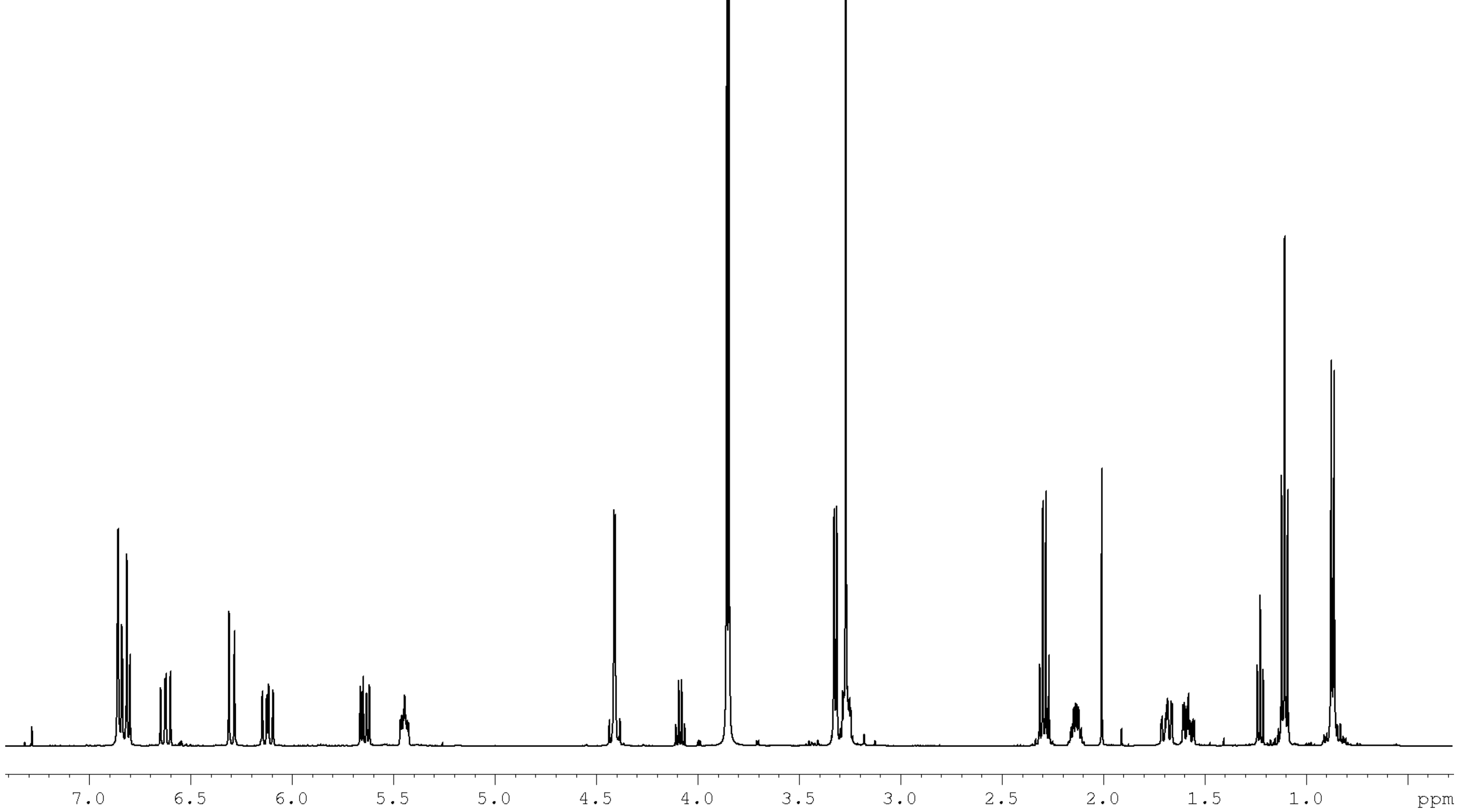

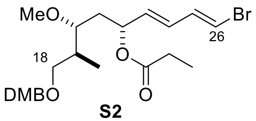

^{13}C NMR (125 MHz, $CDCl_3$)

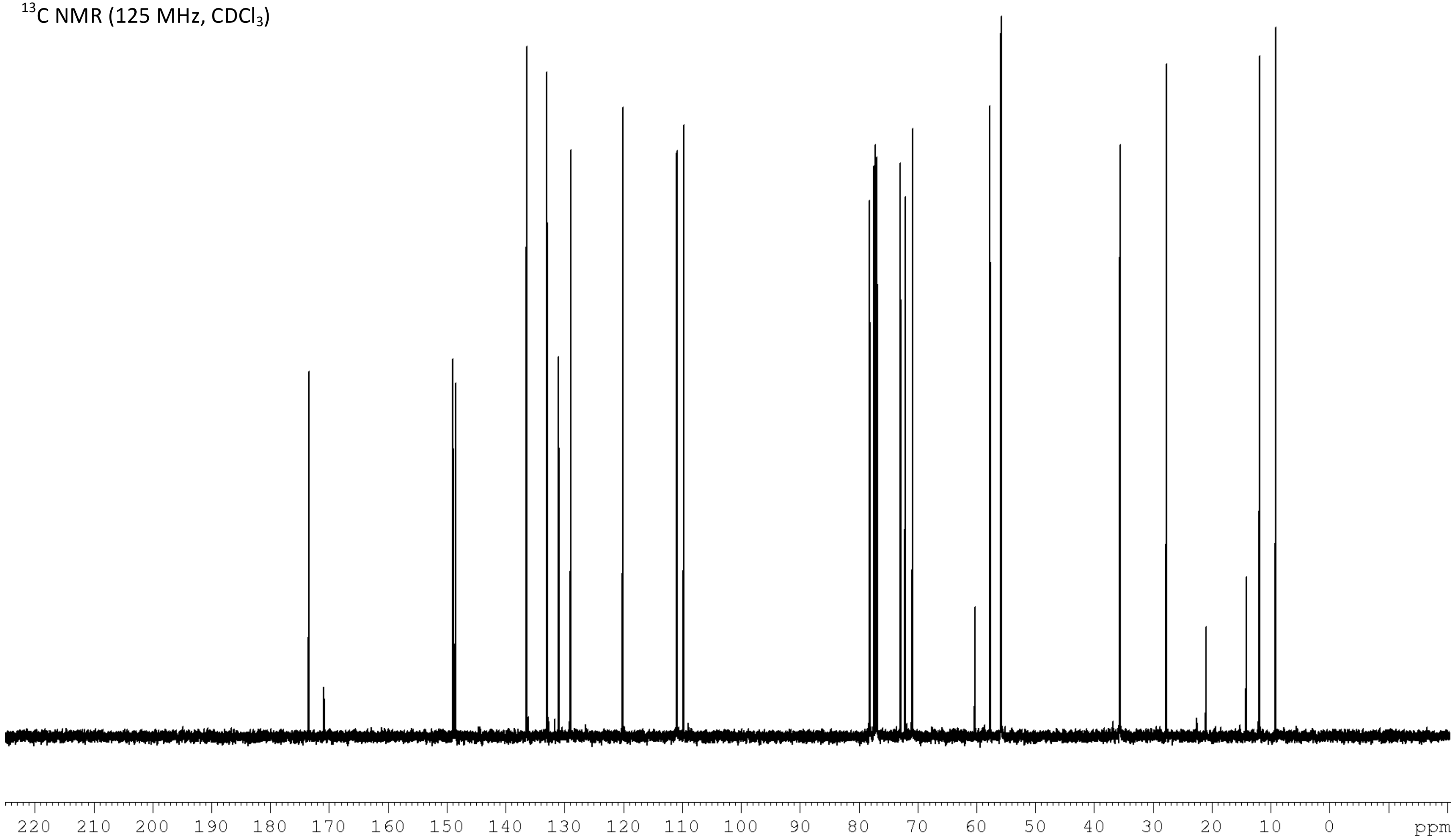

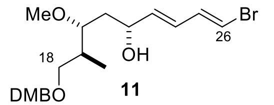

^{1}H NMR (400 MHz, $CDCl_3$)

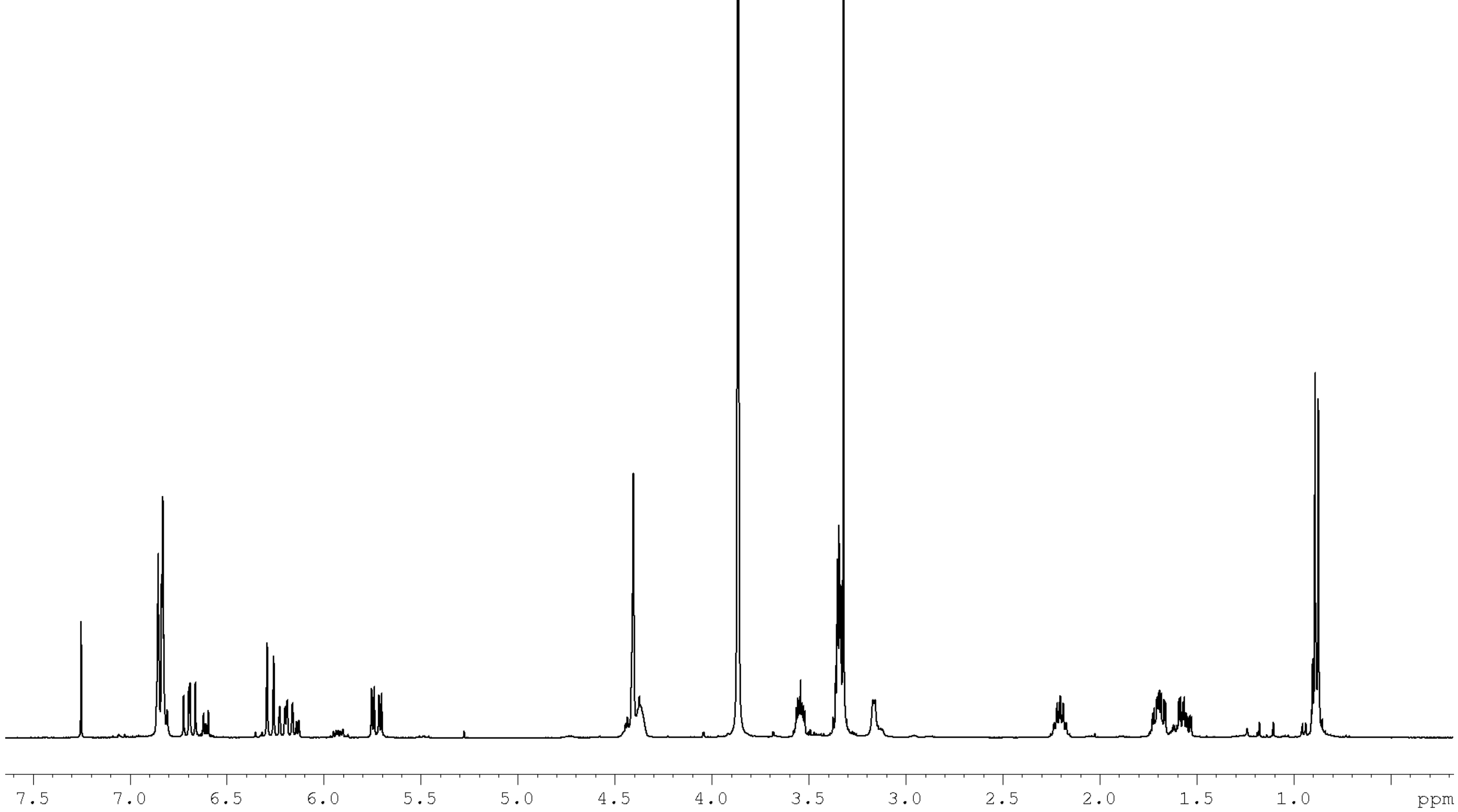

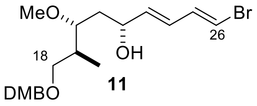

11

^{13}C NMR (100 MHz, $CDCl_3$)

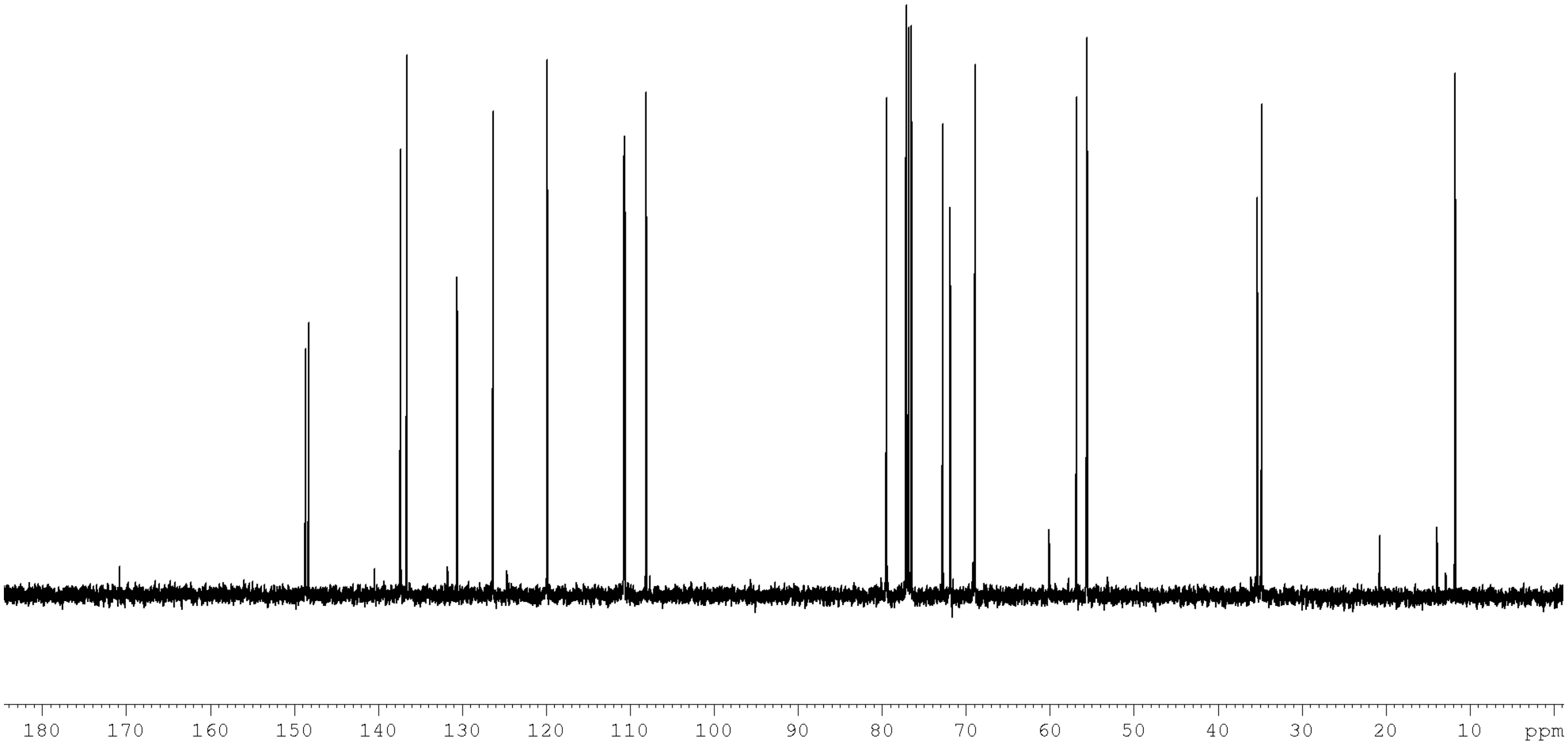

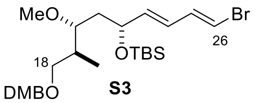

S3

^{1}H NMR (400 MHz, $CDCl_3$)

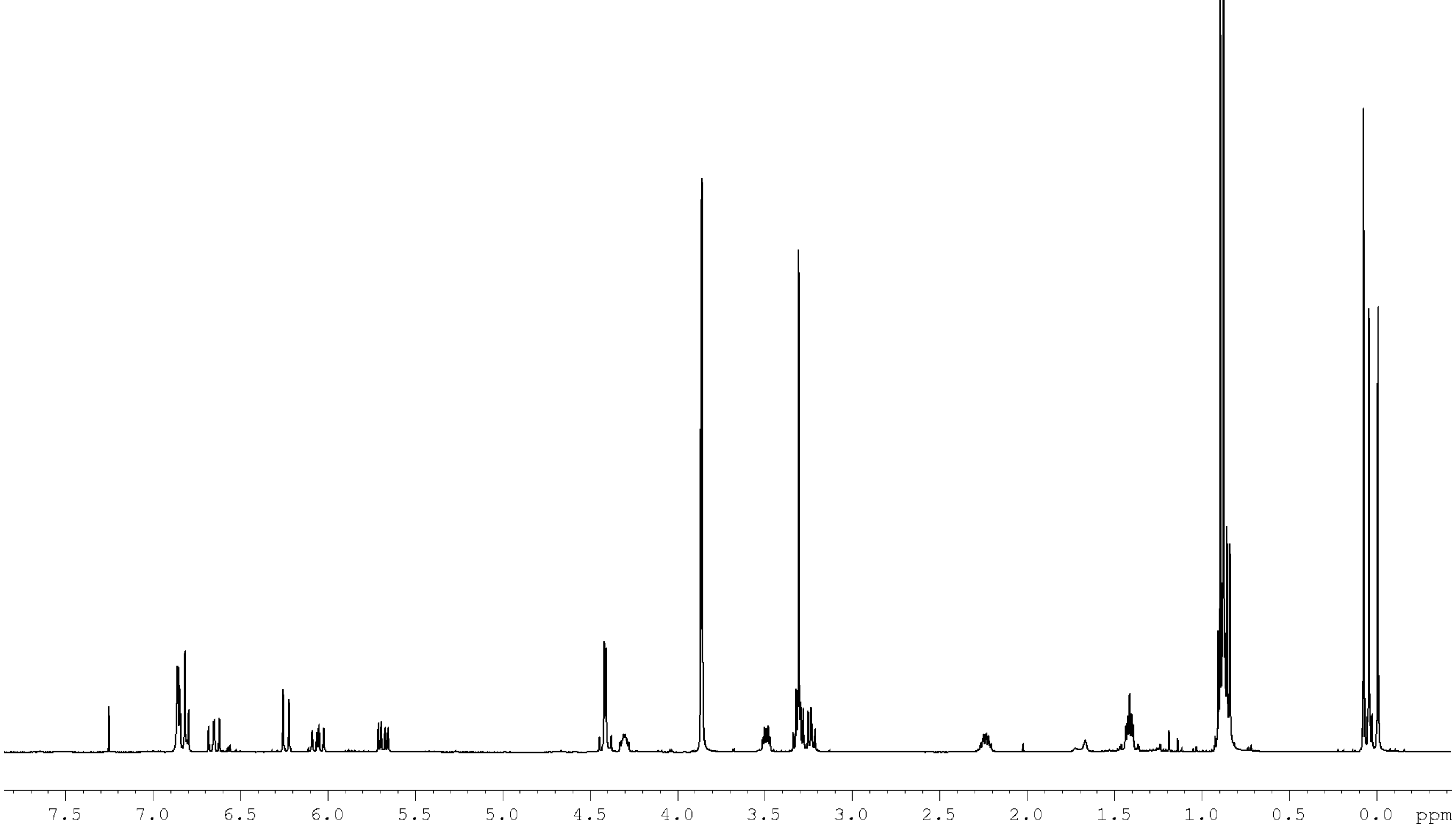

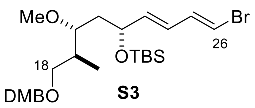

S3

^{13}C NMR (100 MHz, $CDCl_3$)

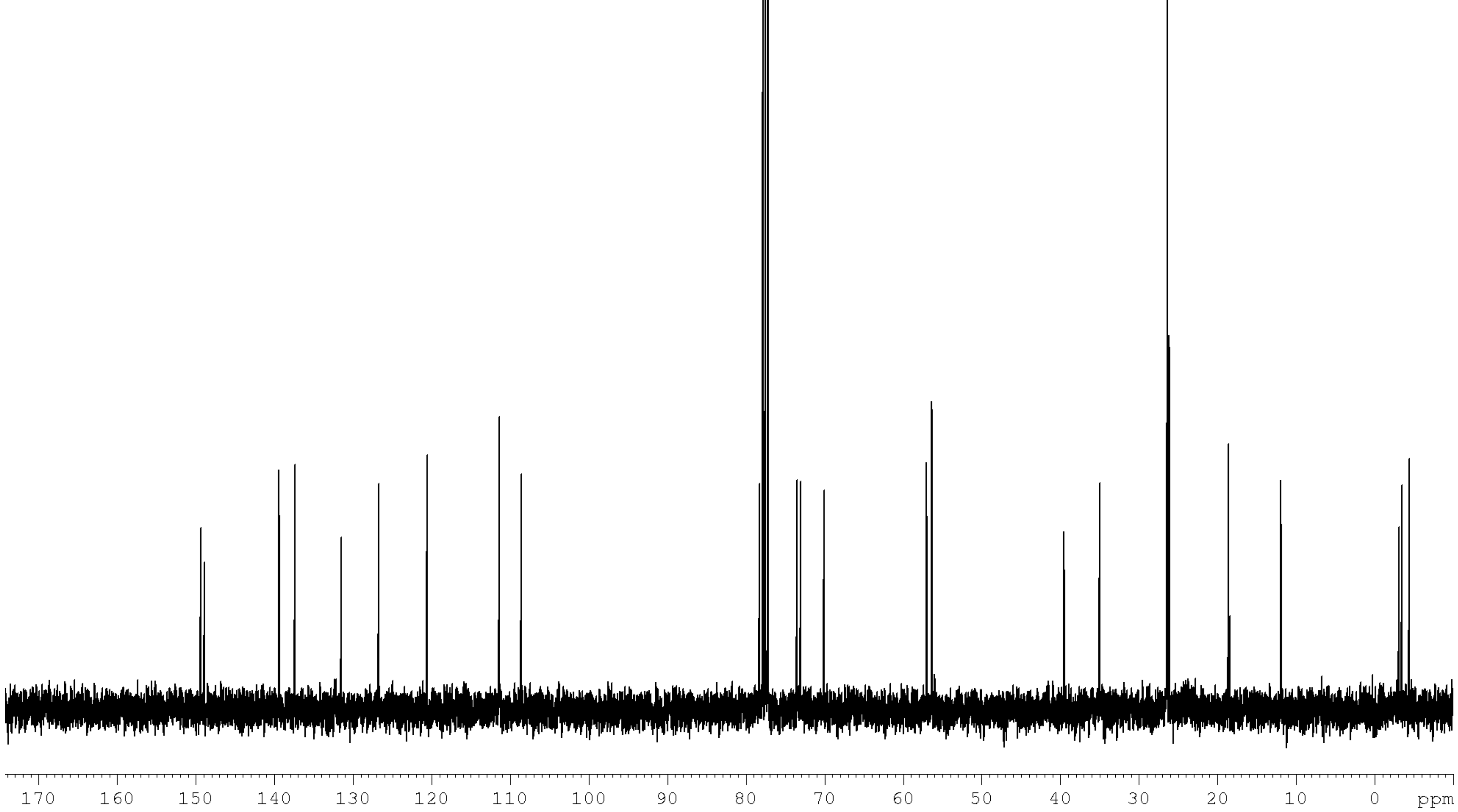

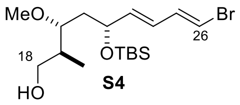

S4

^{1}H NMR (500 MHz, $CDCl_3$)

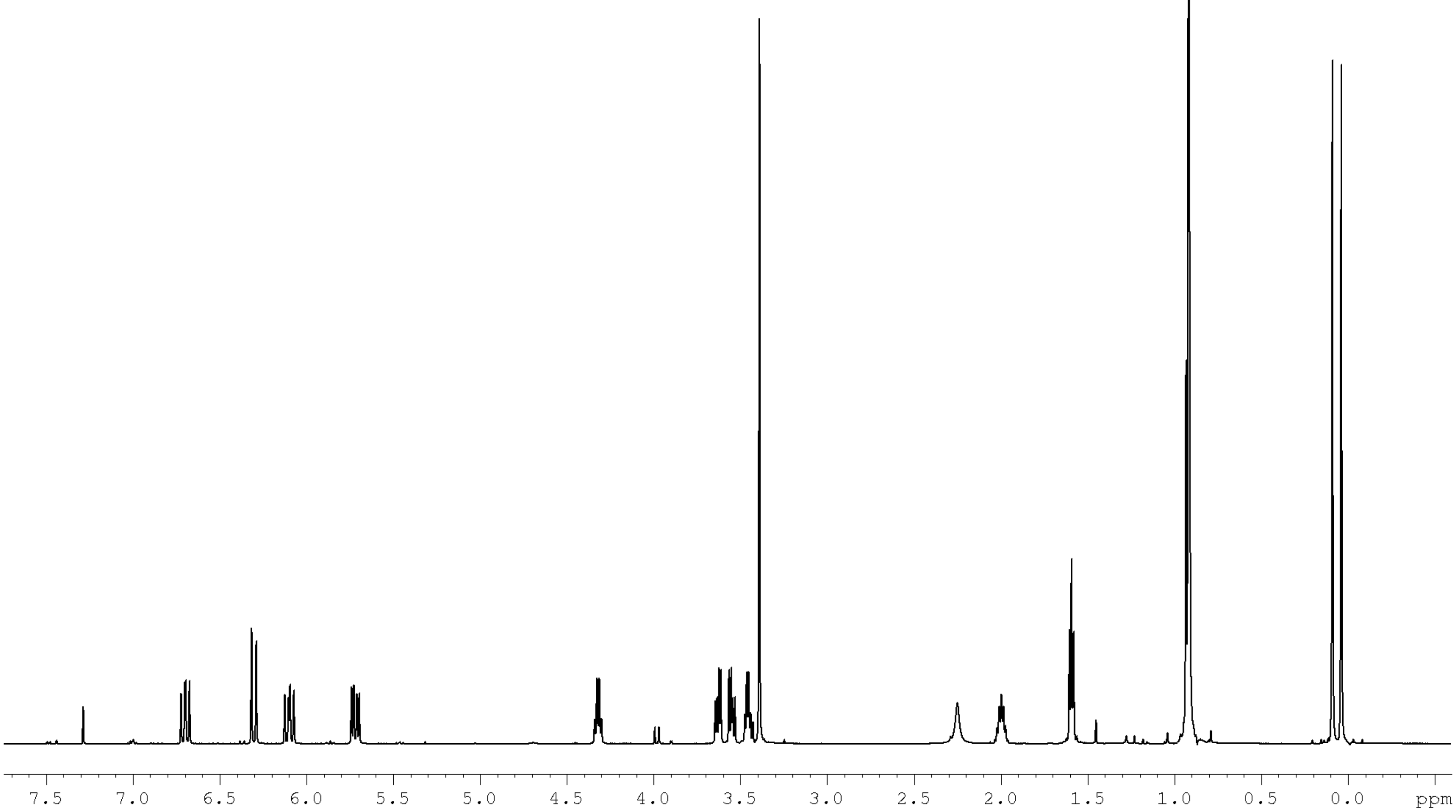

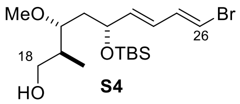

S4

^{13}C NMR (125 MHz, $CDCl_3$)

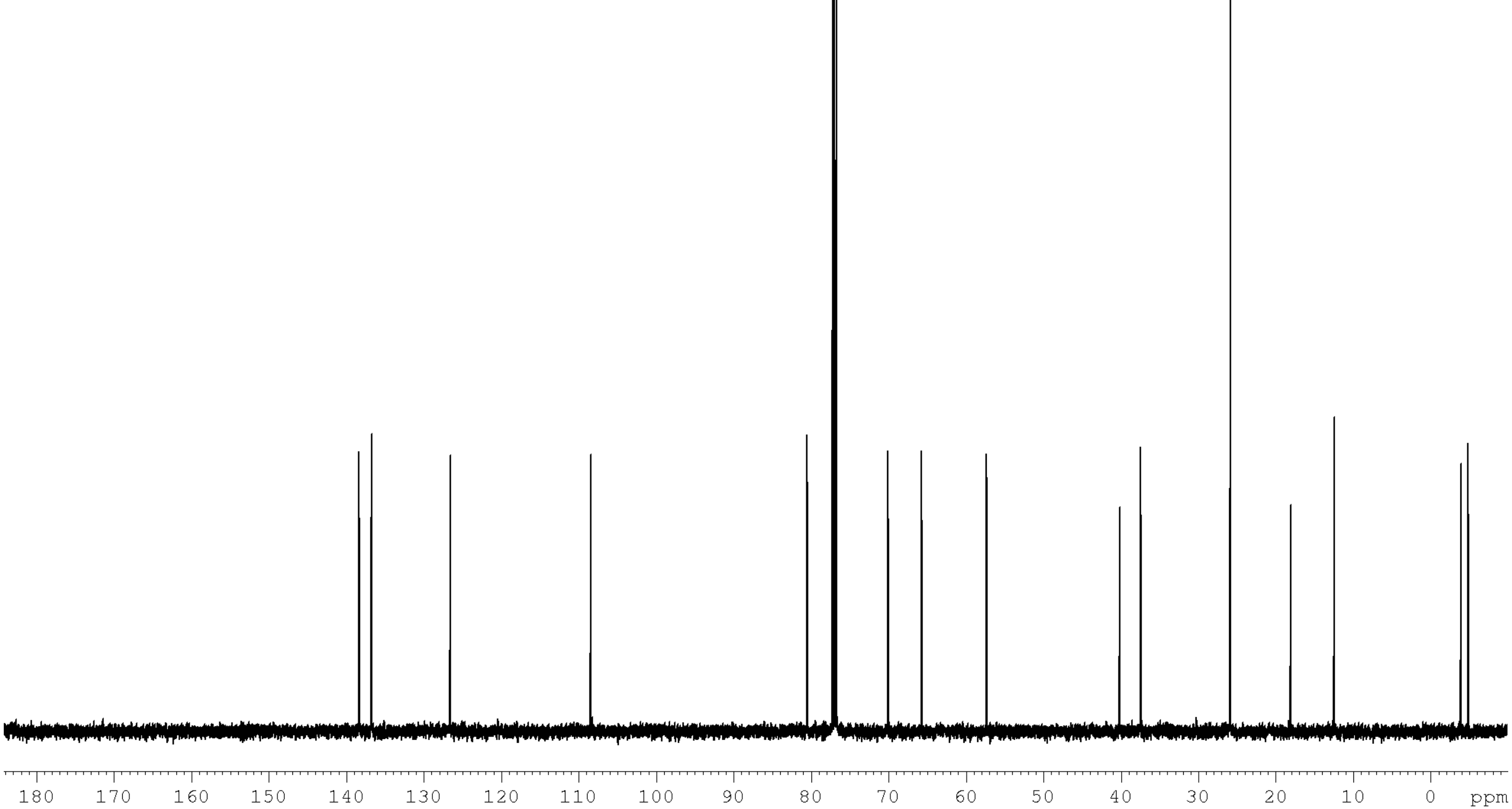

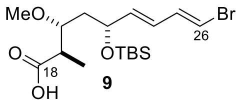

9

^{1}H NMR (500 MHz, $CDCl_3$)

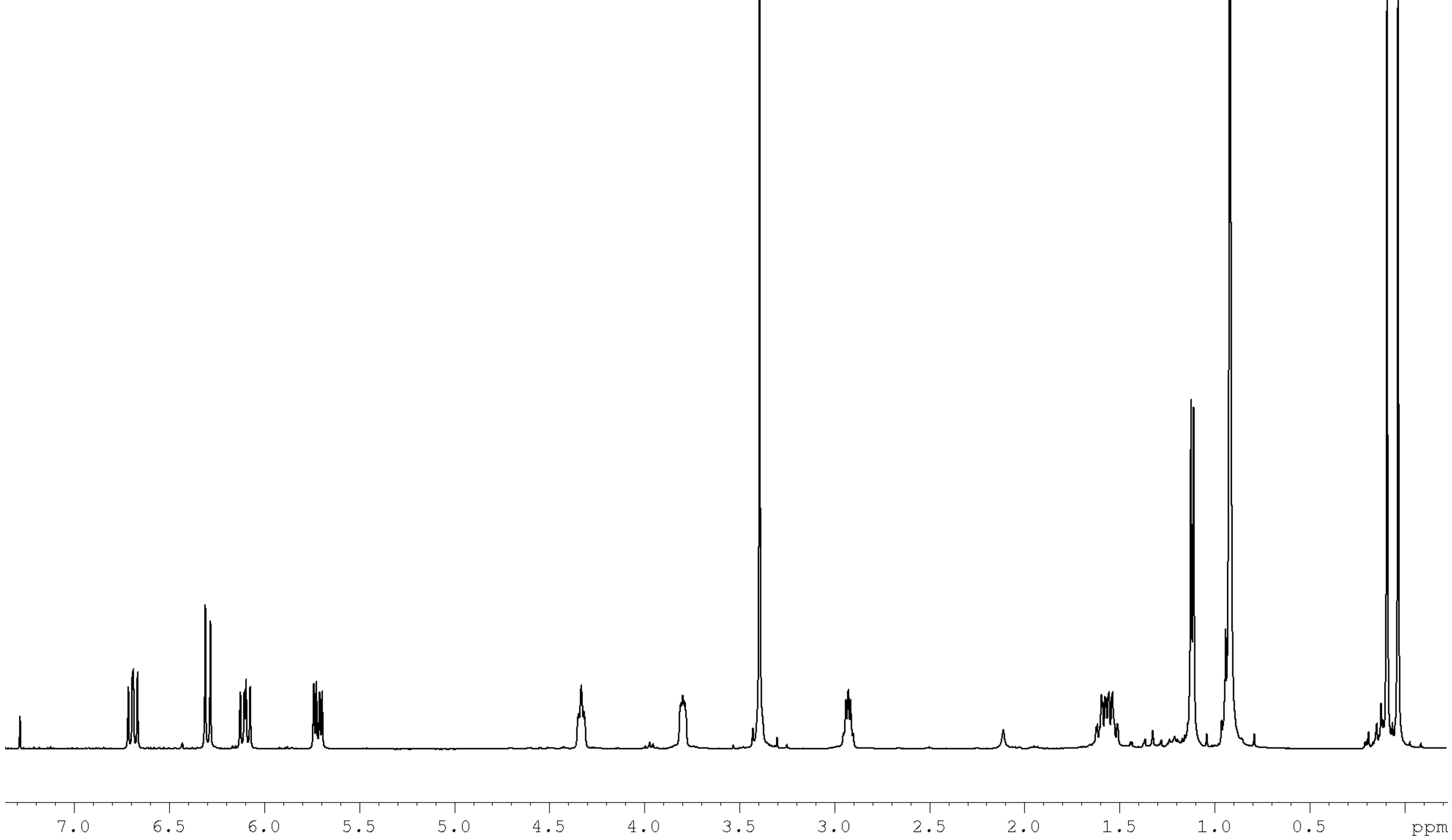

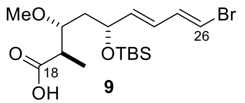

^{13}C NMR (125 MHz, $CDCl_3$)

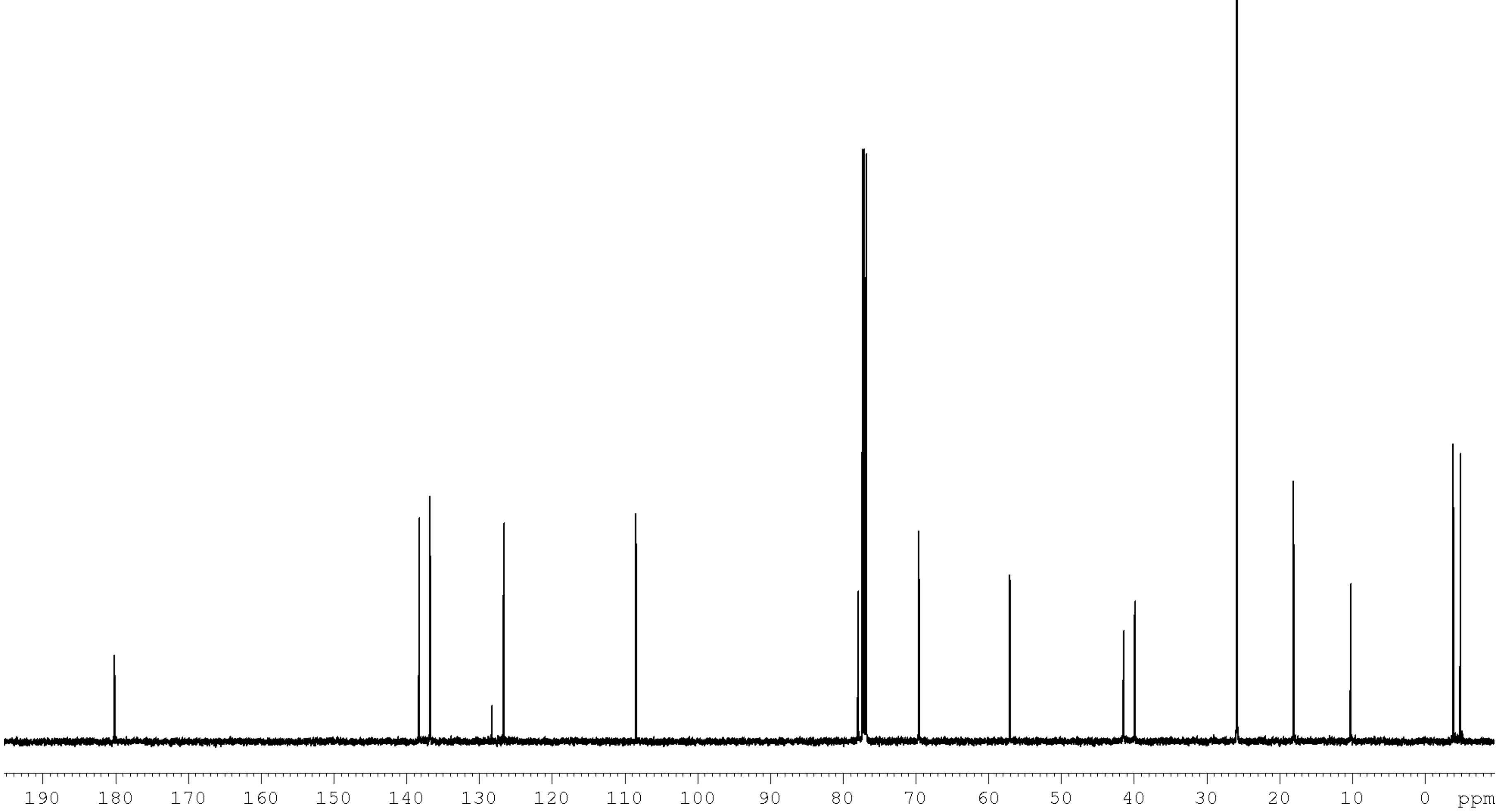

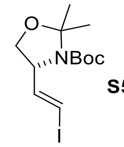
NBoc
O
I

S5

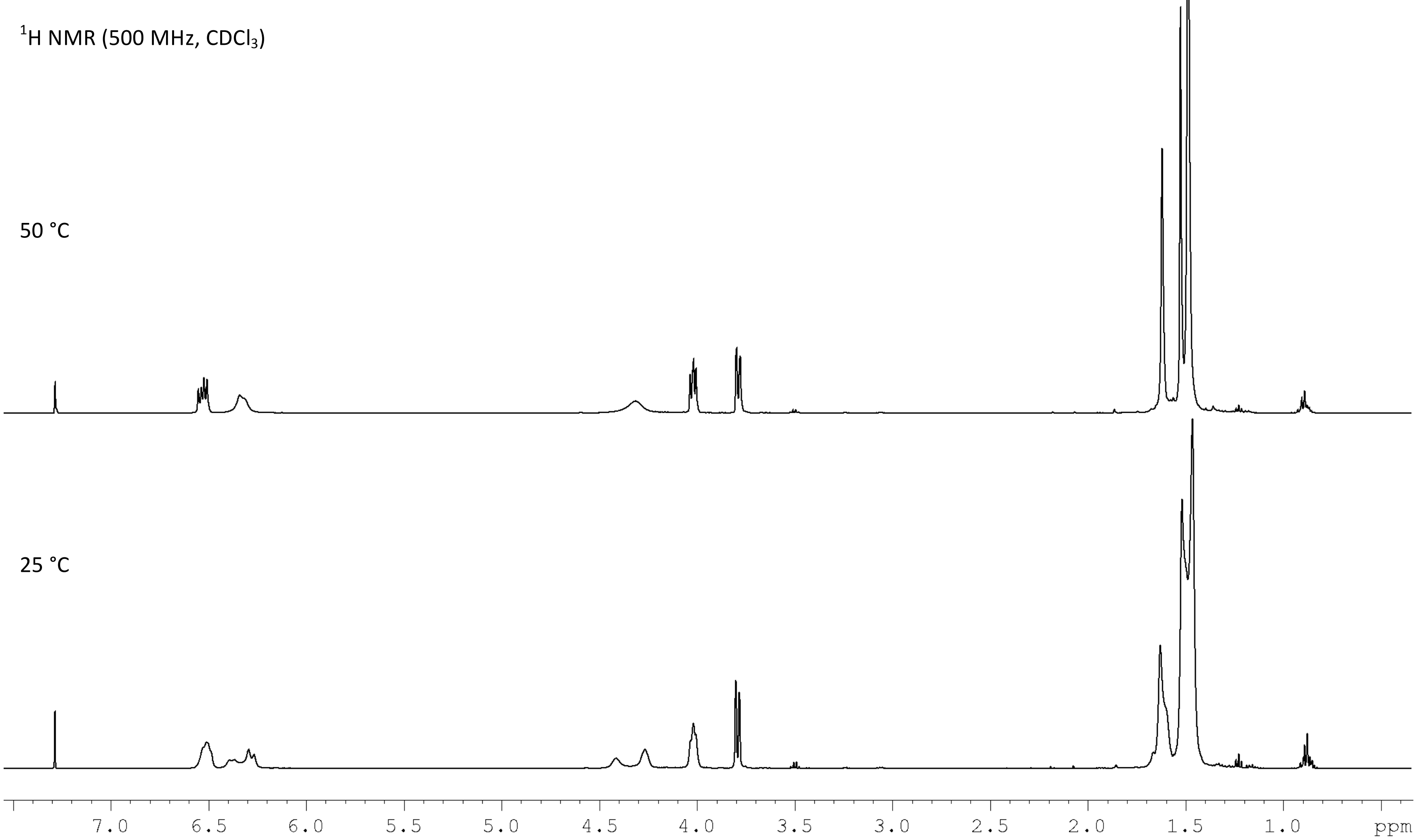
^{1}H NMR (500 MHz, $CDCl_3$)
50 °C
25 °C
7.0
6.5
6.0
5.5
5.0
4.5
4.0
3.5
3.0
2.5
2.0
1.5
1.0
ppm

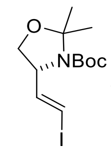

S5

^{13}C NMR (100 MHz, $CDCl_3$)

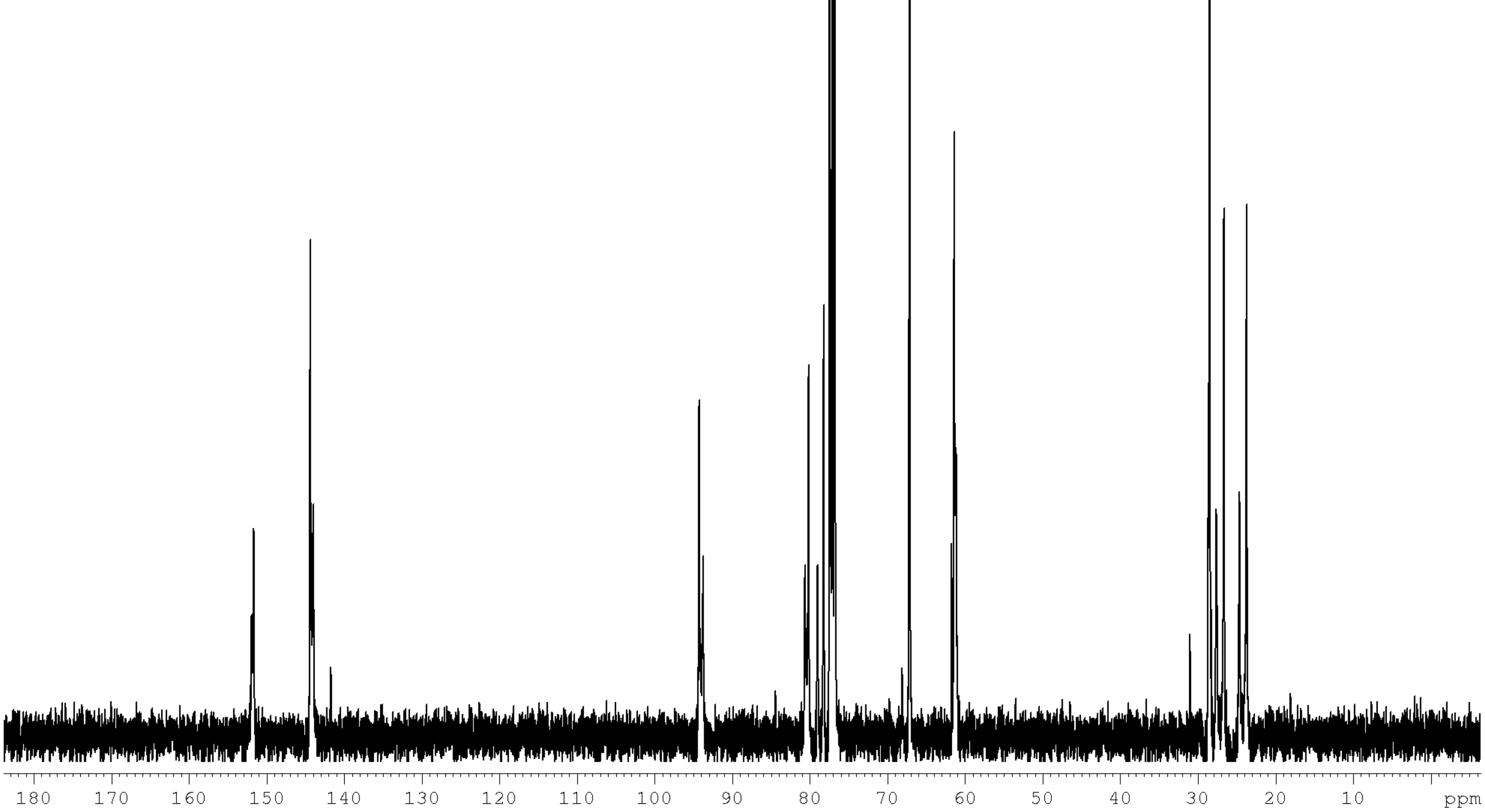

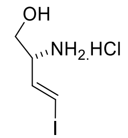

12

^{1}H NMR (500 MHz, CD_3OH)

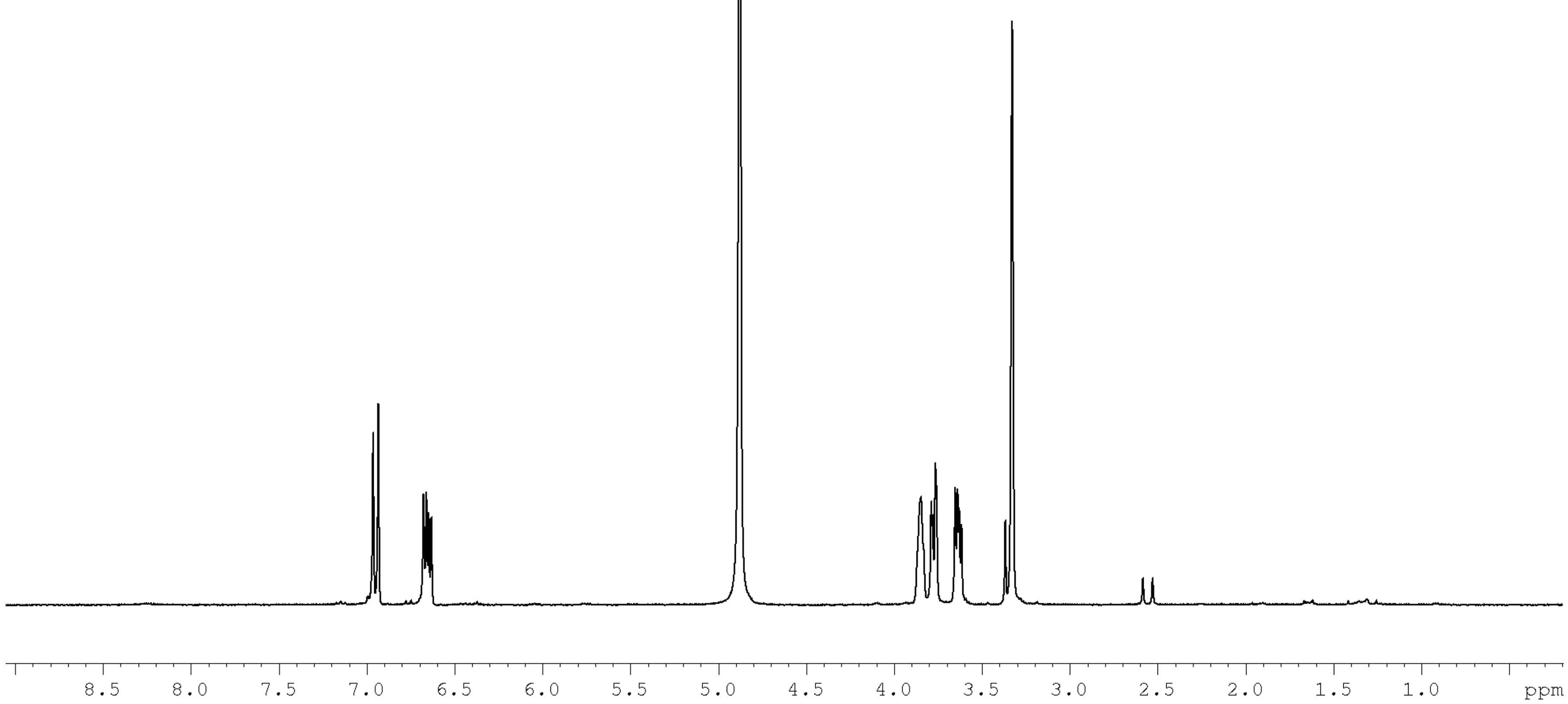

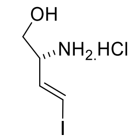

12

^{13}C NMR (125 MHz, CD_3OH)

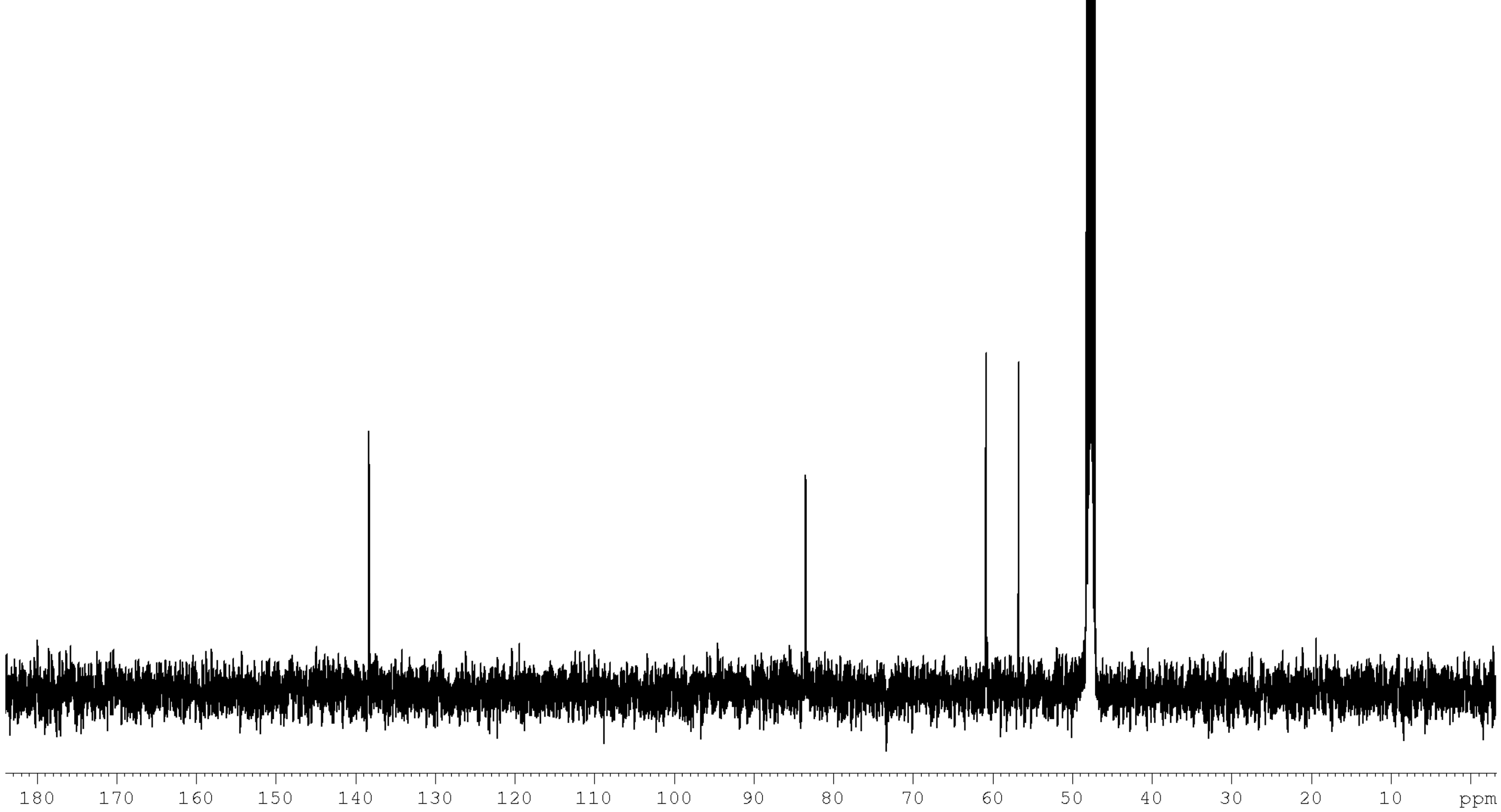

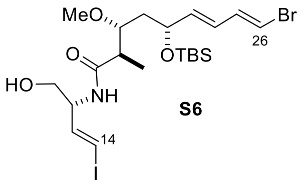

S6

^{1}H NMR (400 MHz, $CDCl_3$)

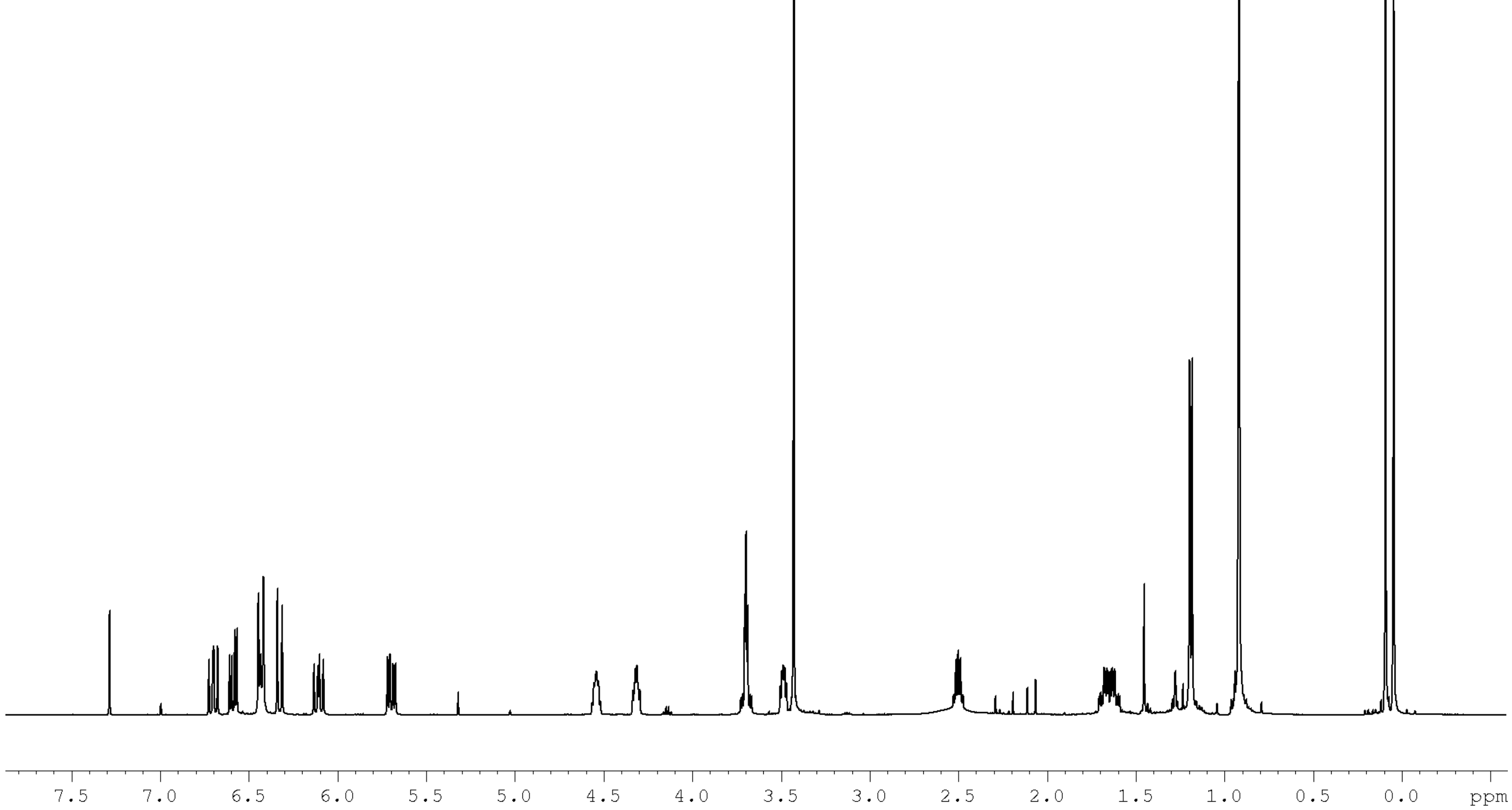

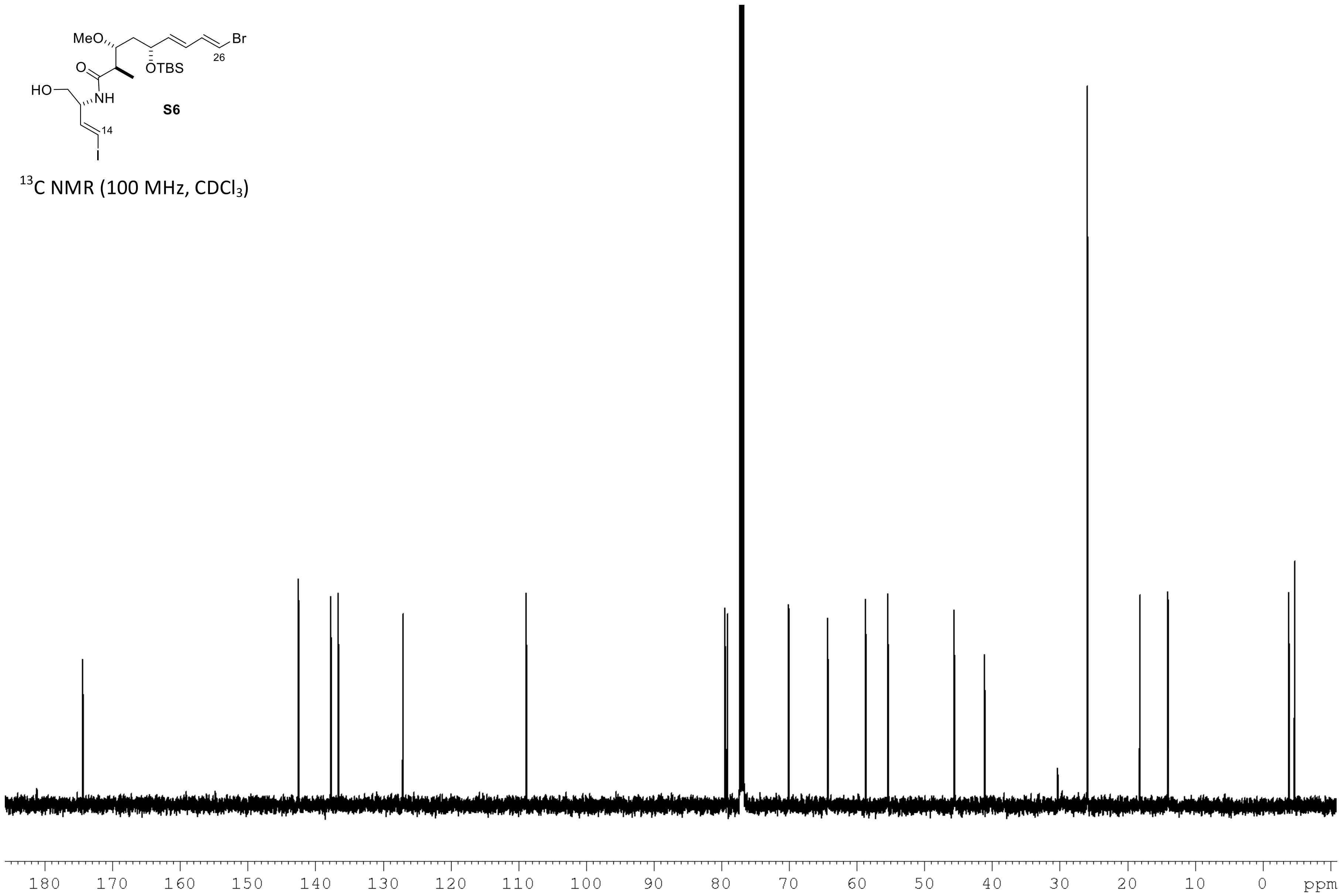

MeO
OTBS
Br
26
O
HO
NH
S6
14
I
^{13}C NMR (100 MHz, CDCl$_3$)
180 170 160 150 140 130 120 110 100 90 80 70 60 50 40 30 20 10 0 ppm

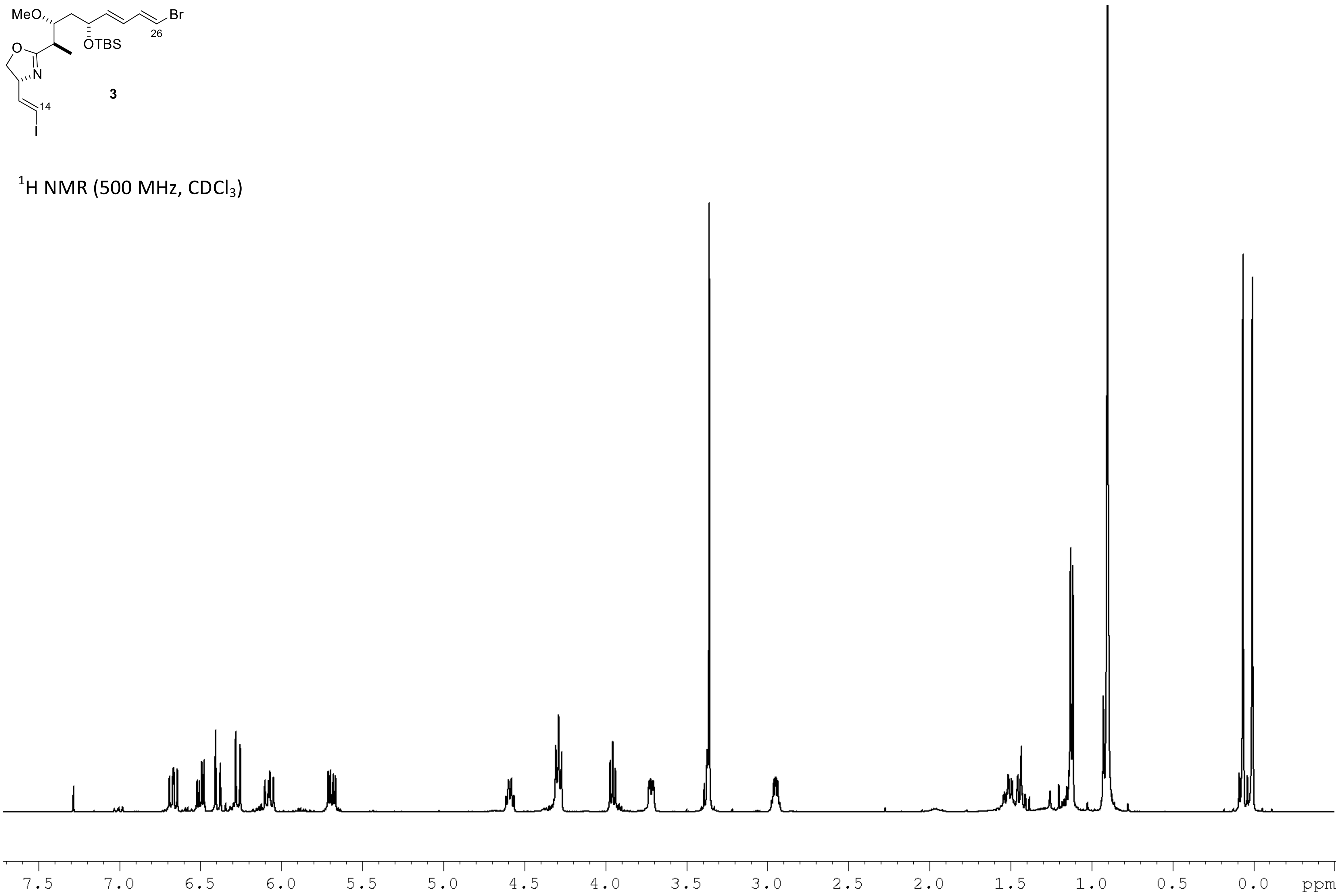
MeO
OTBS
Br
26
O
N
14
I
3
^{1}H NMR (500 MHz, CDCl$_3$)
7.5
7.0
6.5
6.0
5.5
5.0
4.5
4.0
3.5
3.0
2.5
2.0
1.5
1.0
0.5
0.0
ppm

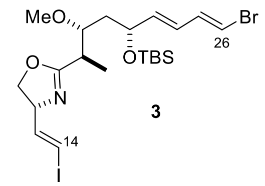

3

^{13}C NMR (125 MHz, $CDCl_3$)

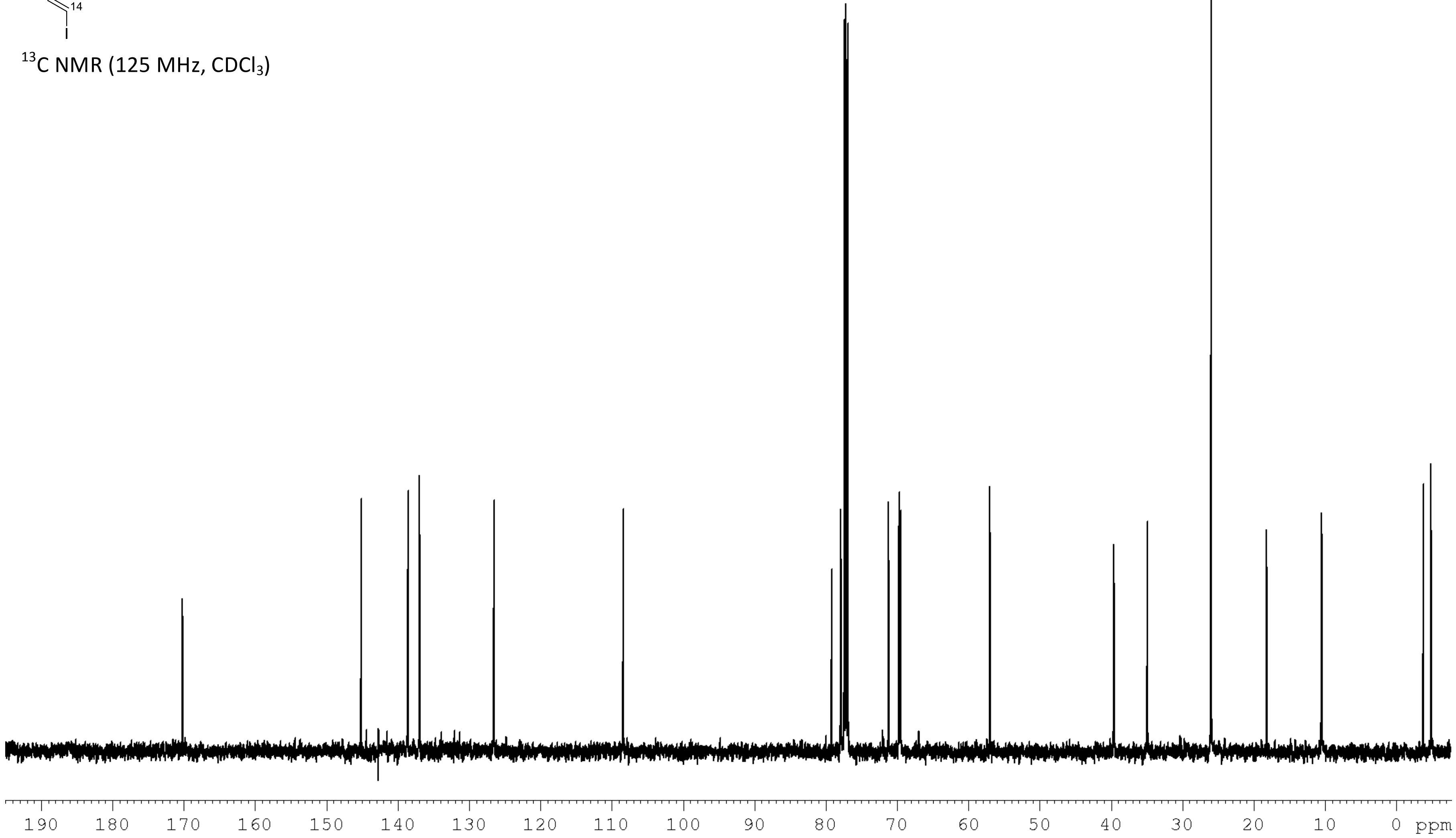

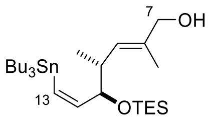

19

^{1}H NMR (500 MHz, $CDCl_3$)

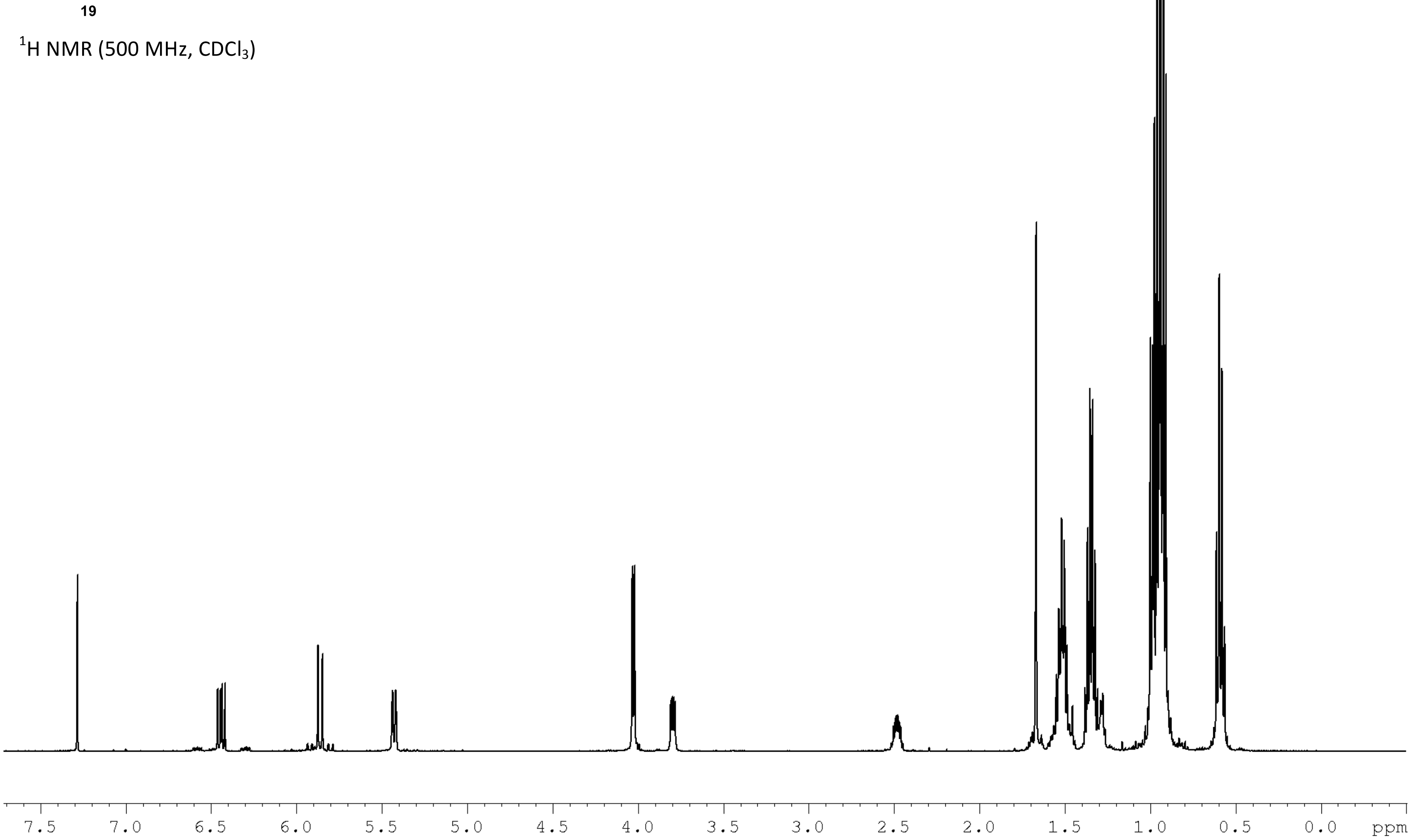

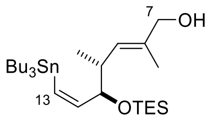

19

^{13}C NMR (125 MHz, $CDCl_3$)

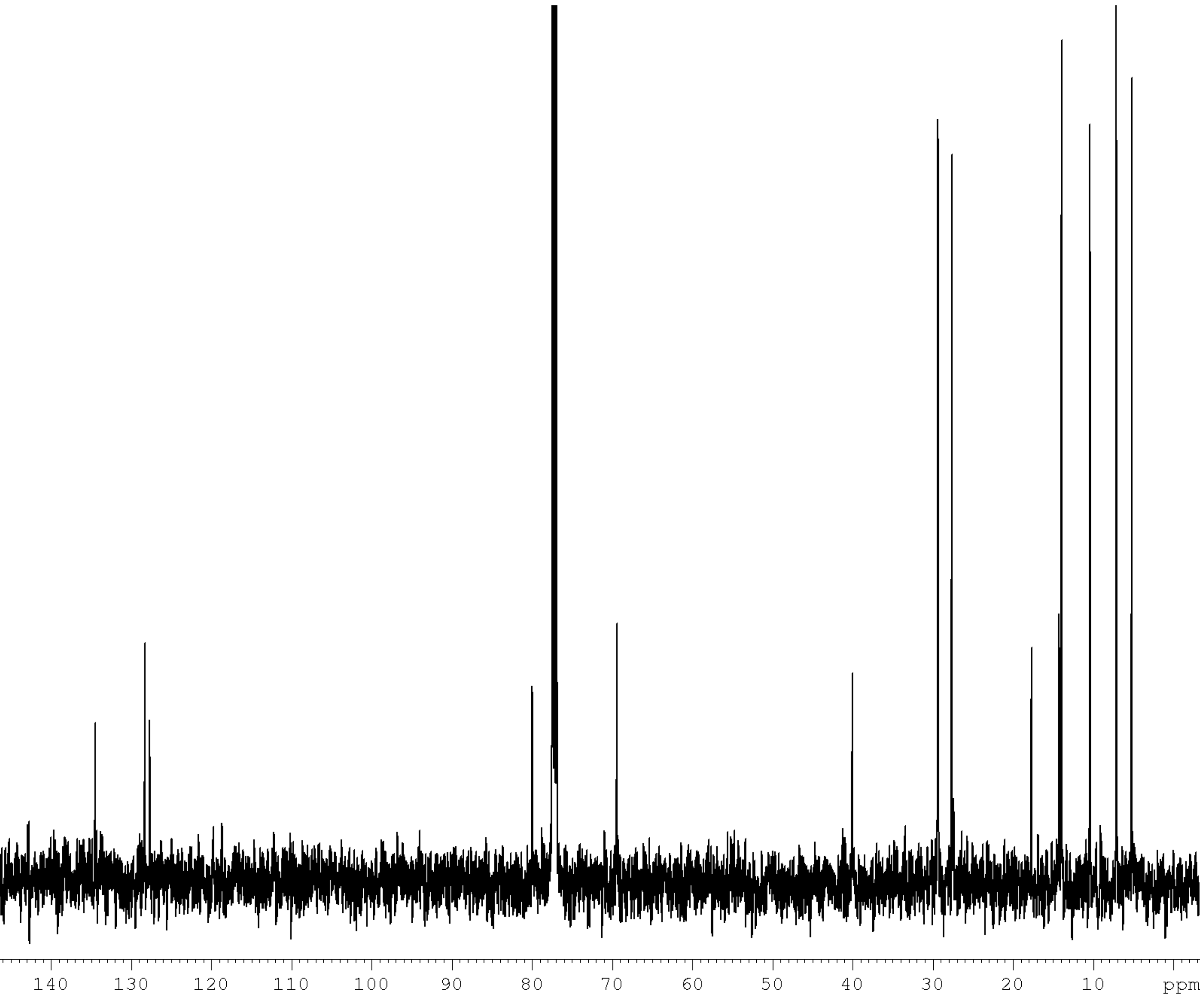

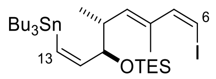

4

^{1}H NMR (500 MHz, $CDCl_3$)

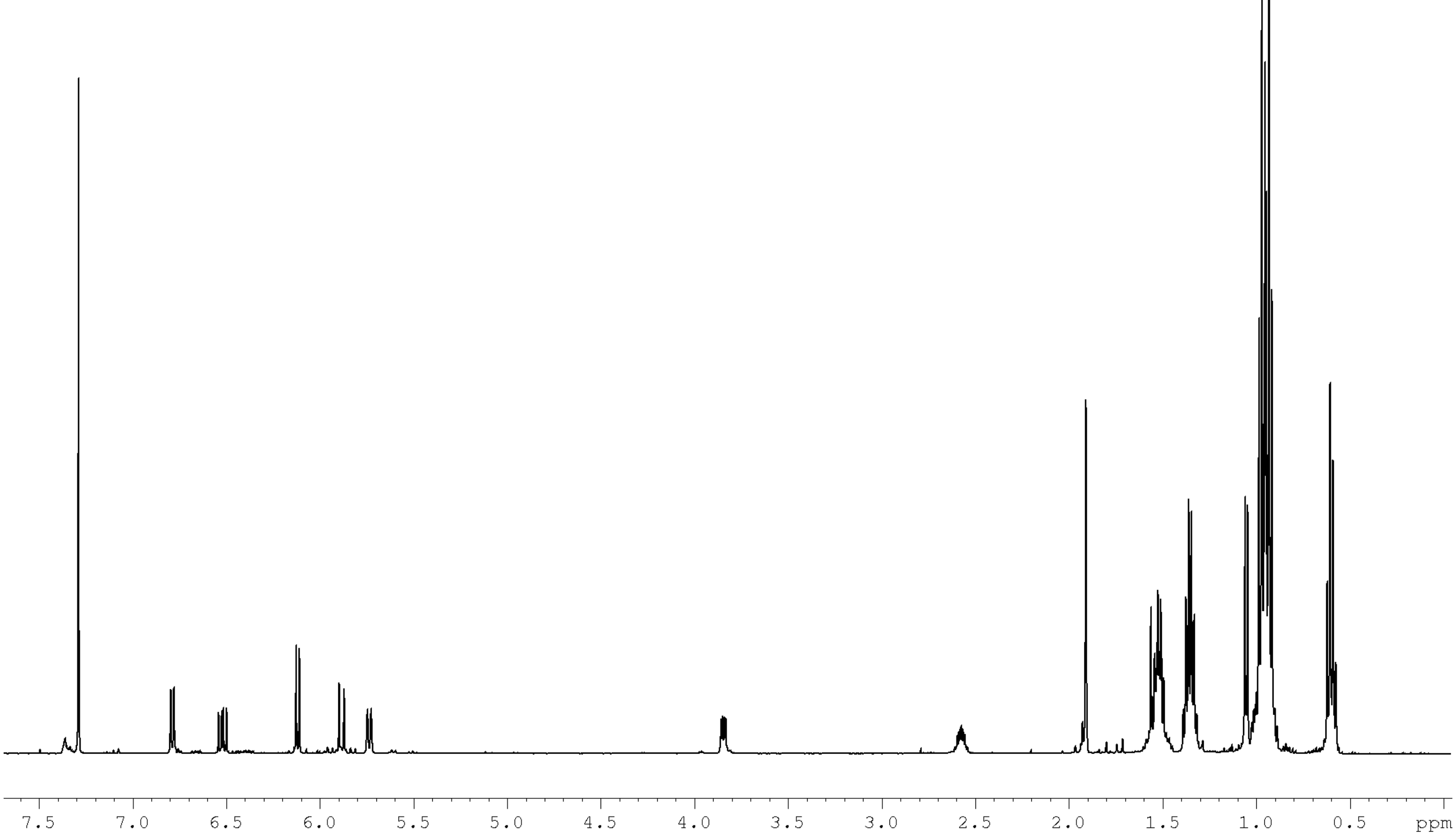

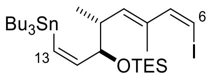

4

^{13}C NMR (125 MHz, $CDCl_3$)

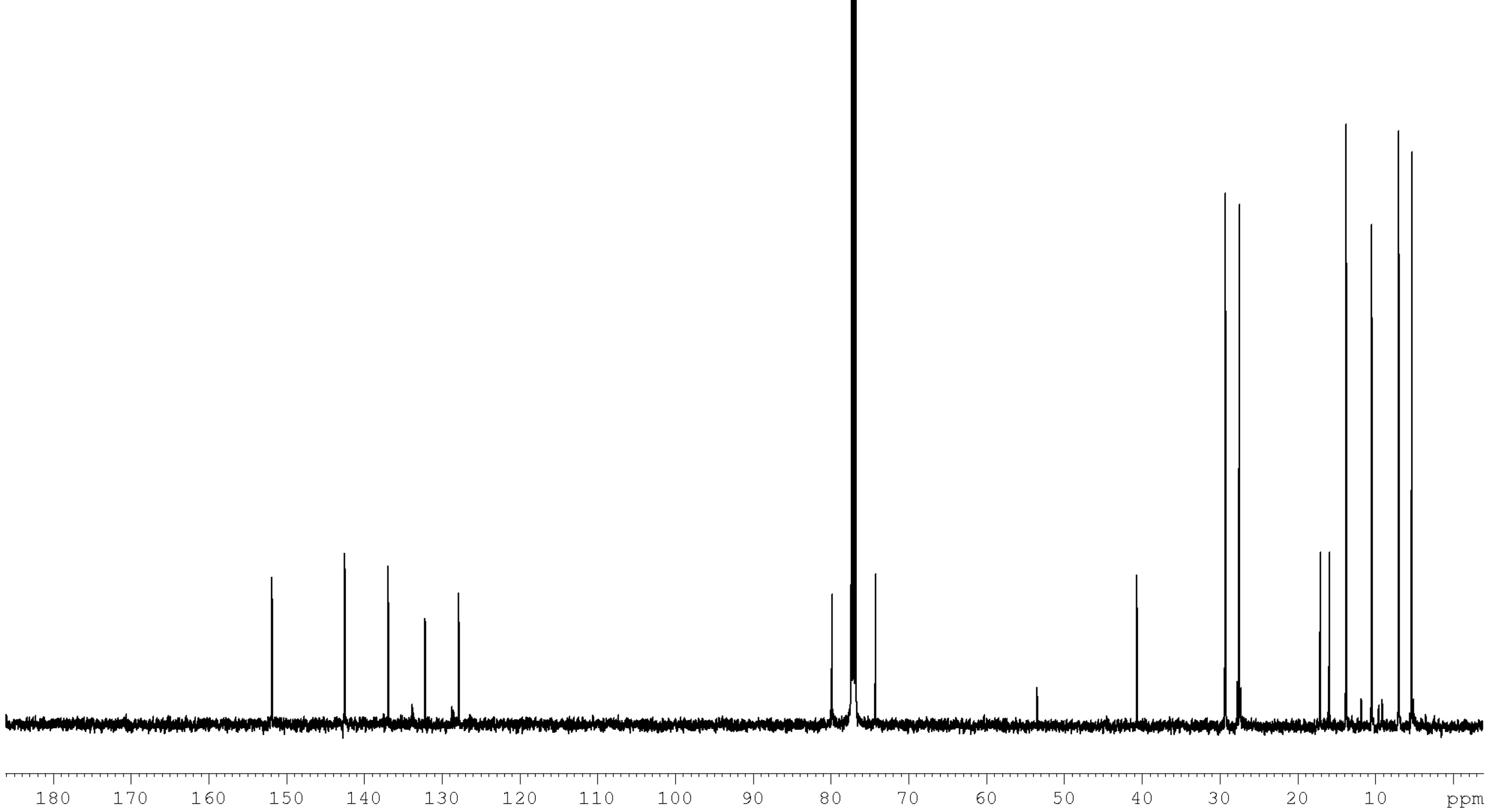

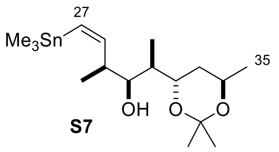

S7

^{1}H NMR (500 MHz, $CDCl_3$)

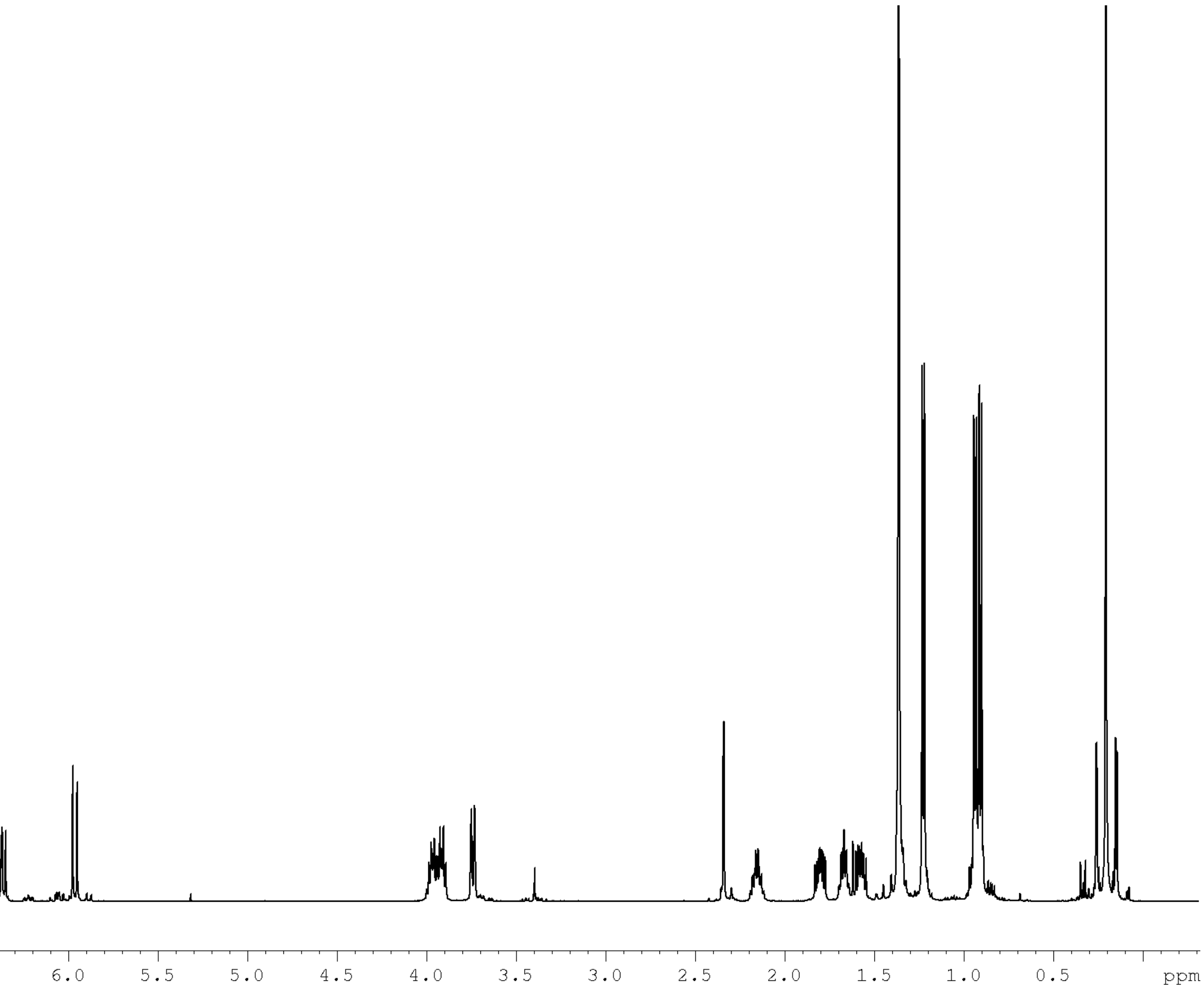

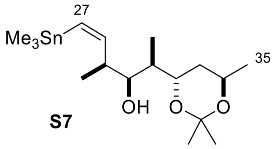

S7

^{13}C NMR (125 MHz, $CDCl_3$)

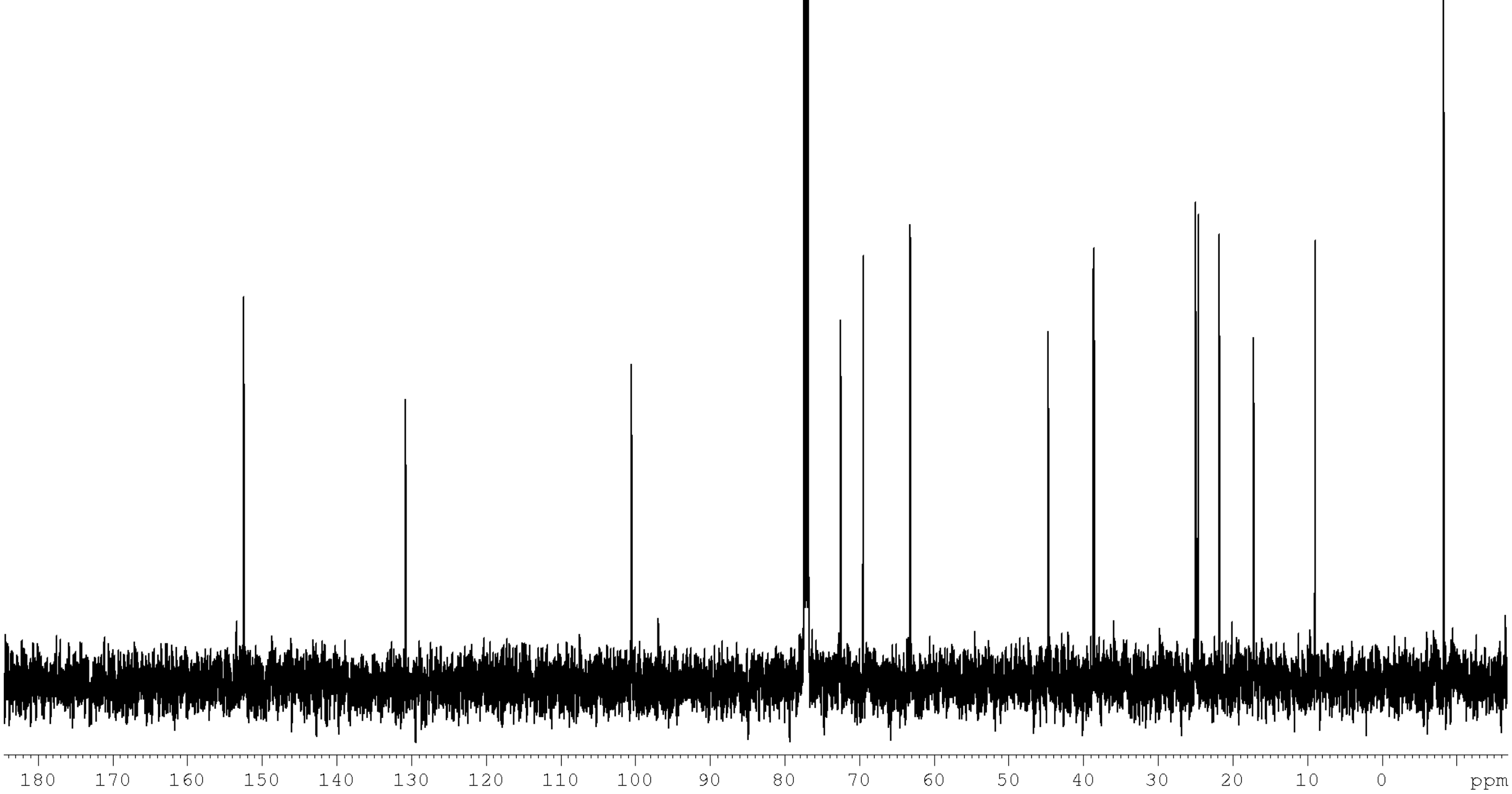

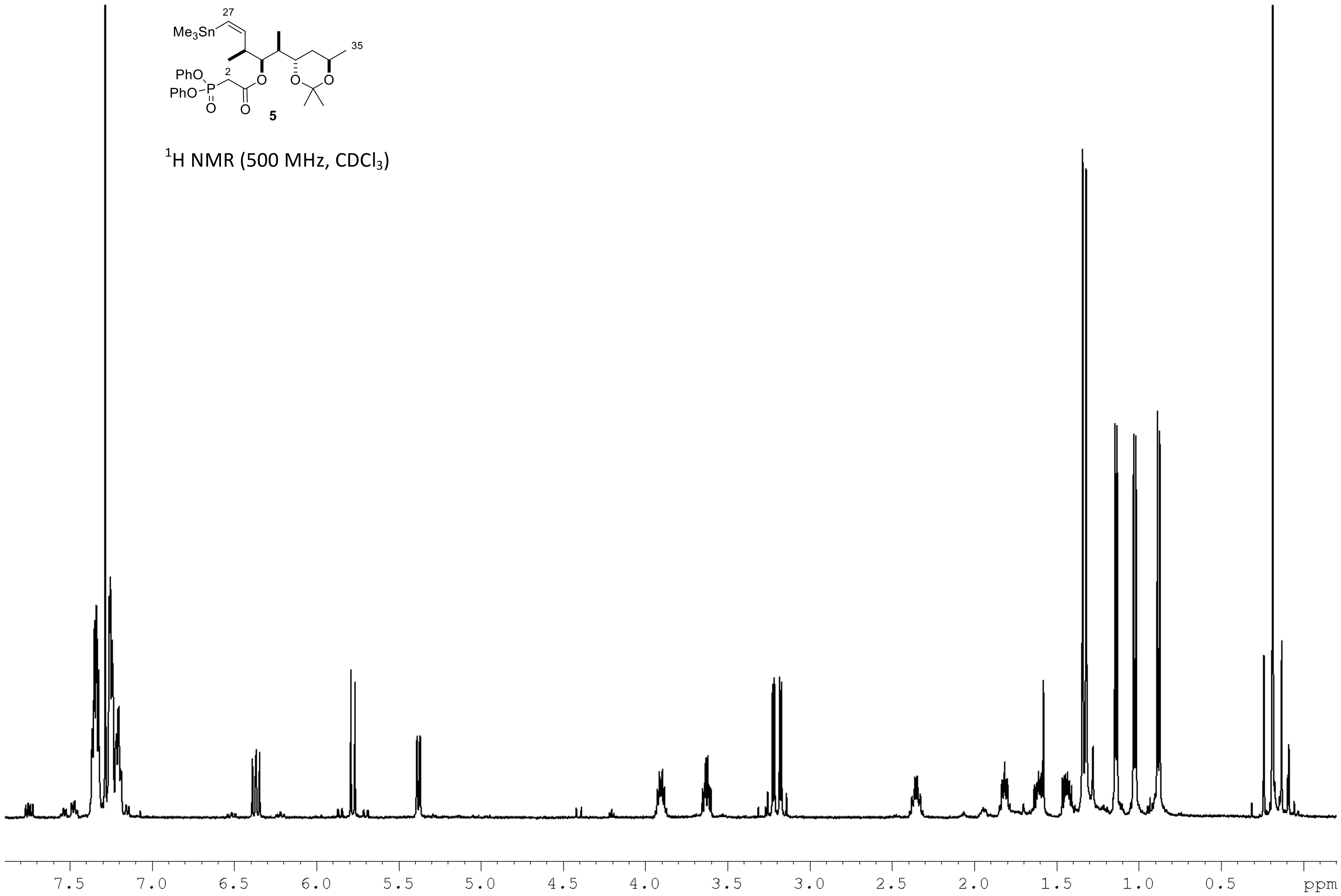
Me3Sn
27
2
35
PhO
PhO
P
O
O
O
O
O
5
1H NMR (500 MHz, CDCl3)
7.5 7.0 6.5 6.0 5.5 5.0 4.5 4.0 3.5 3.0 2.5 2.0 1.5 1.0 0.5 ppm

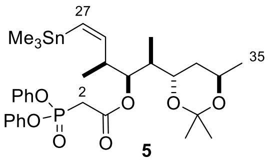

5

^{13}C NMR (125 MHz, $CDCl_3$)

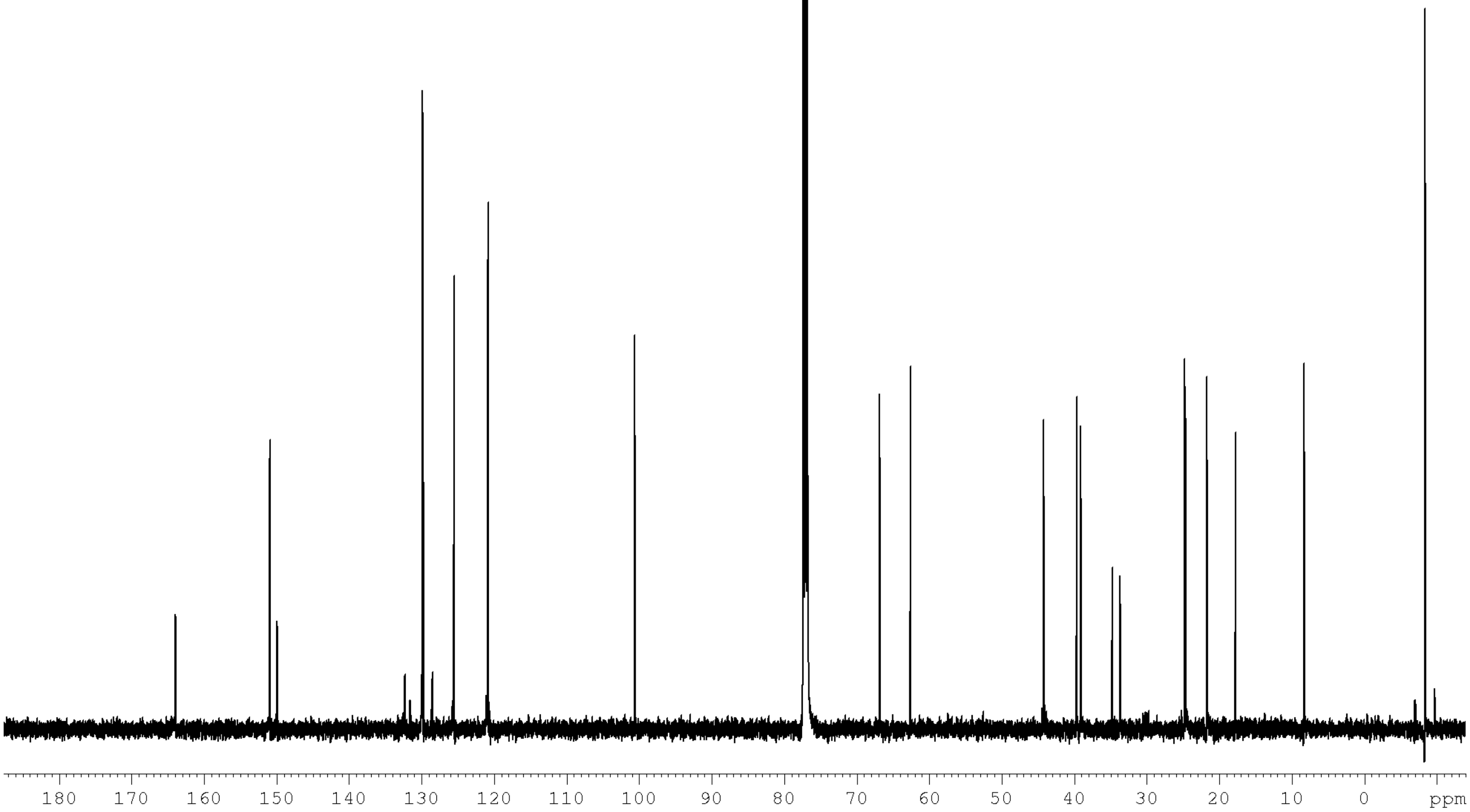

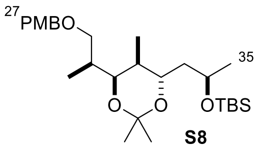

S8

^{1}H NMR (500 MHz, $CDCl_3$)

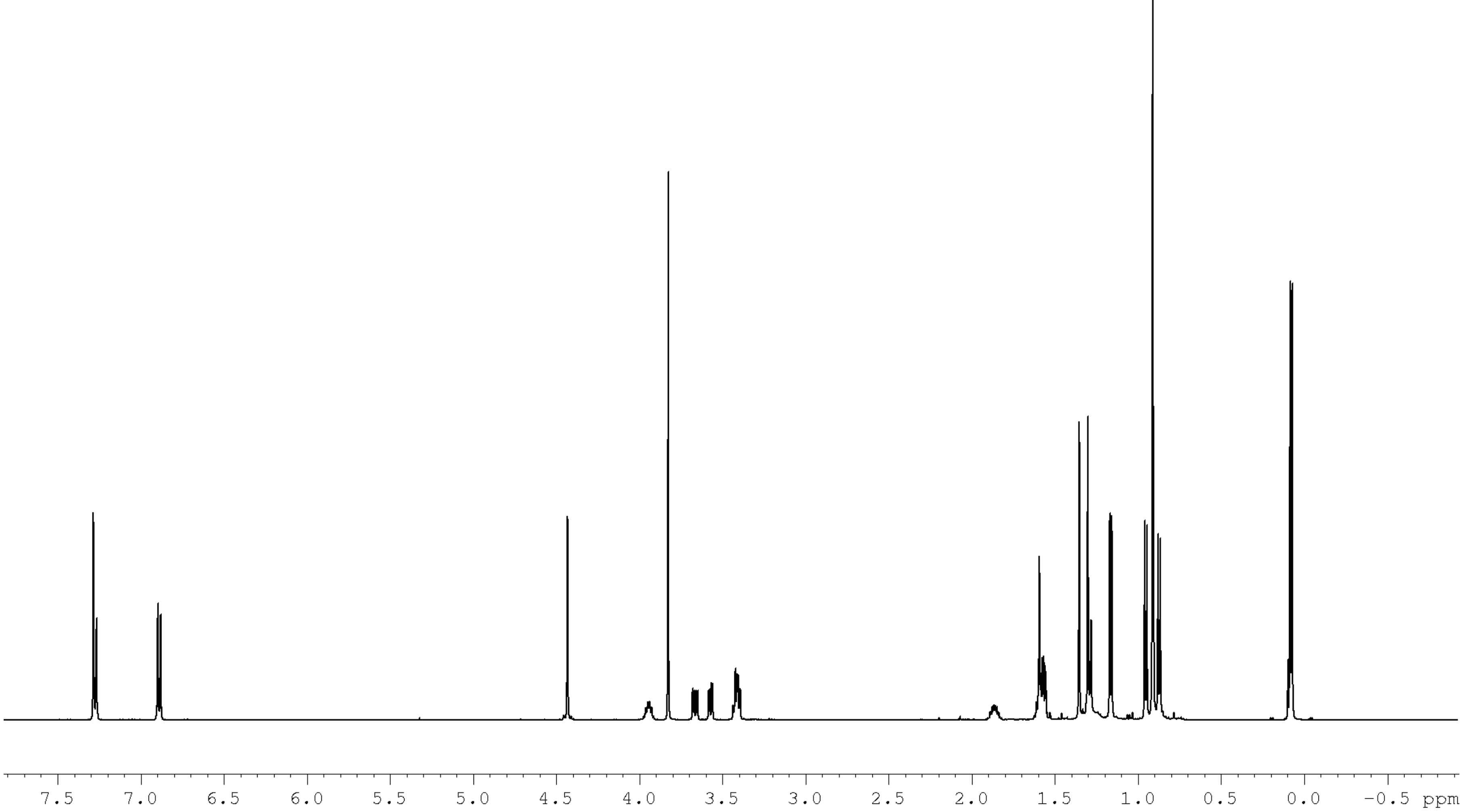

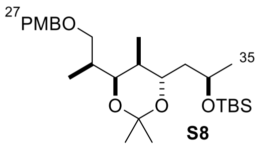

S8

^{13}C NMR (125 MHz, $CDCl_3$)

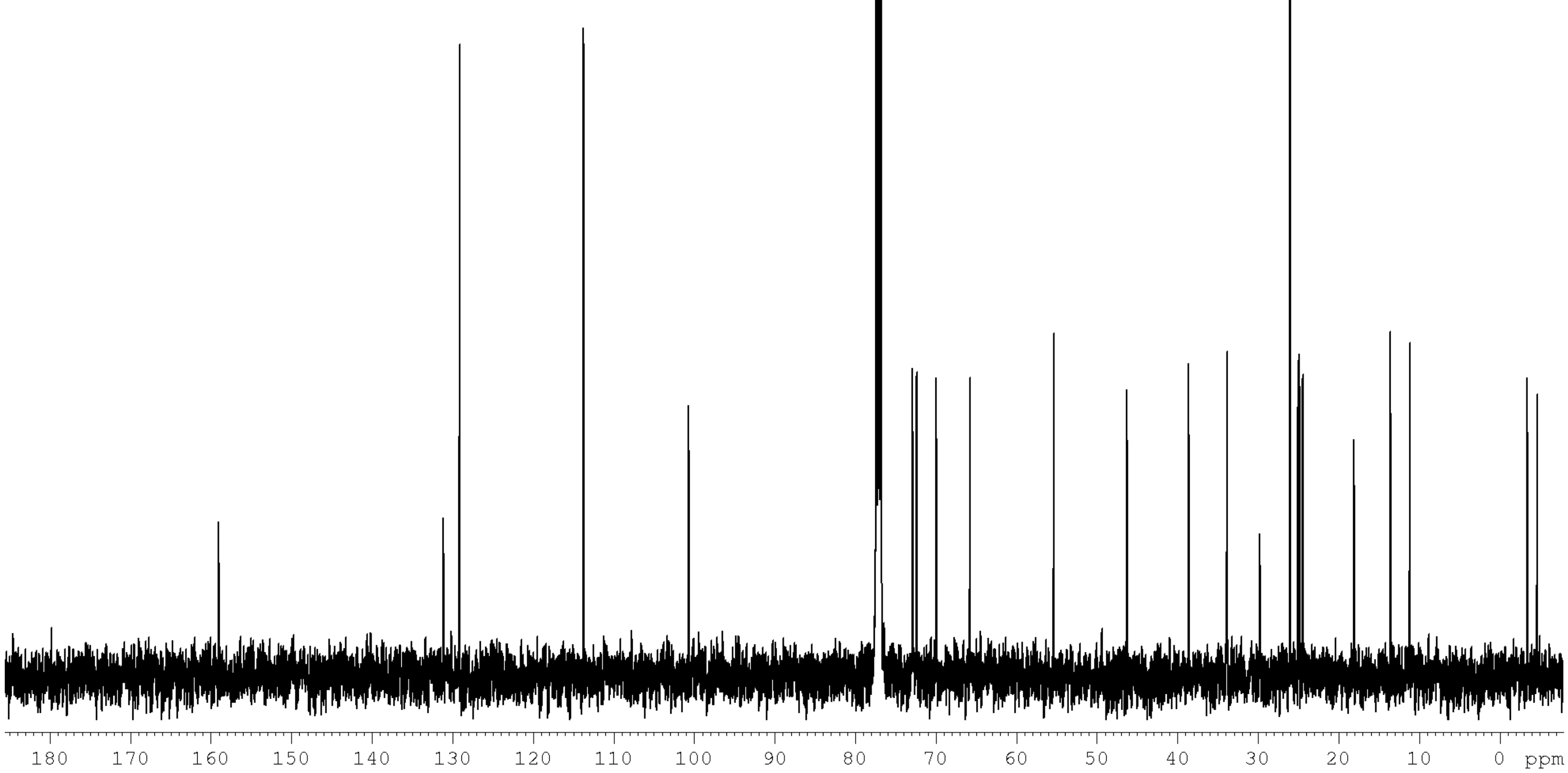

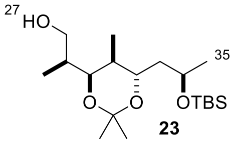

23

^{1}H NMR (500 MHz, $CDCl_3$)

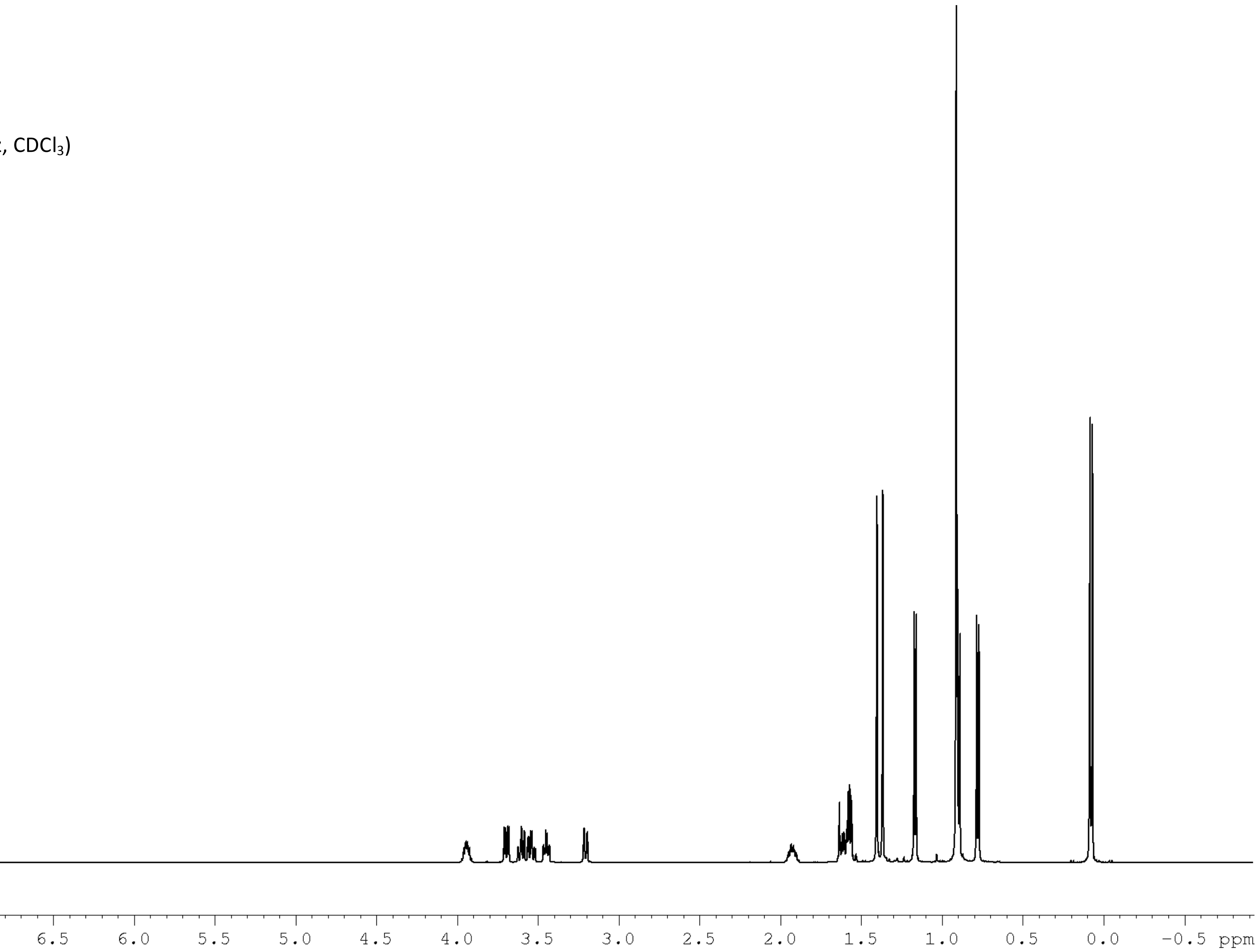

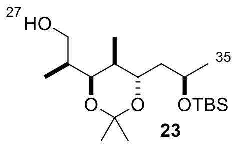

23

^{13}C NMR (125 MHz, $CDCl_3$)

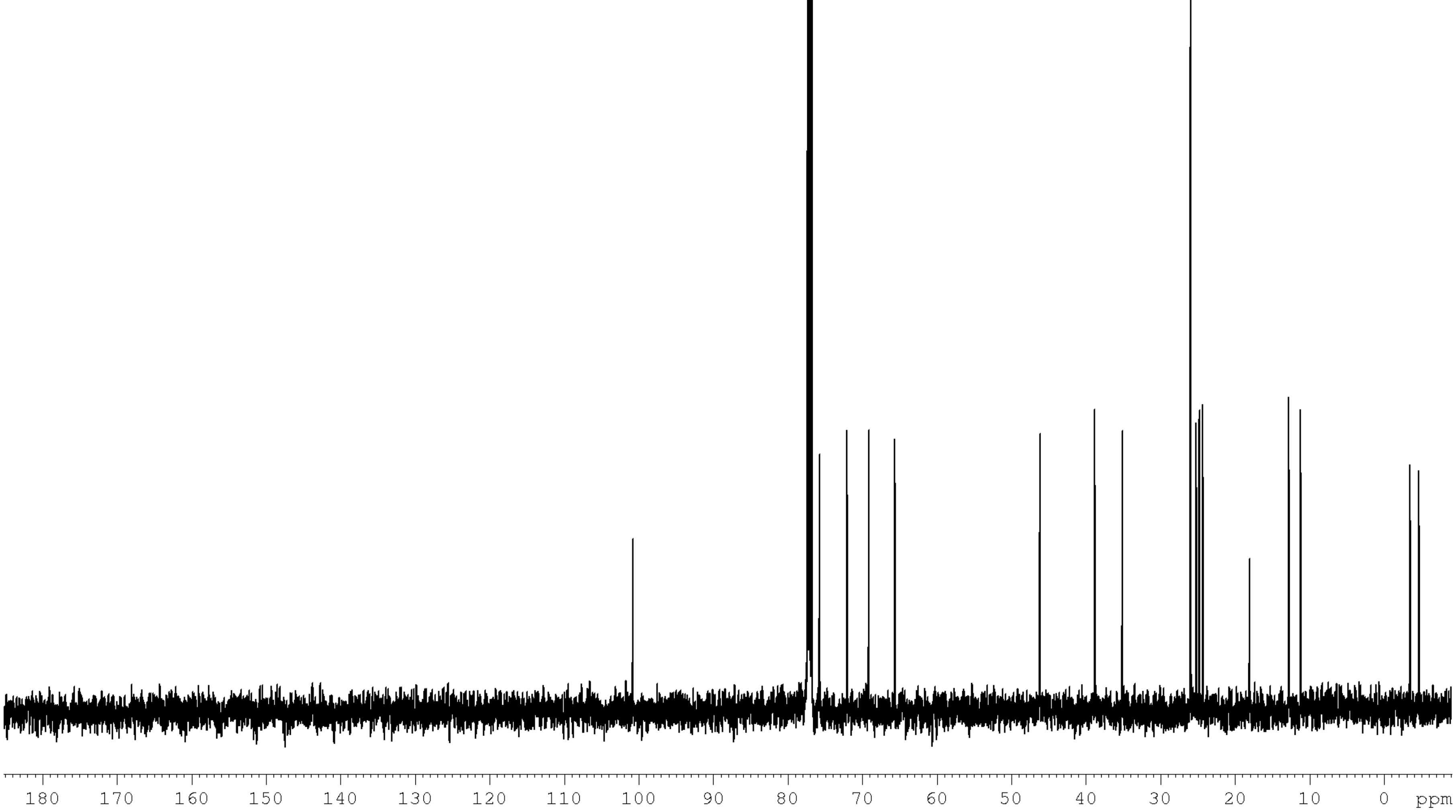

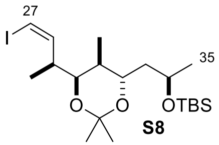

^{1}H NMR (500 MHz, $CDCl_3$)

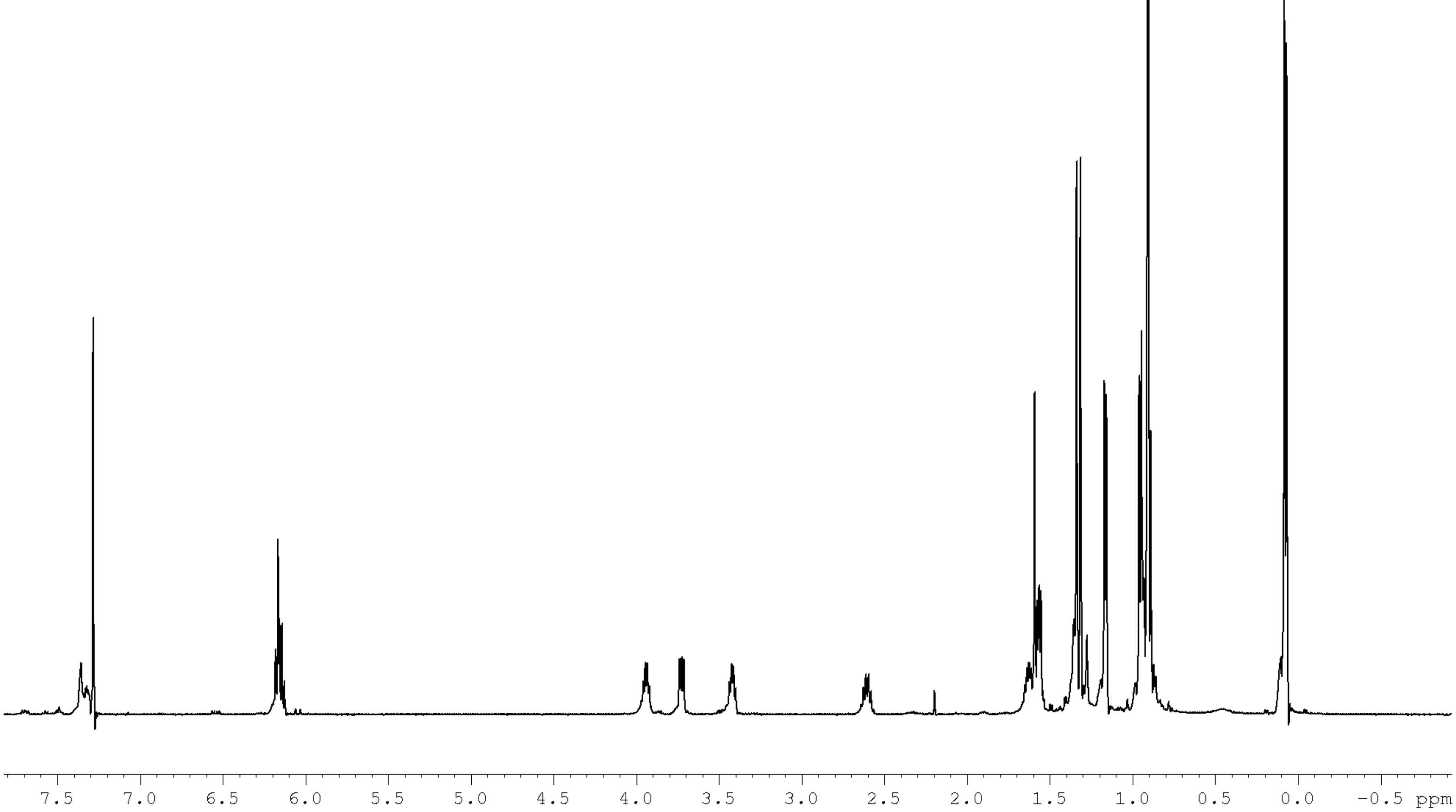

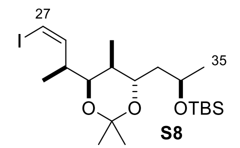

^{13}C NMR (125 MHz, $CDCl_3$)

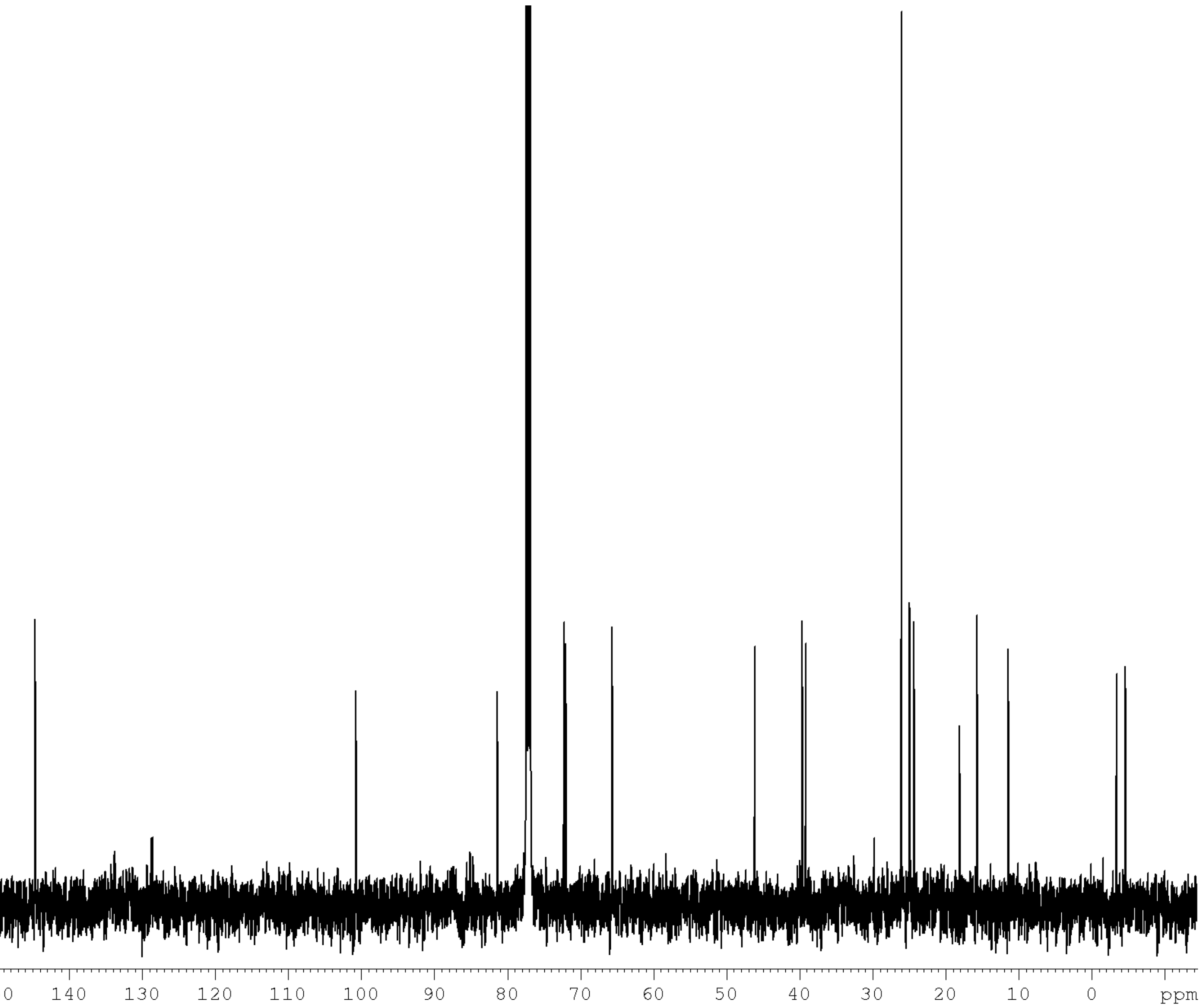

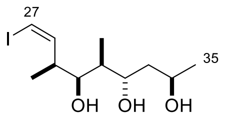

24

^{1}H NMR (500 MHz, $CDCl_3$)

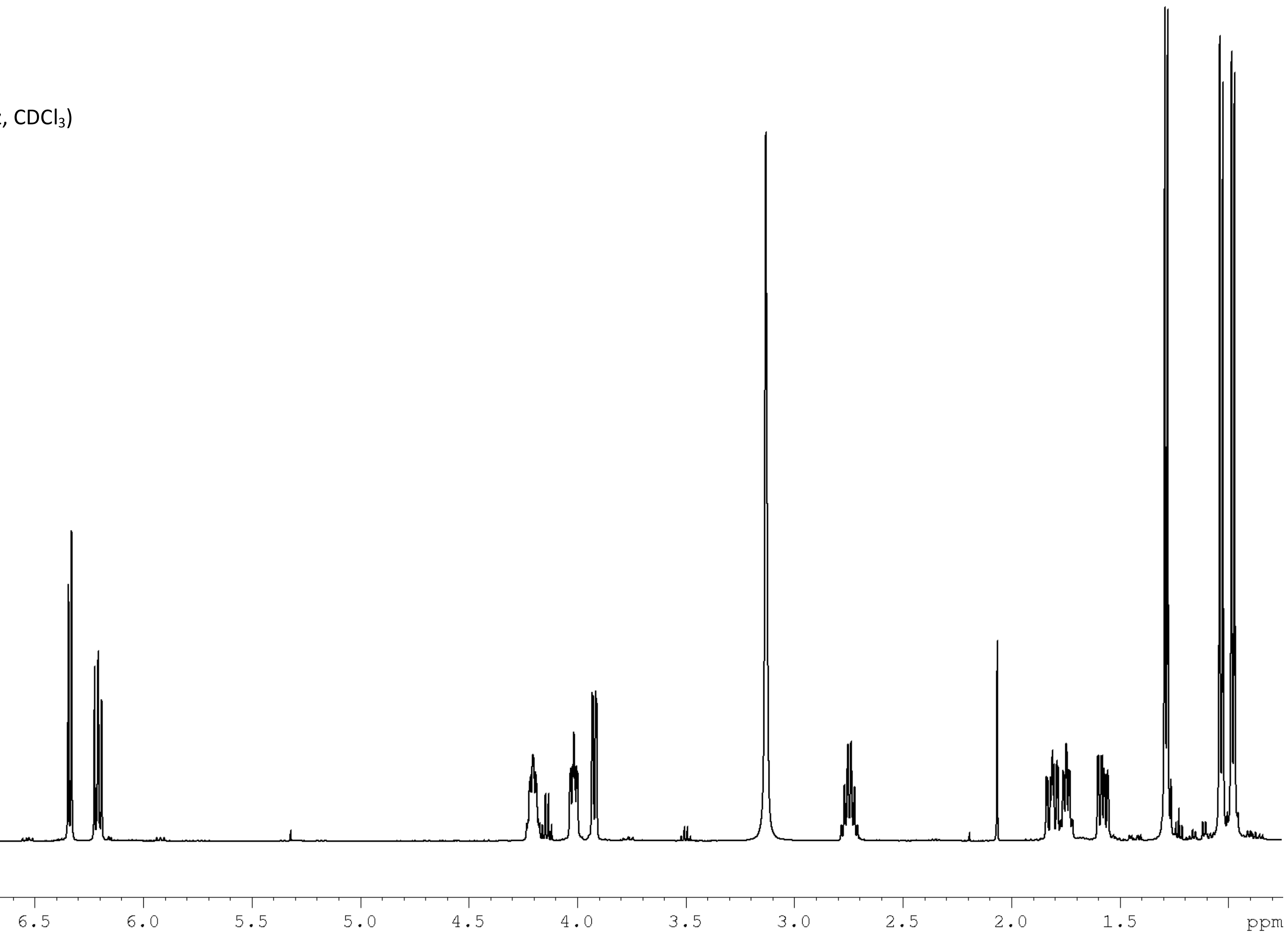

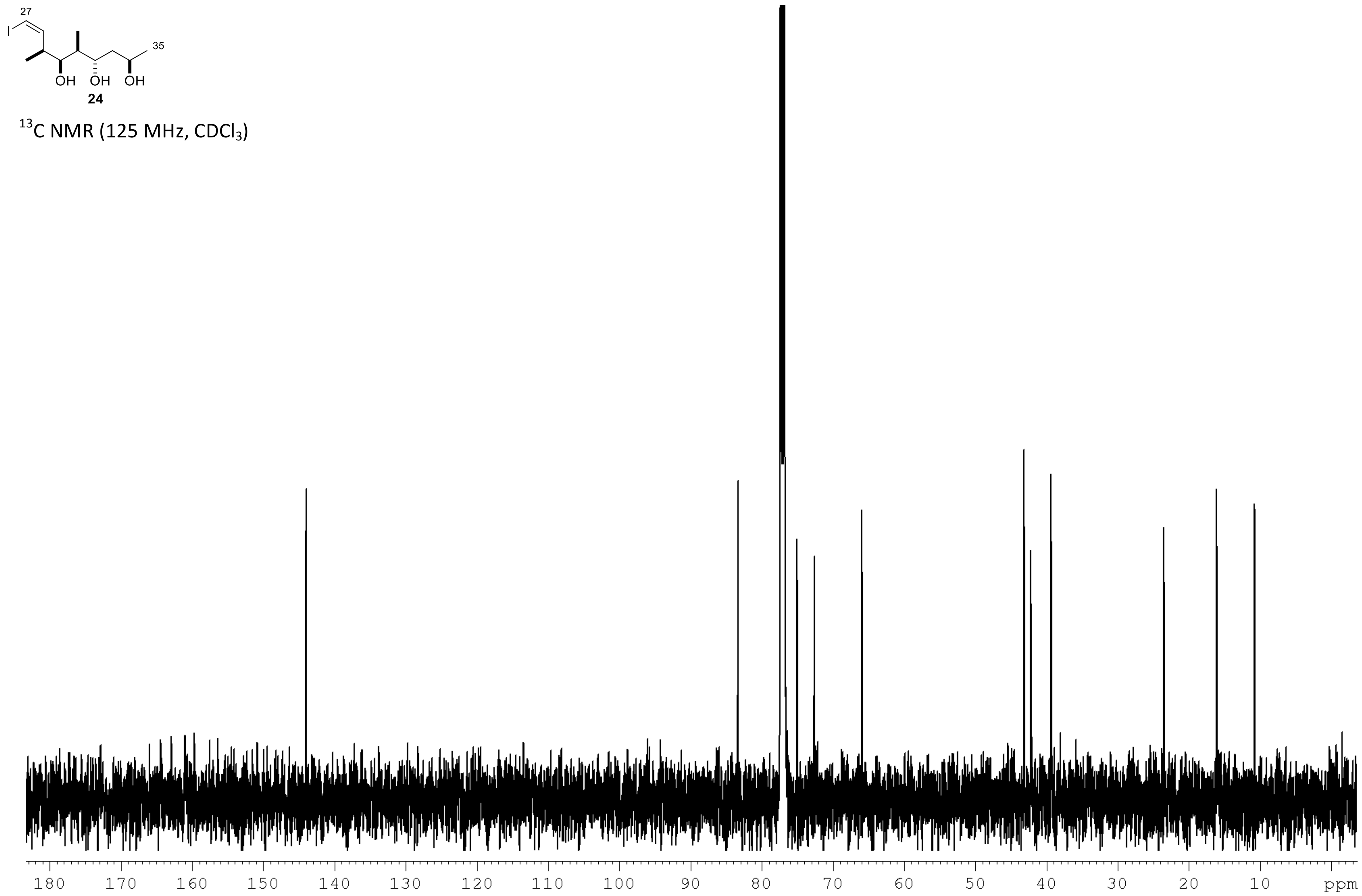
27
I
35
OH OH OH
24
13C NMR (125 MHz, CDCl3)
180 170 160 150 140 130 120 110 100 90 80 70 60 50 40 30 20 10 ppm

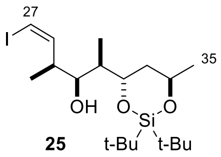

^{1}H NMR (500 MHz, $CDCl_3$)

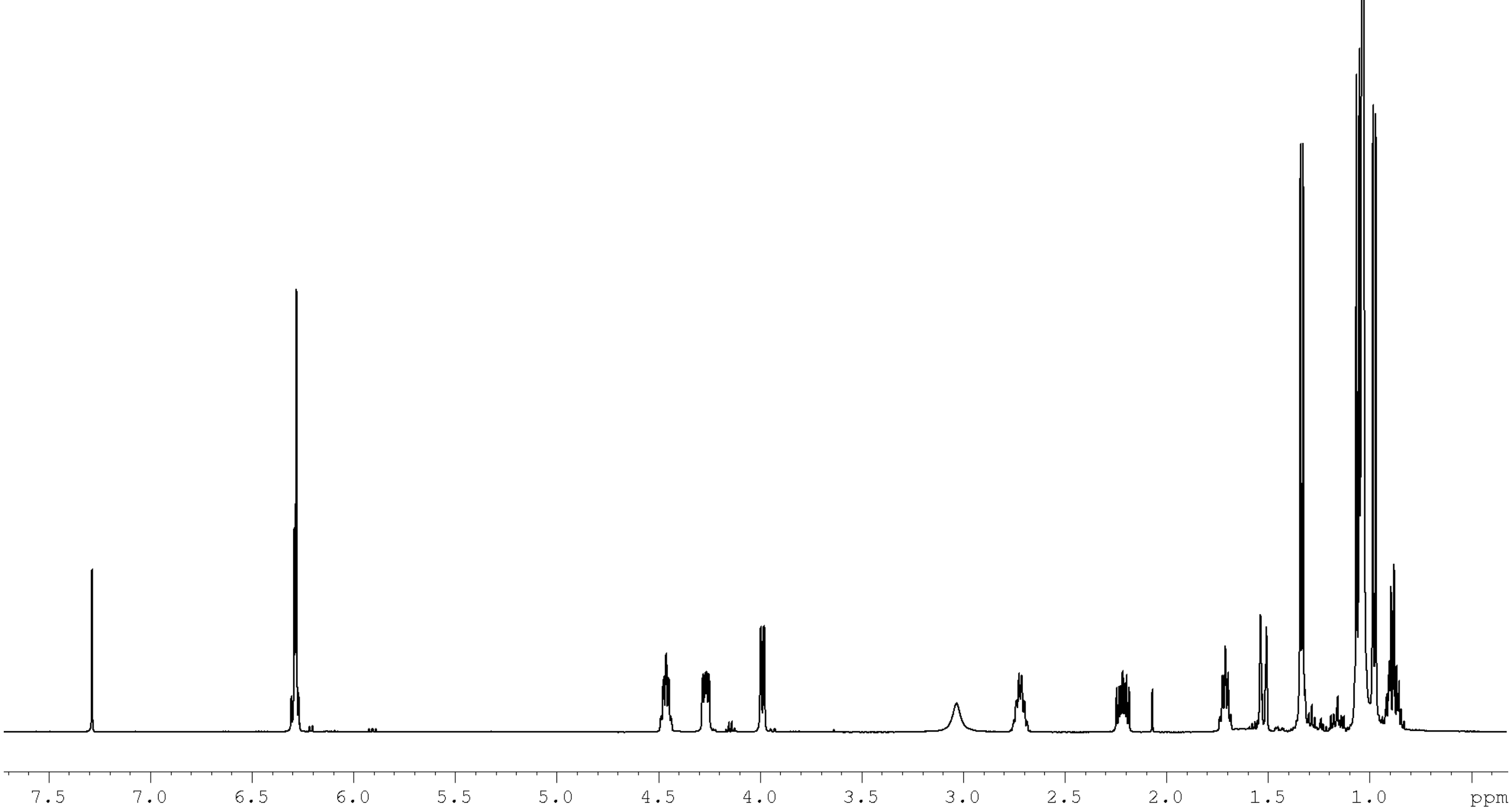

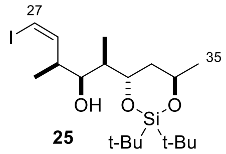

25

^{13}C NMR (125 MHz, $CDCl_3$)

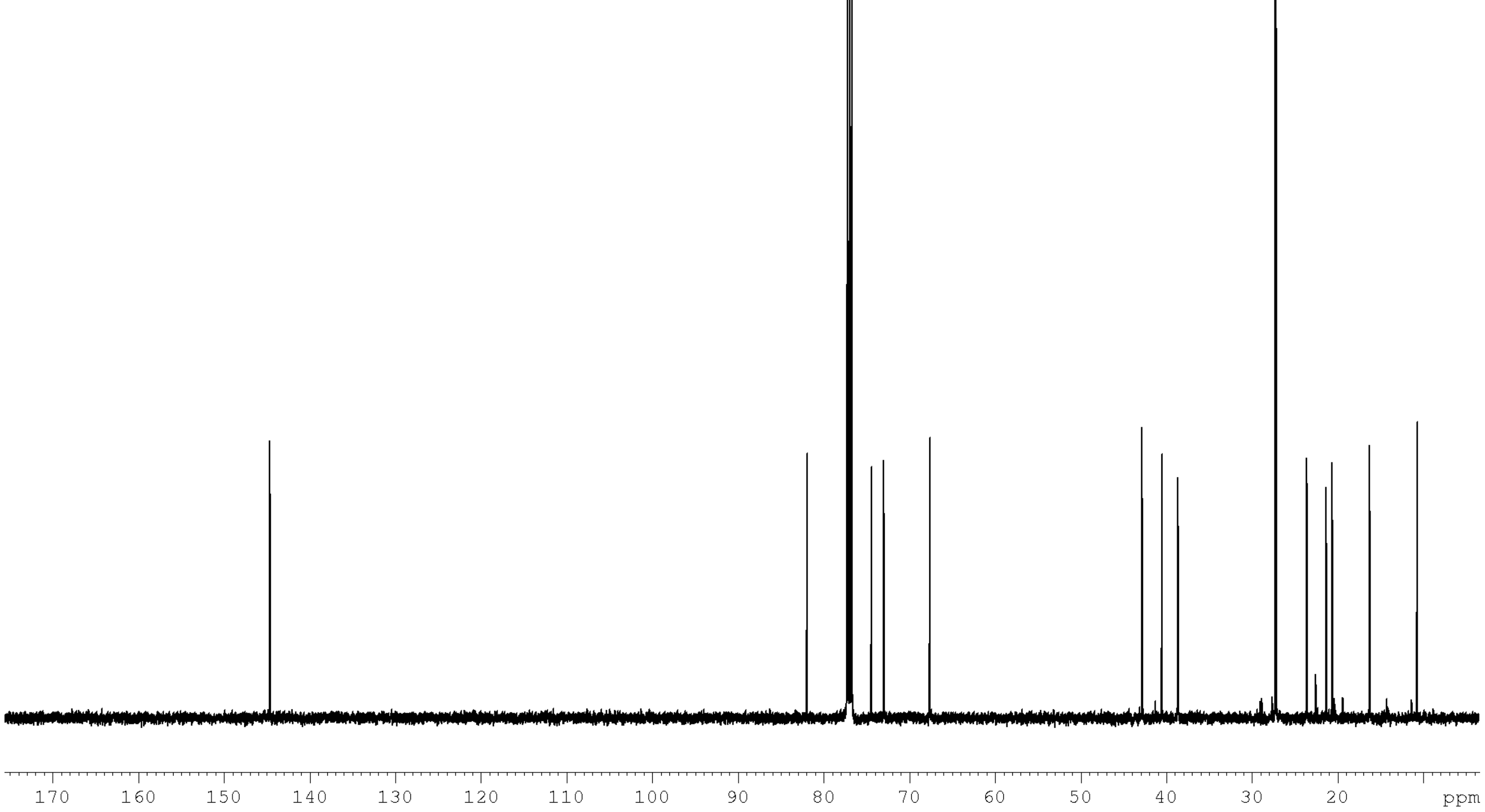

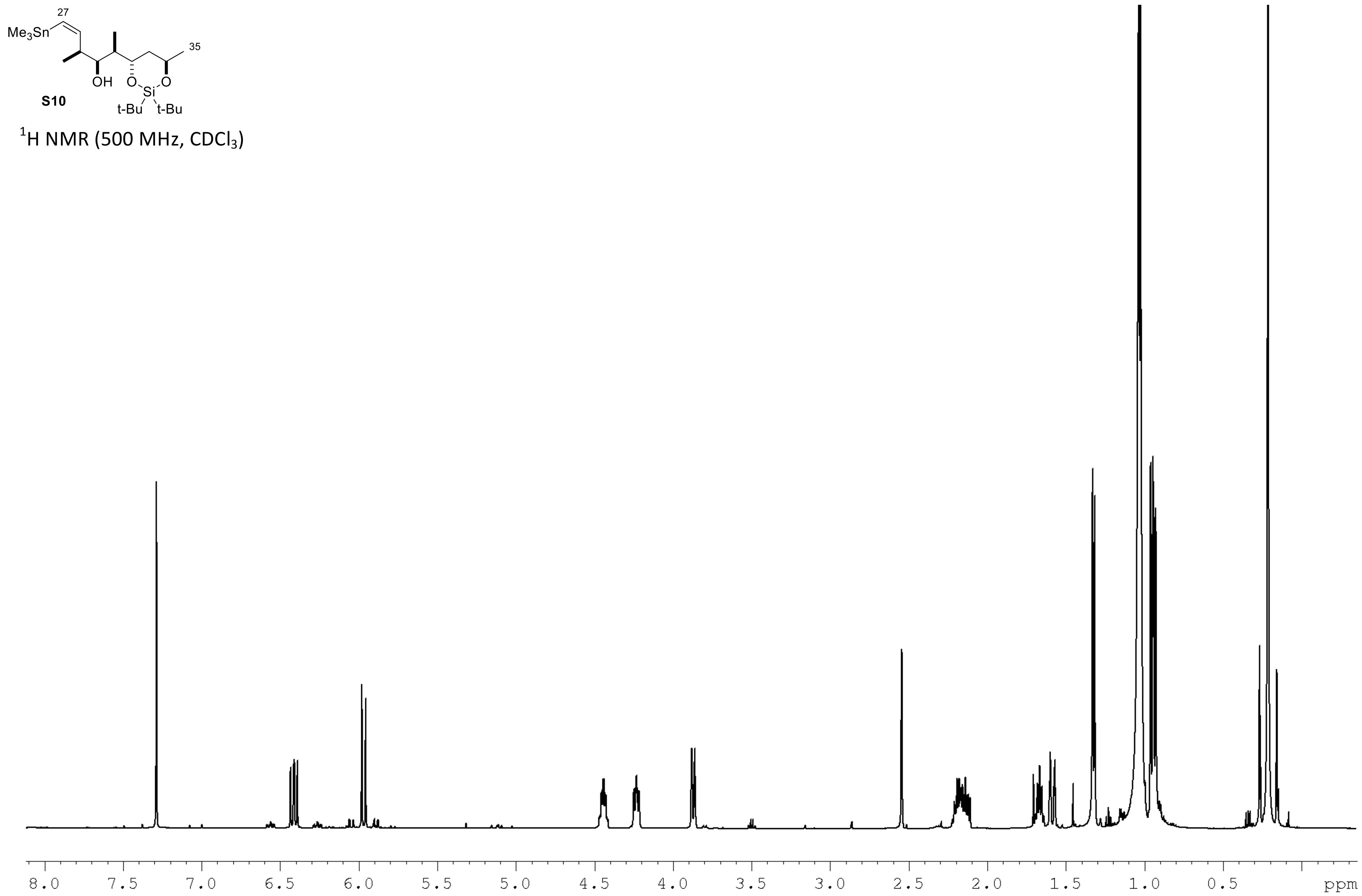
Me3Sn
27
35
OH
O
Si
O
t-Bu
t-Bu
S10
1H NMR (500 MHz, CDCl3)
8.0
7.5
7.0
6.5
6.0
5.5
5.0
4.5
4.0
3.5
3.0
2.5
2.0
1.5
1.0
0.5
ppm

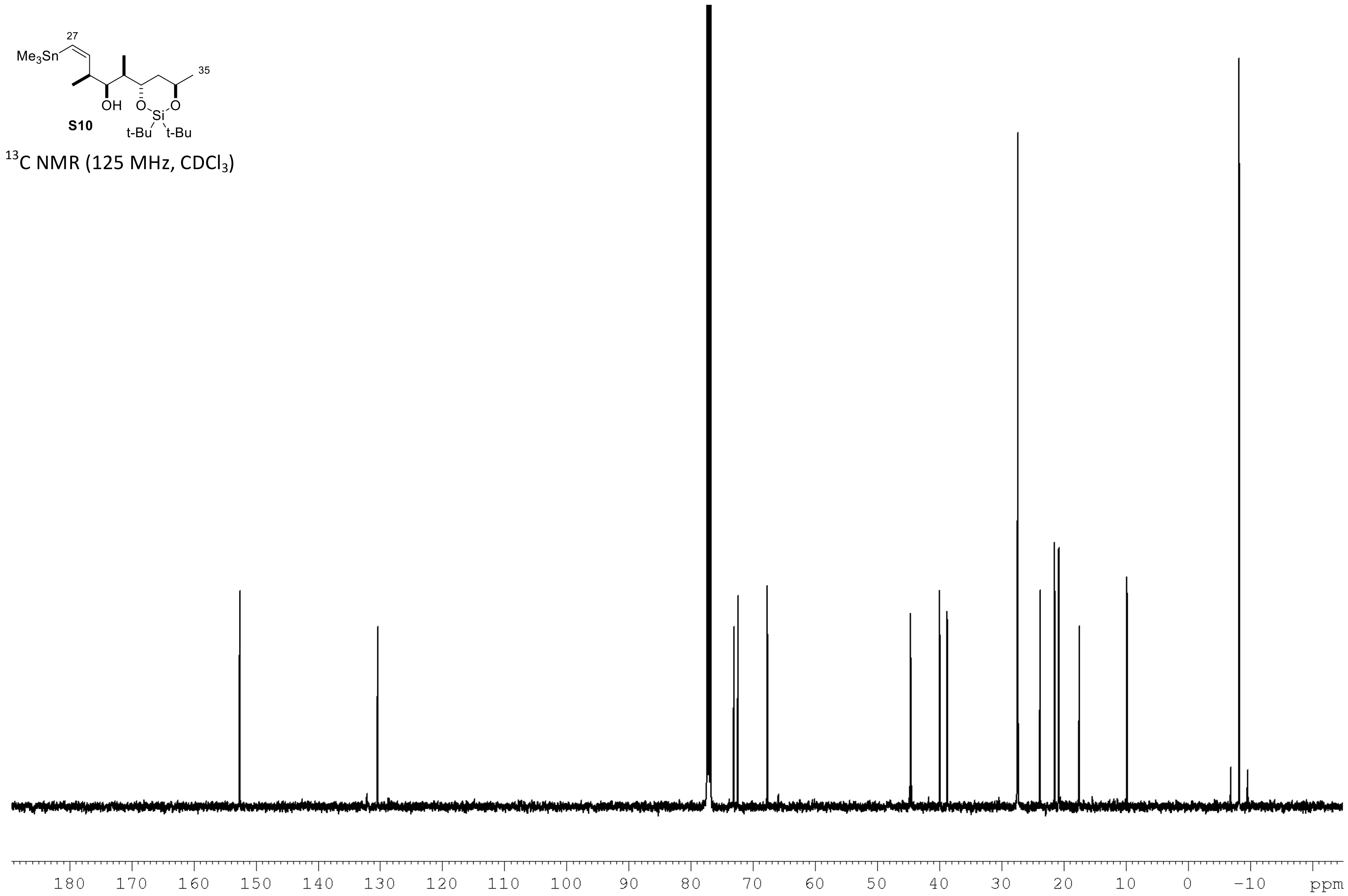
27
Me3Sn
35
OH
O
Si
O
t-Bu
t-Bu
S10
13C NMR (125 MHz, CDCl3)
180 170 160 150 140 130 120 110 100 90 80 70 60 50 40 30 20 10 0 -10 ppm

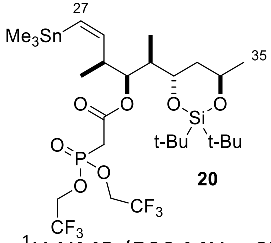

20

^{1}H NMR (500 MHz, $CDCl_3$)

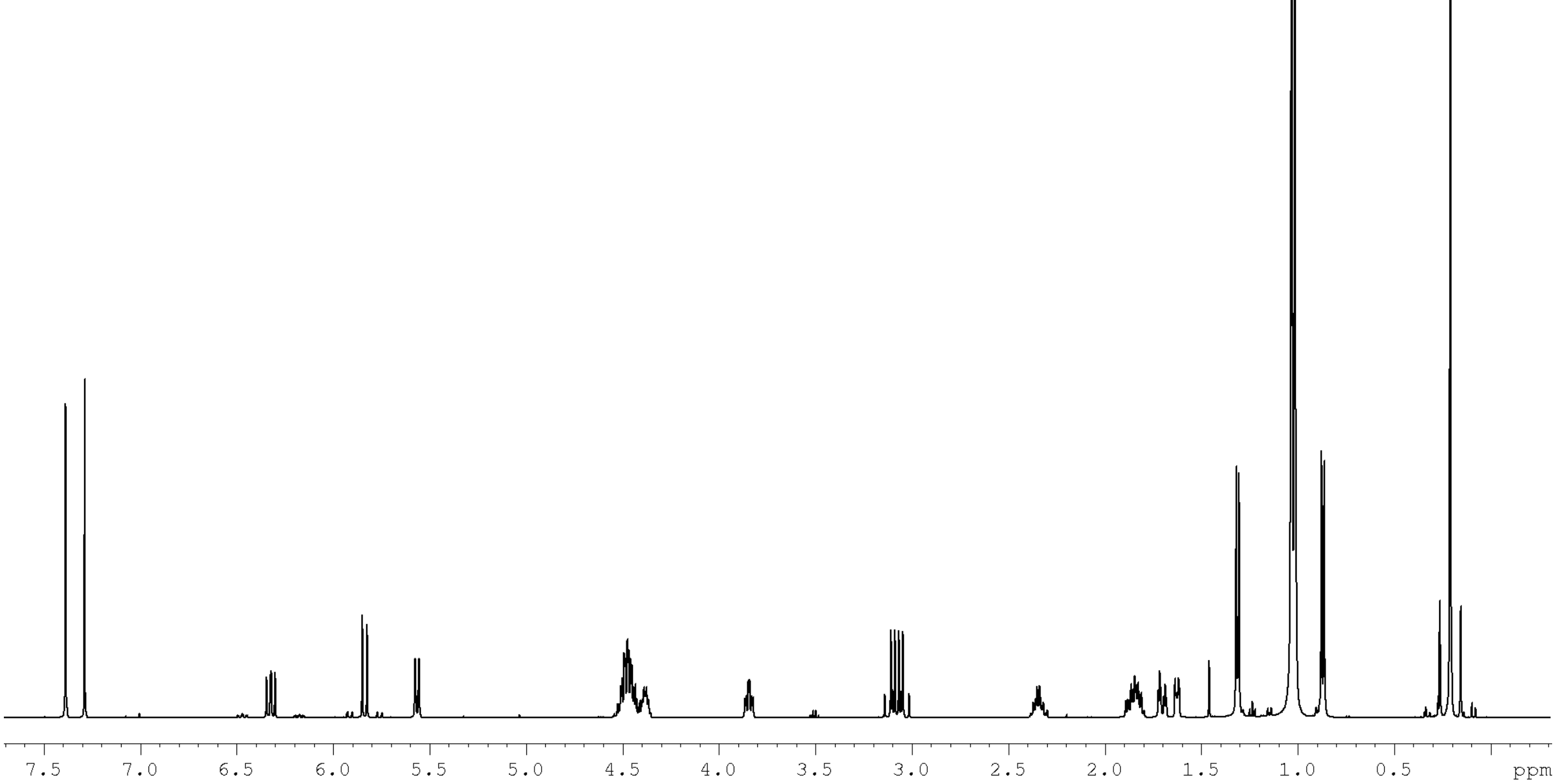

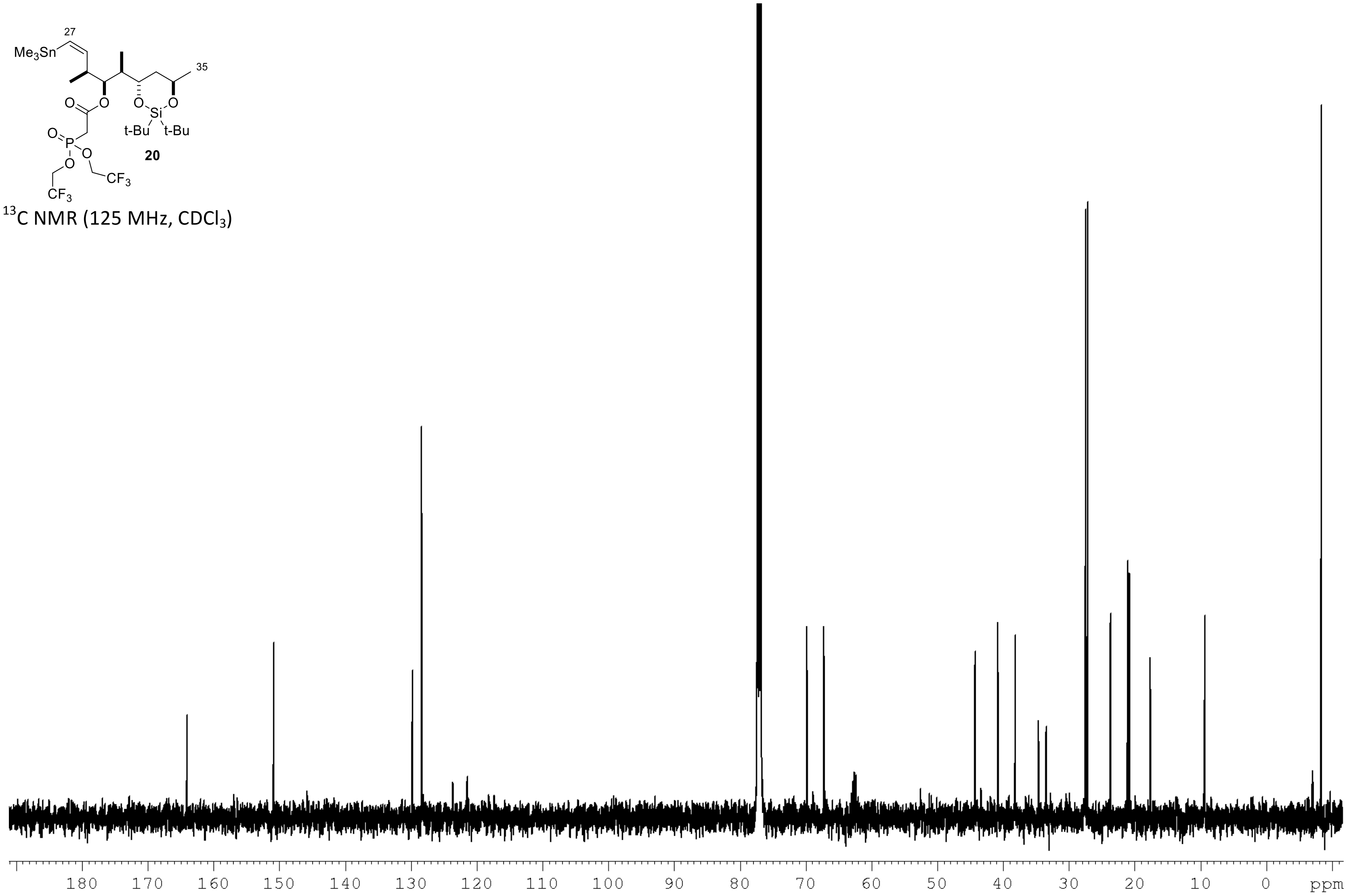

20

^{13}C NMR (125 MHz, $CDCl_3$)

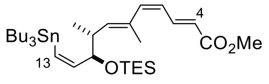

16

^{1}H NMR (500 MHz, $CDCl_3$)

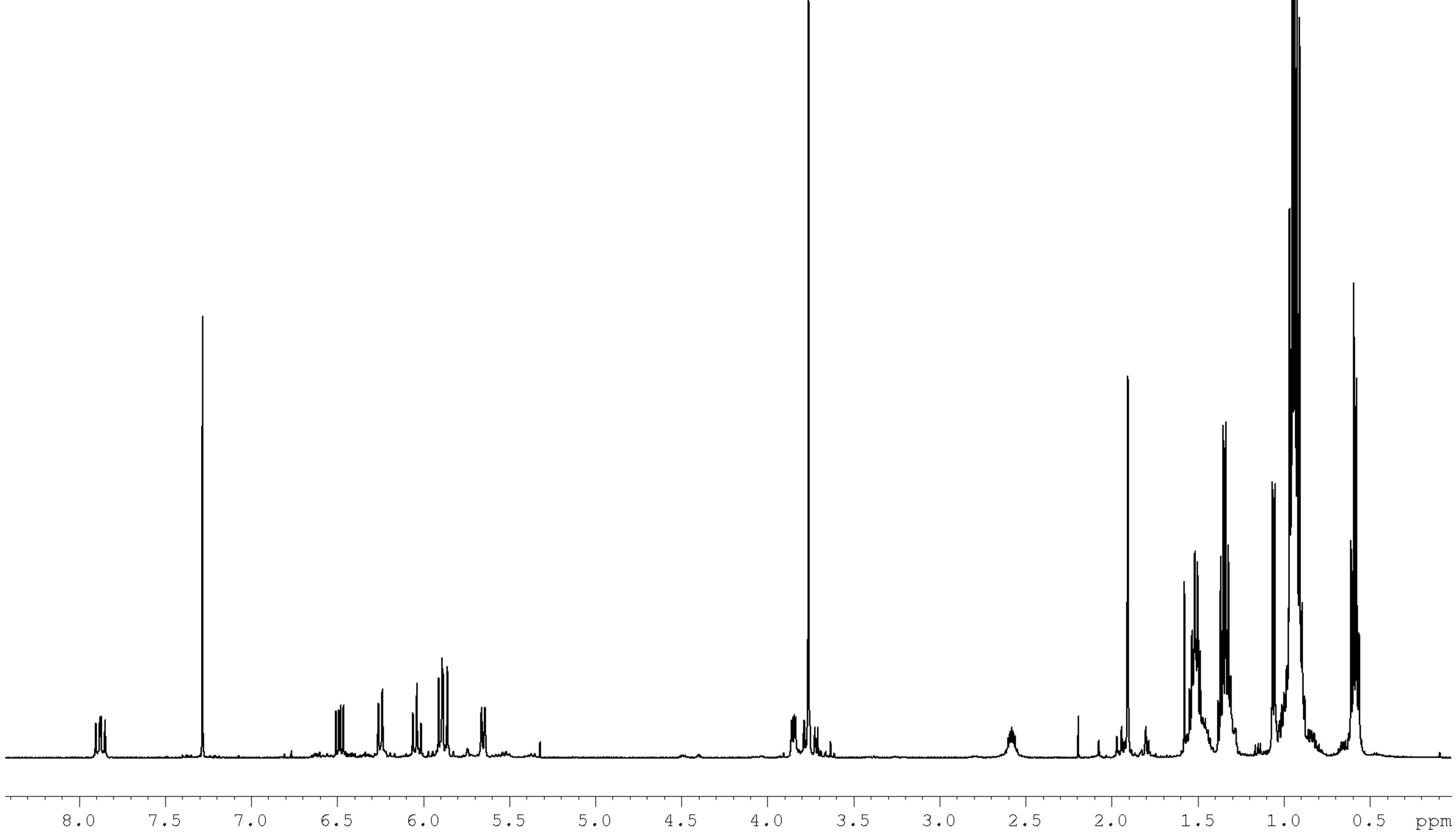

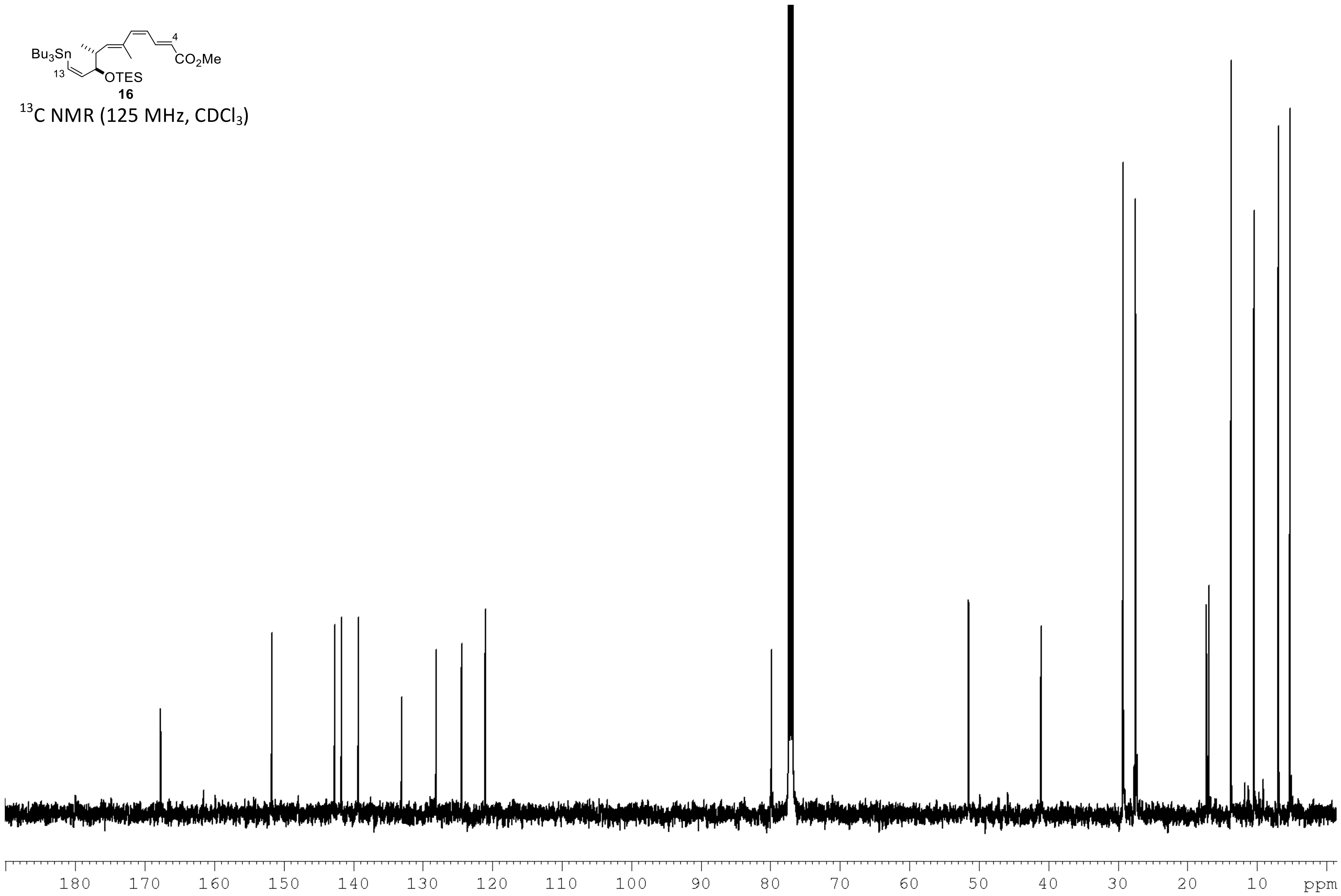

16

^{13}C NMR (125 MHz, $CDCl_3$)

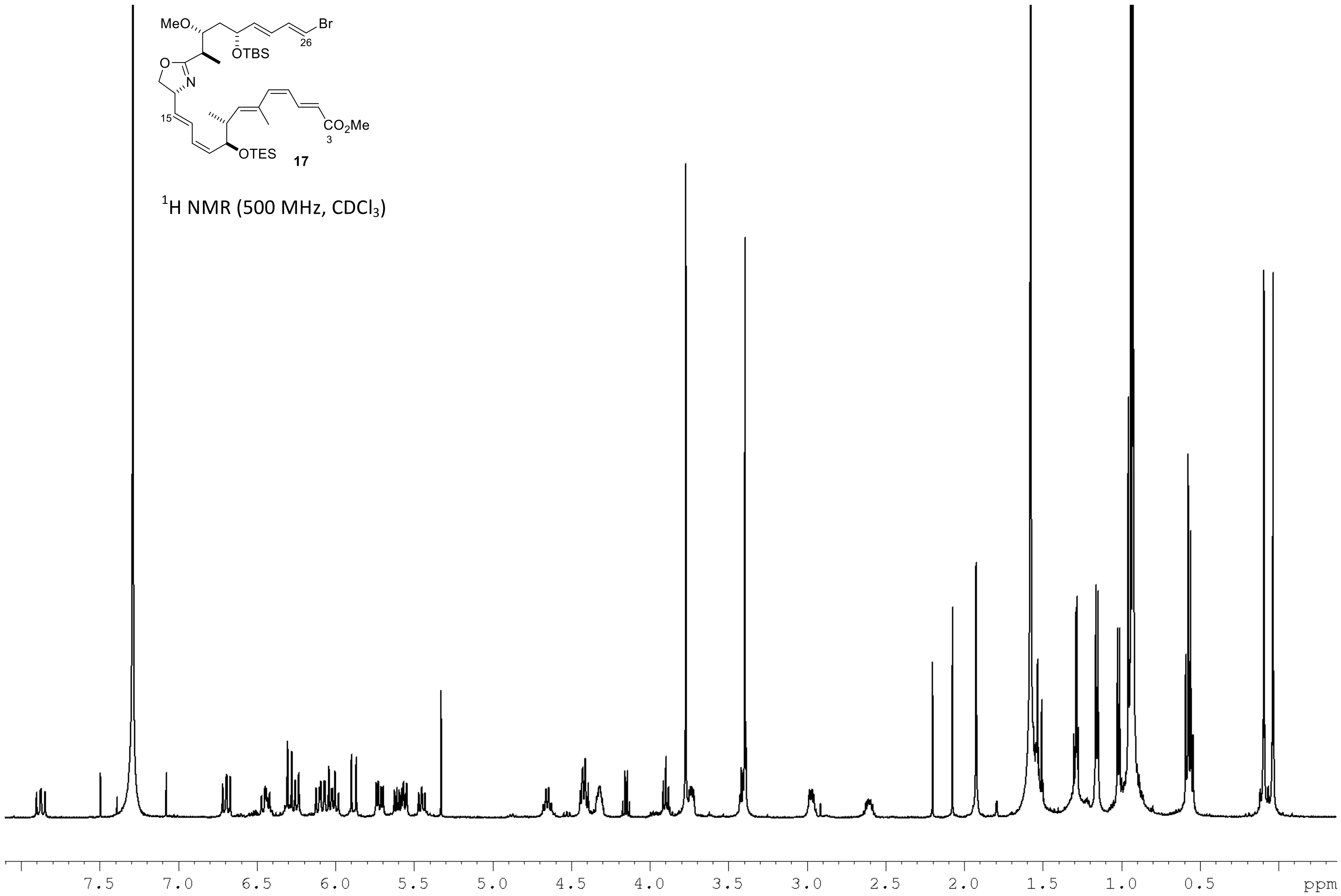
MeO
Br
26
OTBS
O
N
15
CO2Me
3
OTES
17
1H NMR (500 MHz, CDCl3)
7.5
7.0
6.5
6.0
5.5
5.0
4.5
4.0
3.5
3.0
2.5
2.0
1.5
1.0
0.5
ppm

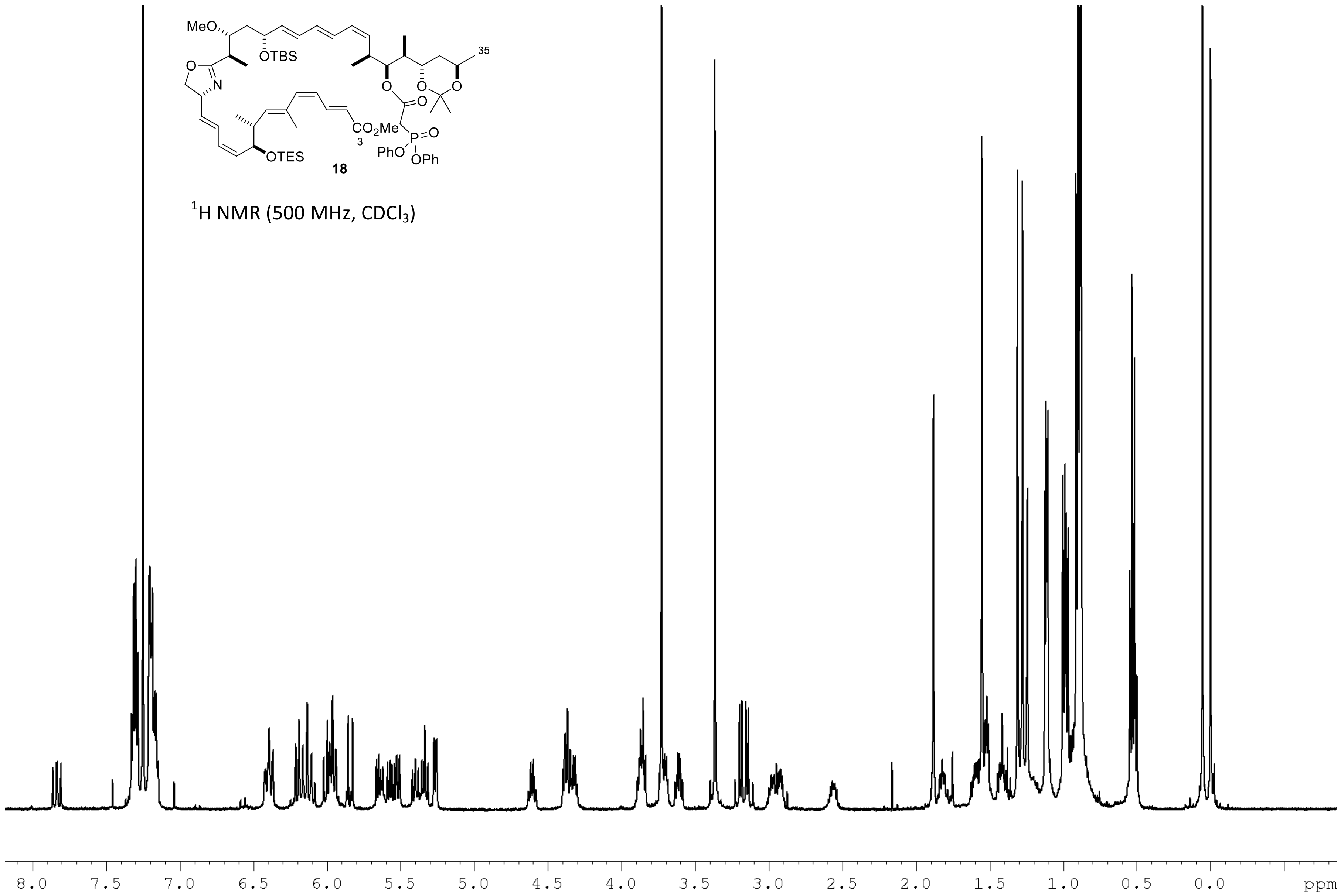

18

^{1}H NMR (500 MHz, $CDCl_3$)

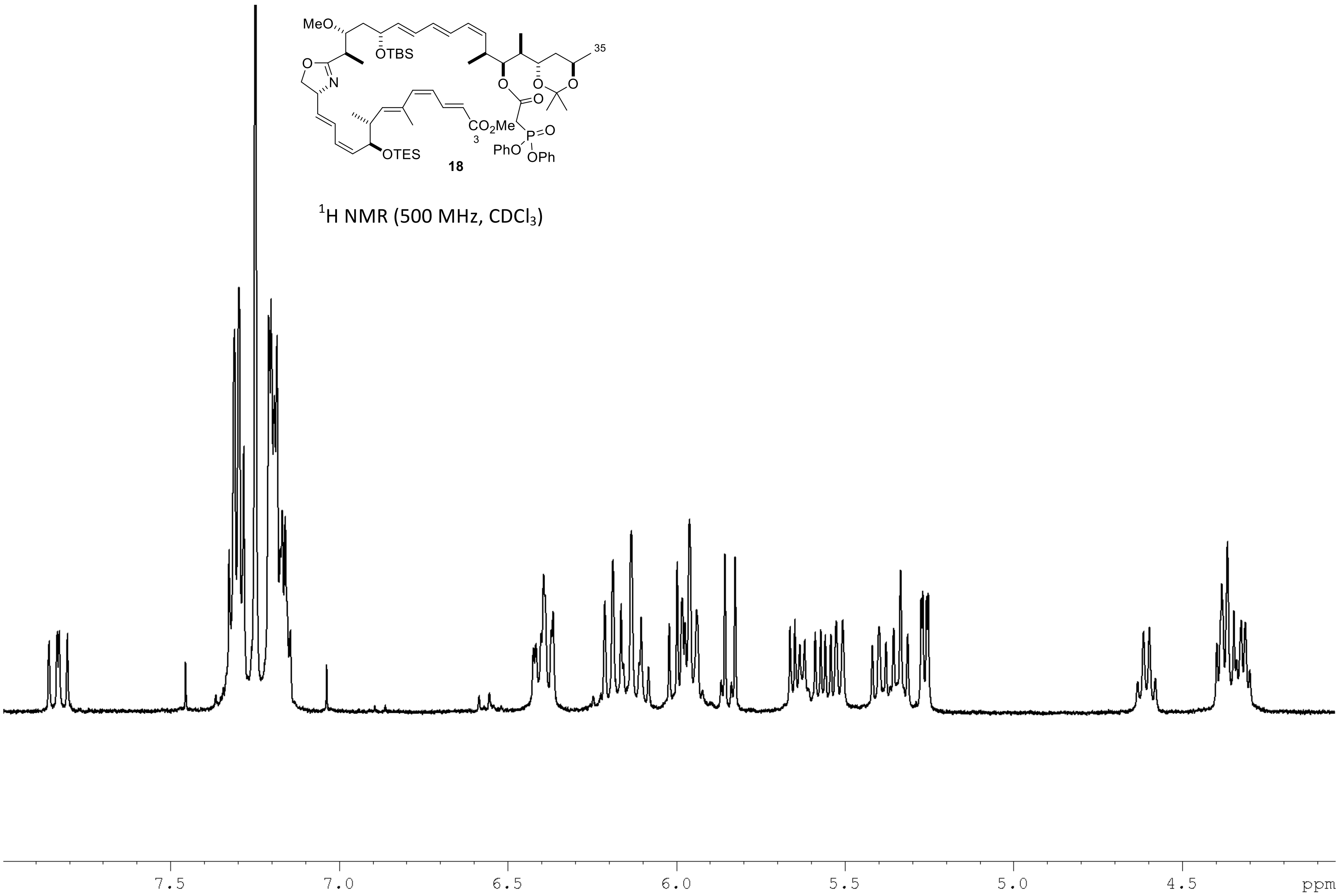
MeO
OTBS
OTES
CO2Me
3
O
N
O
O
O
O
P=O
PhO
OPh
35
18
1H NMR (500 MHz, CDCl3)
7.5
7.0
6.5
6.0
5.5
5.0
4.5
ppm

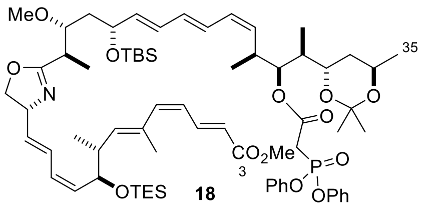

18

^{13}C NMR (125 MHz, $CDCl_3$)

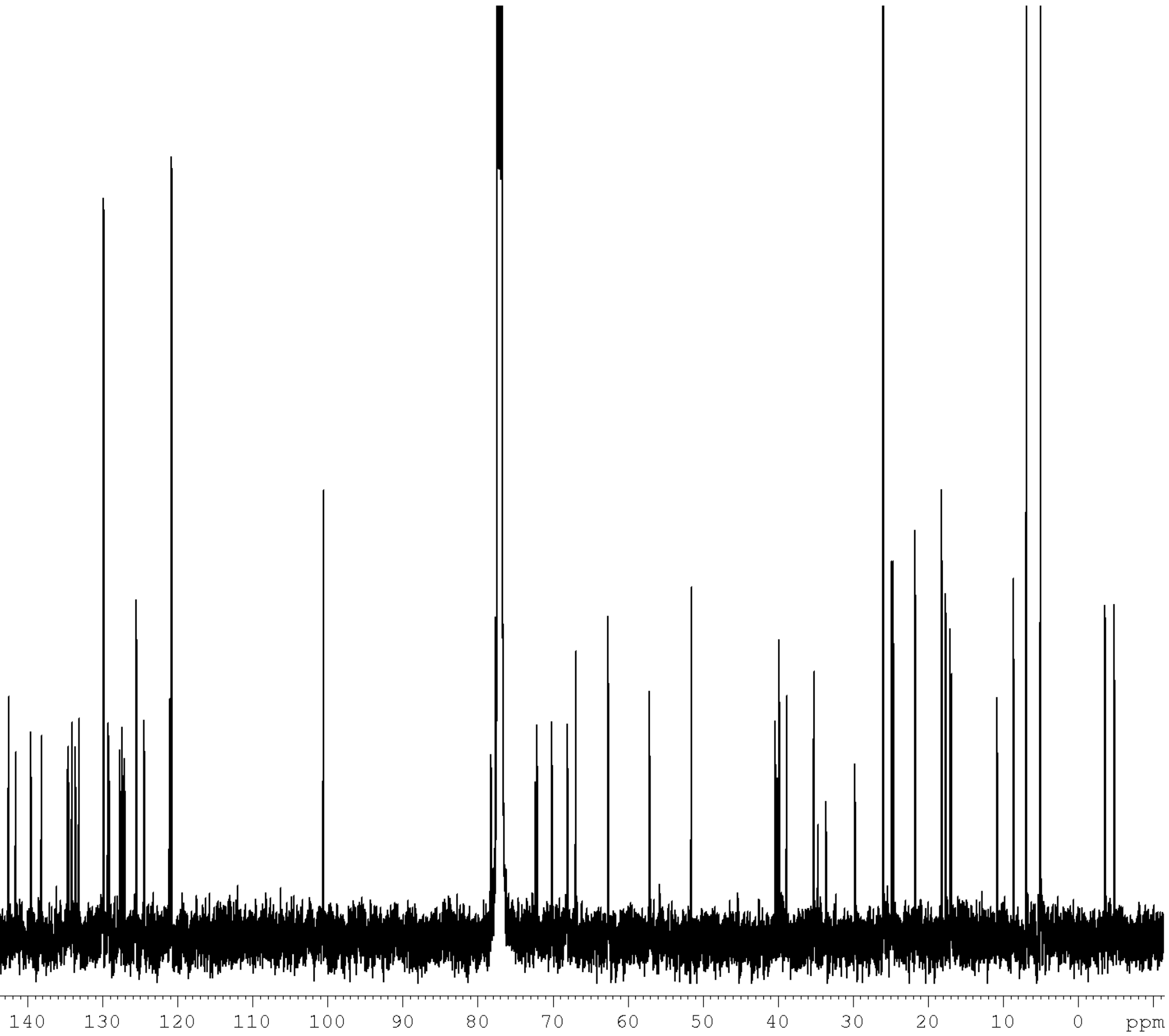

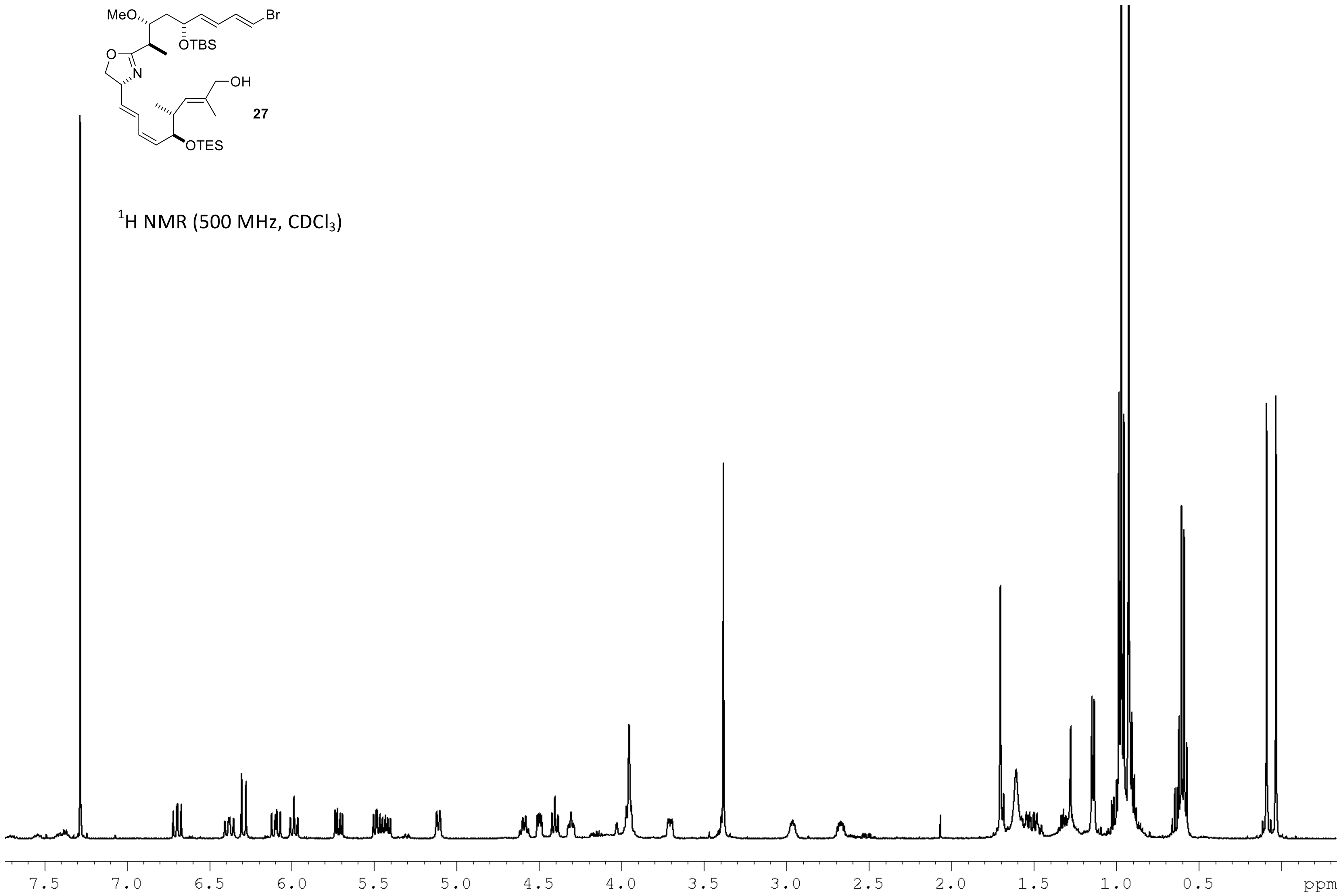
MeO
OTBS
Br
O
N
OH
OTES
27
^{1}H NMR (500 MHz, $CDCl_3$)
7.5
7.0
6.5
6.0
5.5
5.0
4.5
4.0
3.5
3.0
2.5
2.0
1.5
1.0
0.5
ppm

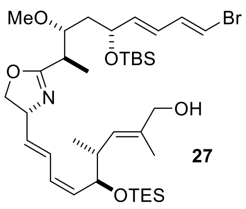

27

^{13}C NMR (125 MHz, $CDCl_3$)

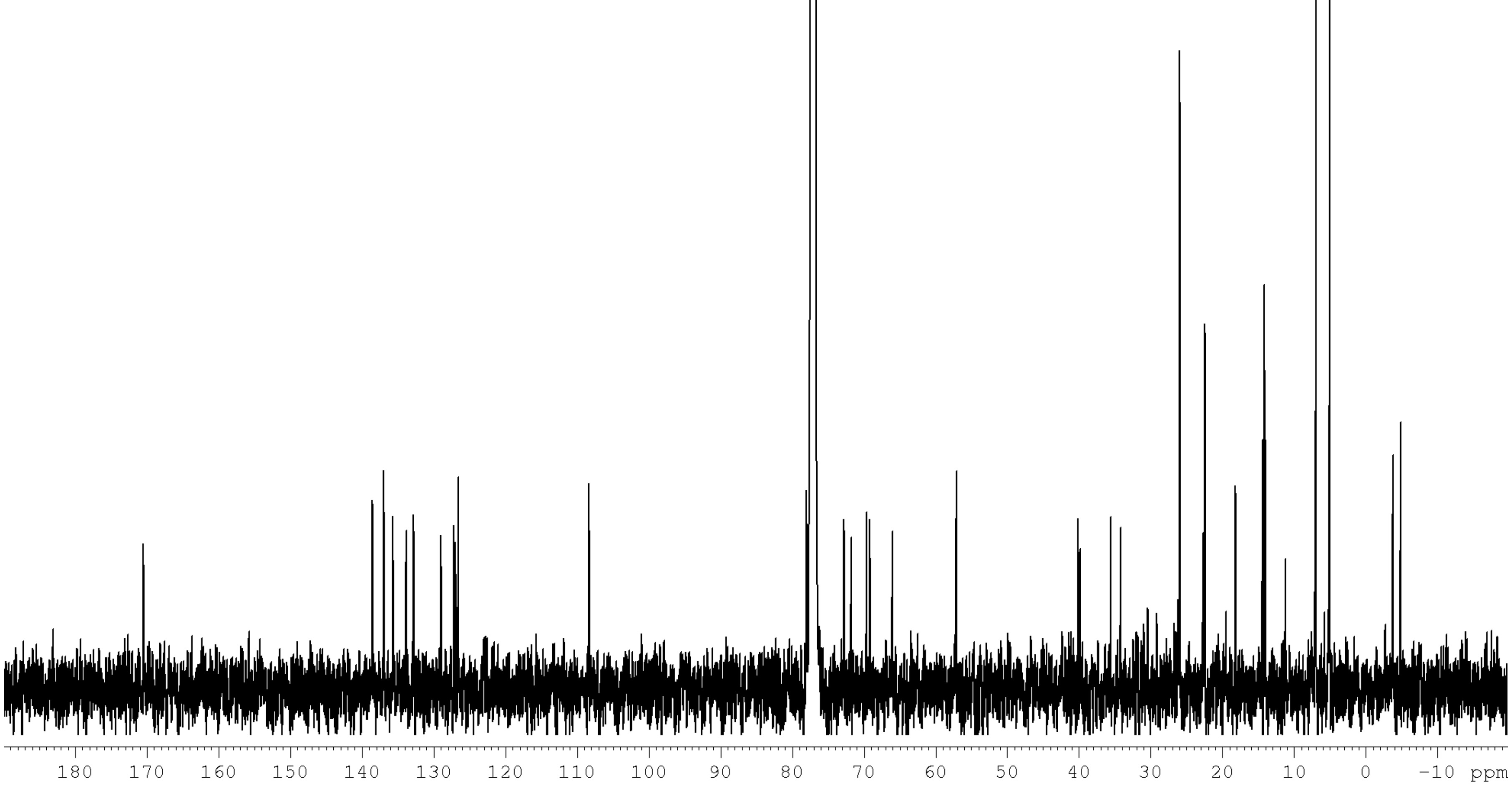

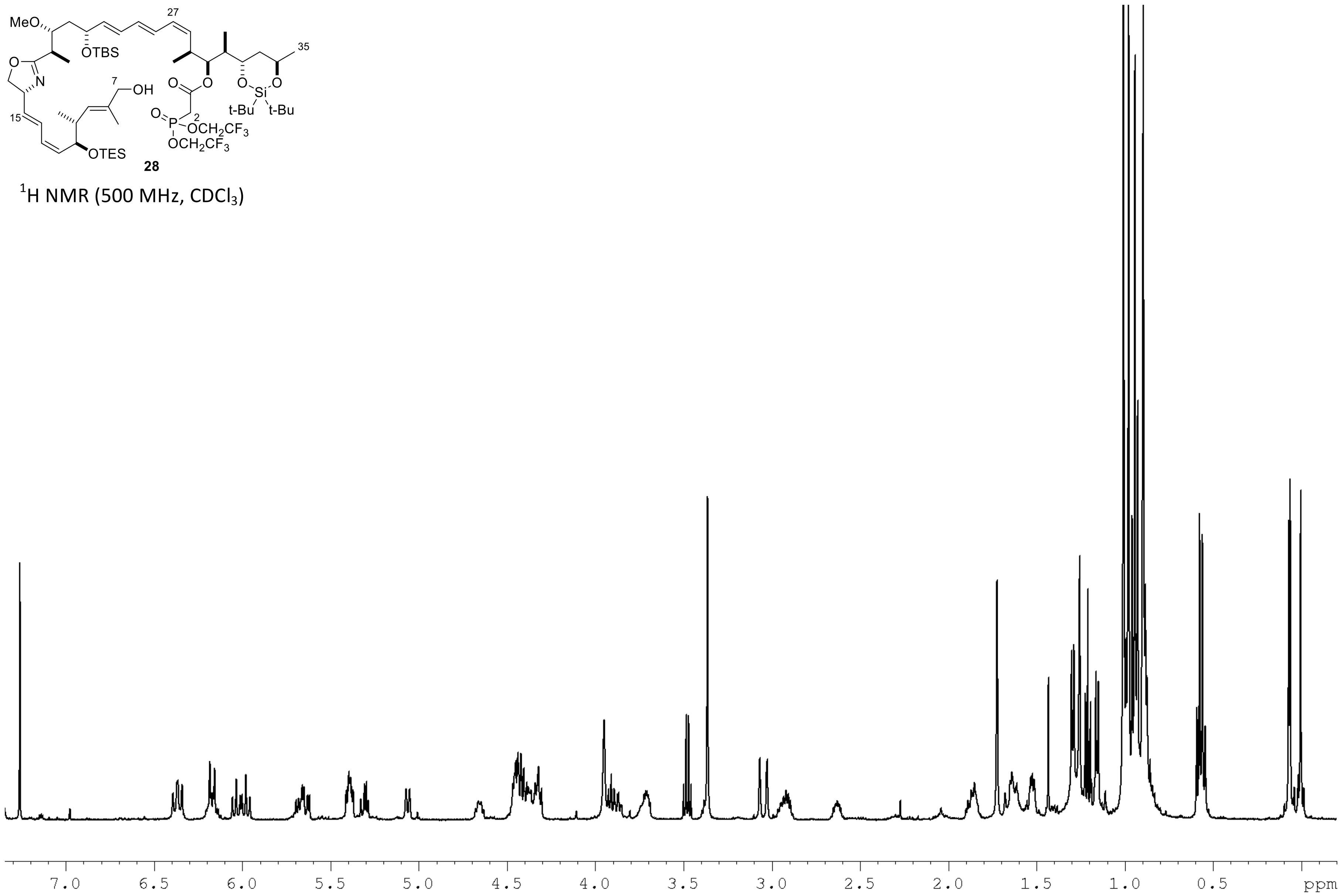
MeO
OTBS
27
35
O
N
7
OH
O
O
O
Si
t-Bu
t-Bu
15
P
2
OCH2CF3
OCH2CF3
OTES
28
1H NMR (500 MHz, CDCl3)
7.0
6.5
6.0
5.5
5.0
4.5
4.0
3.5
3.0
2.5
2.0
1.5
1.0
0.5
ppm

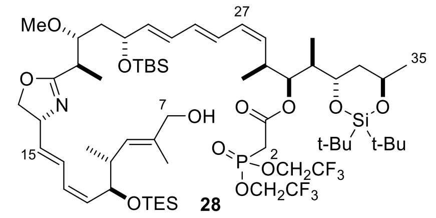

^{13}C NMR (125 MHz, $CDCl_3$)

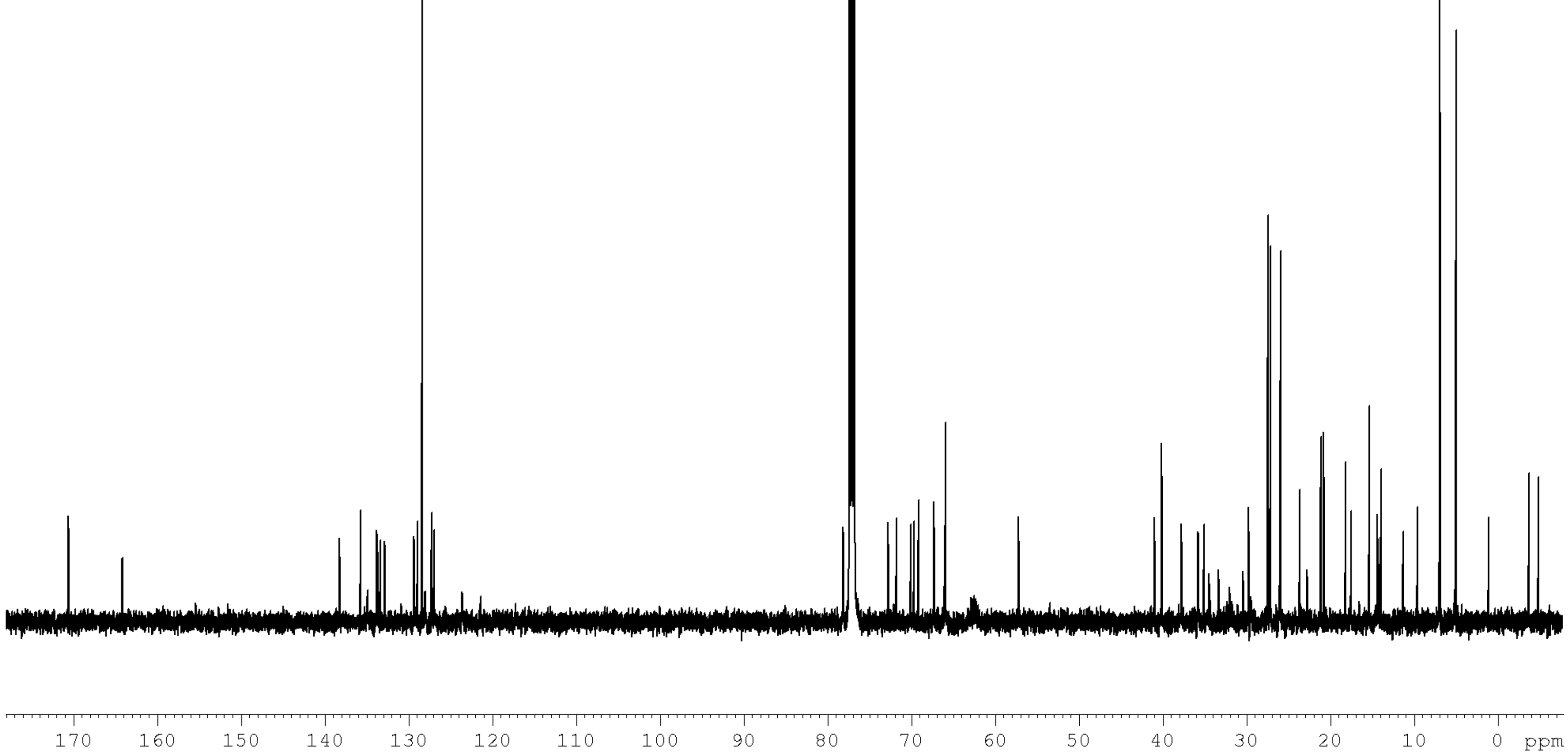

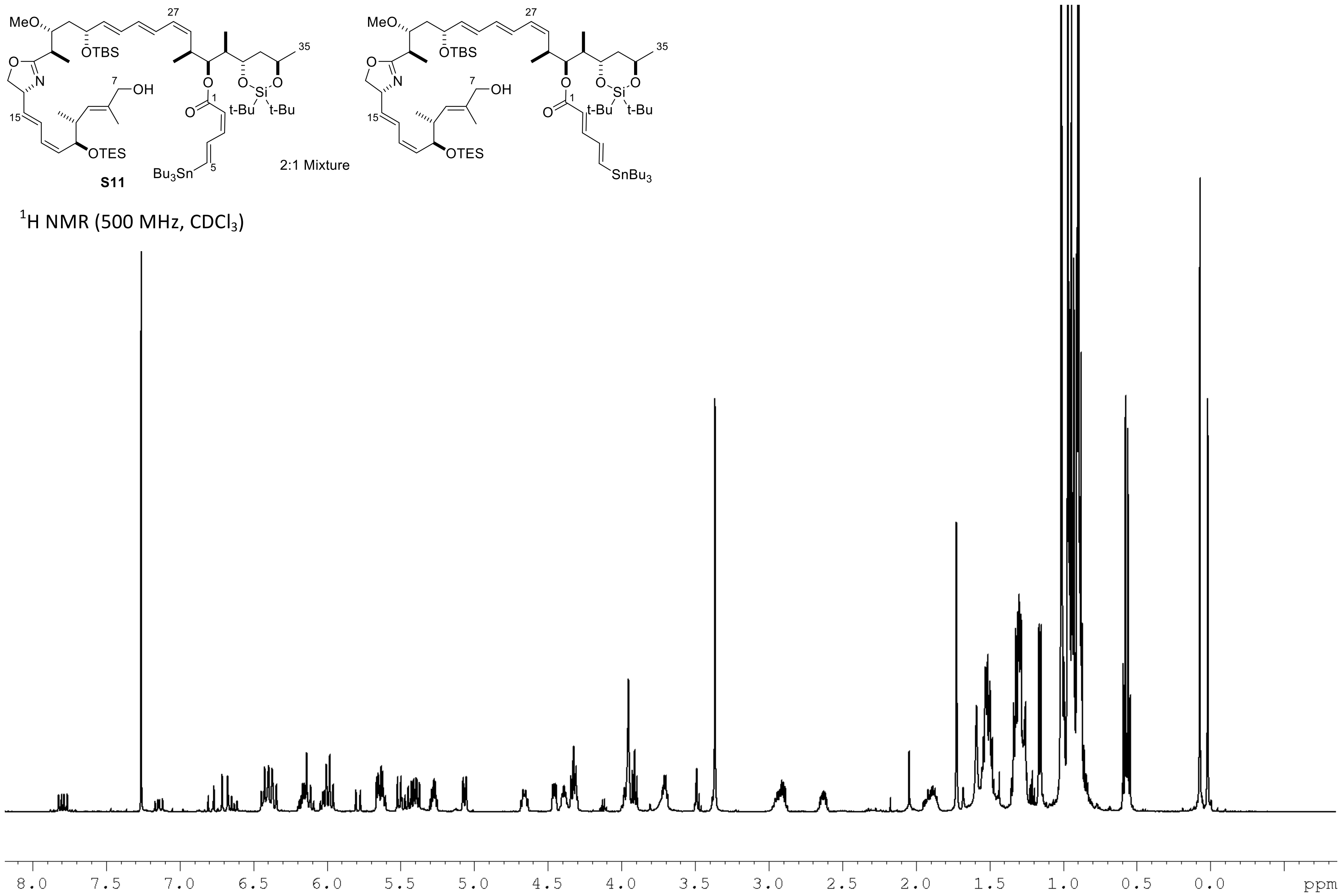

S11

2:1 Mixture

^{1}H NMR (500 MHz, $CDCl_3$)

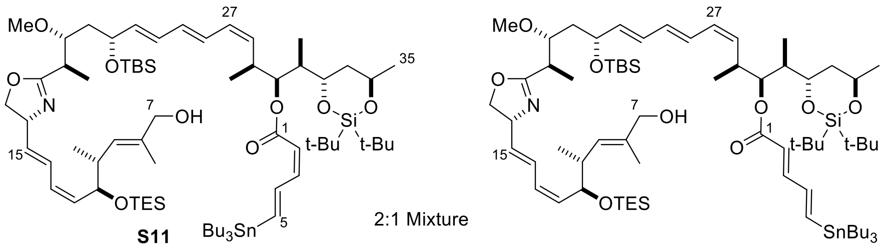

S11

2:1 Mixture

^{13}C NMR (125 MHz, $CDCl_3$)

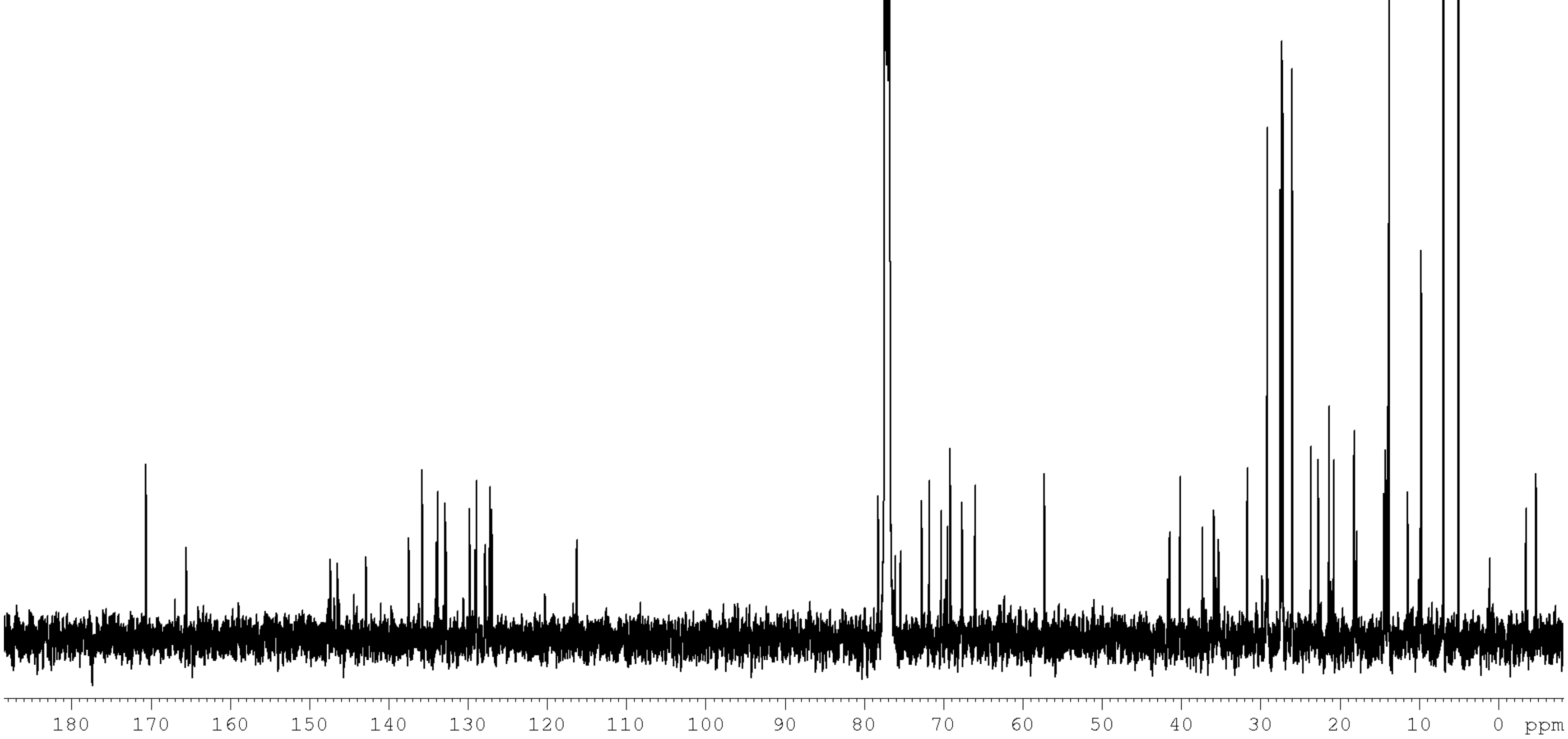

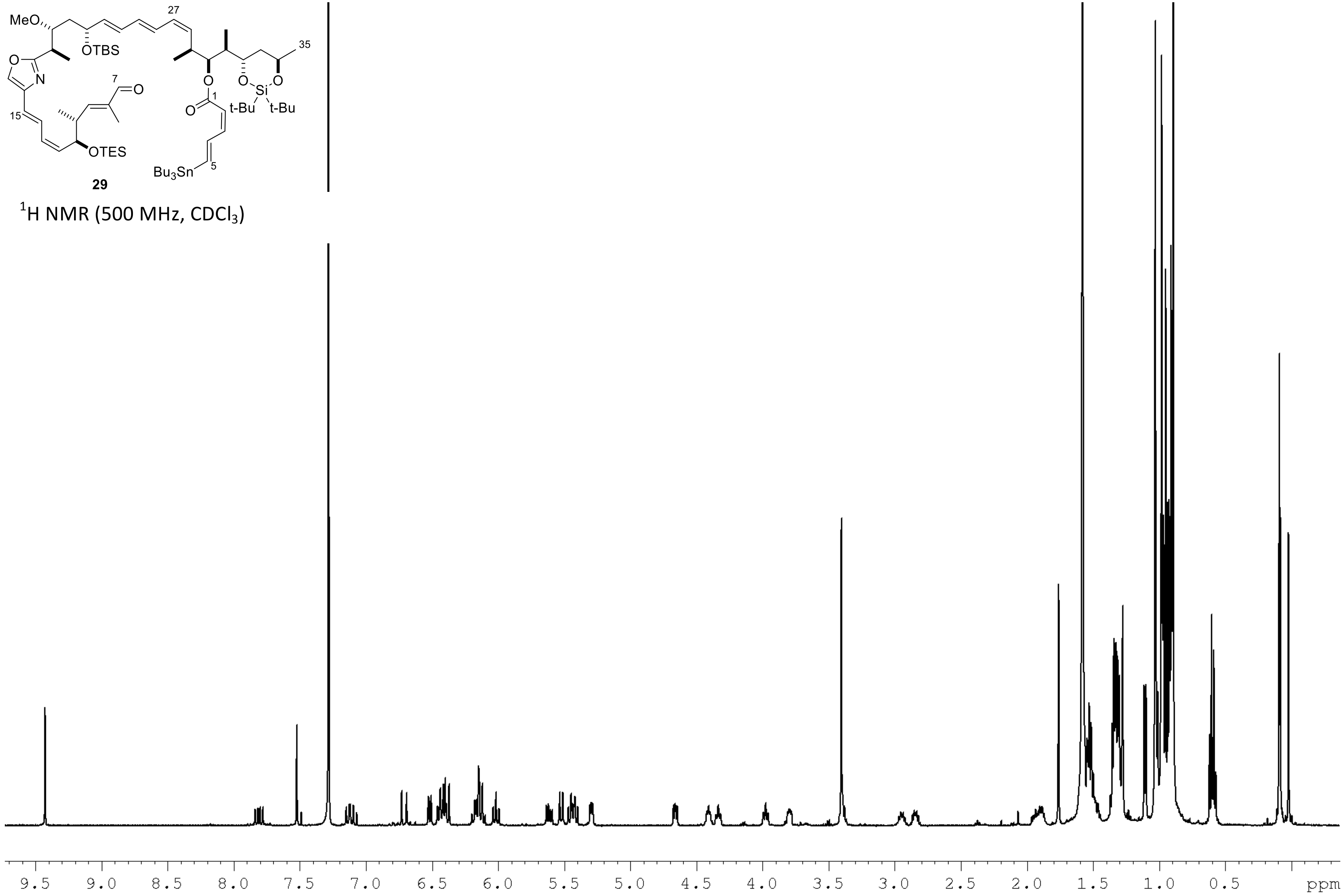
MeO
OTBS
27
35
O
N
7
O
1
t-Bu
Si
t-Bu
15
OTES
Bu3Sn
5
29
1H NMR (500 MHz, CDCl3)
9.5 9.0 8.5 8.0 7.5 7.0 6.5 6.0 5.5 5.0 4.5 4.0 3.5 3.0 2.5 2.0 1.5 1.0 0.5 ppm

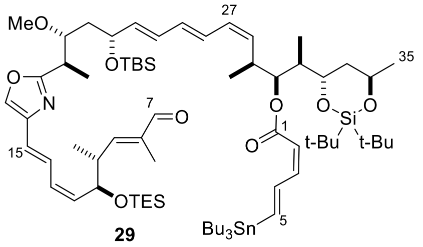

^{13}C NMR (125 MHz, $CDCl_3$)

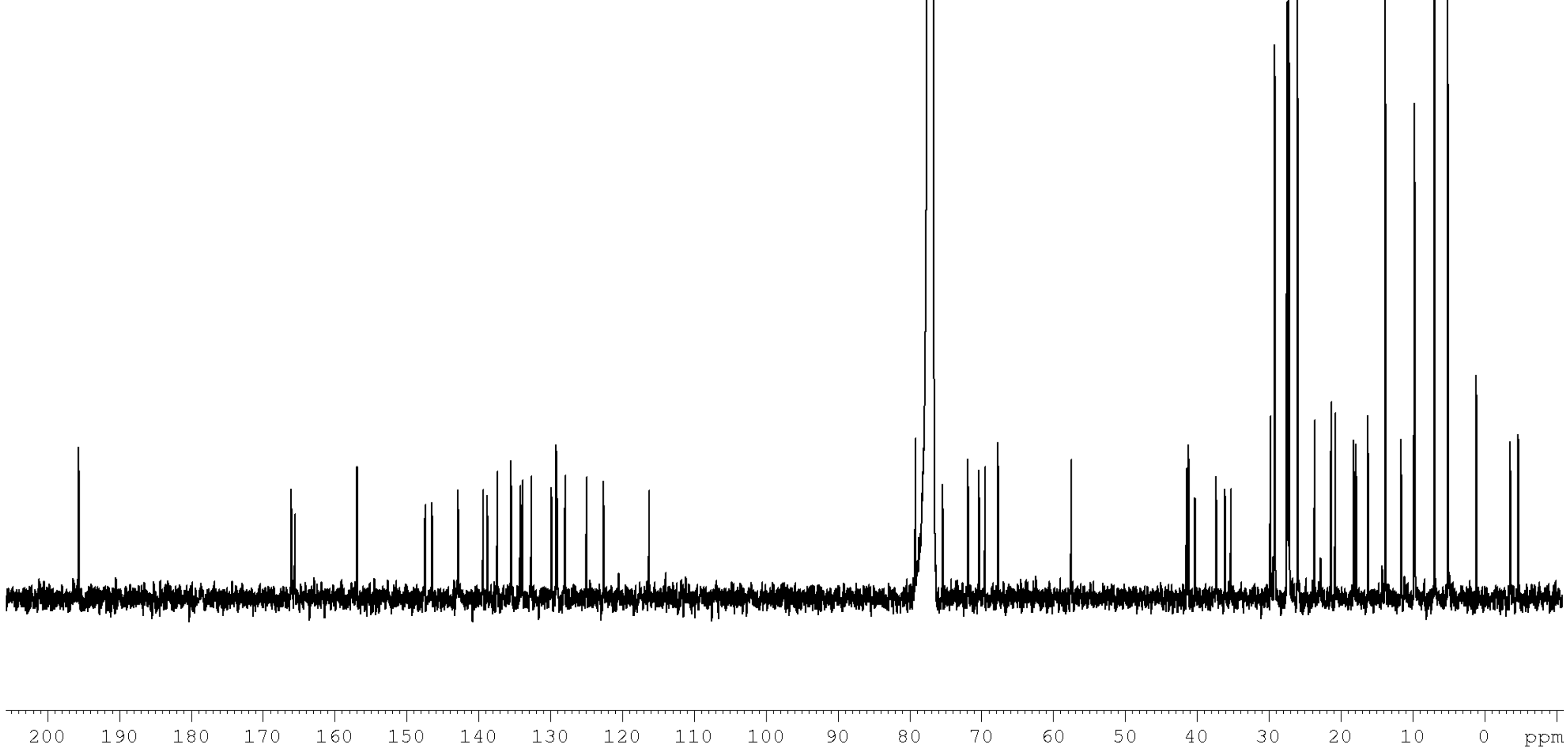

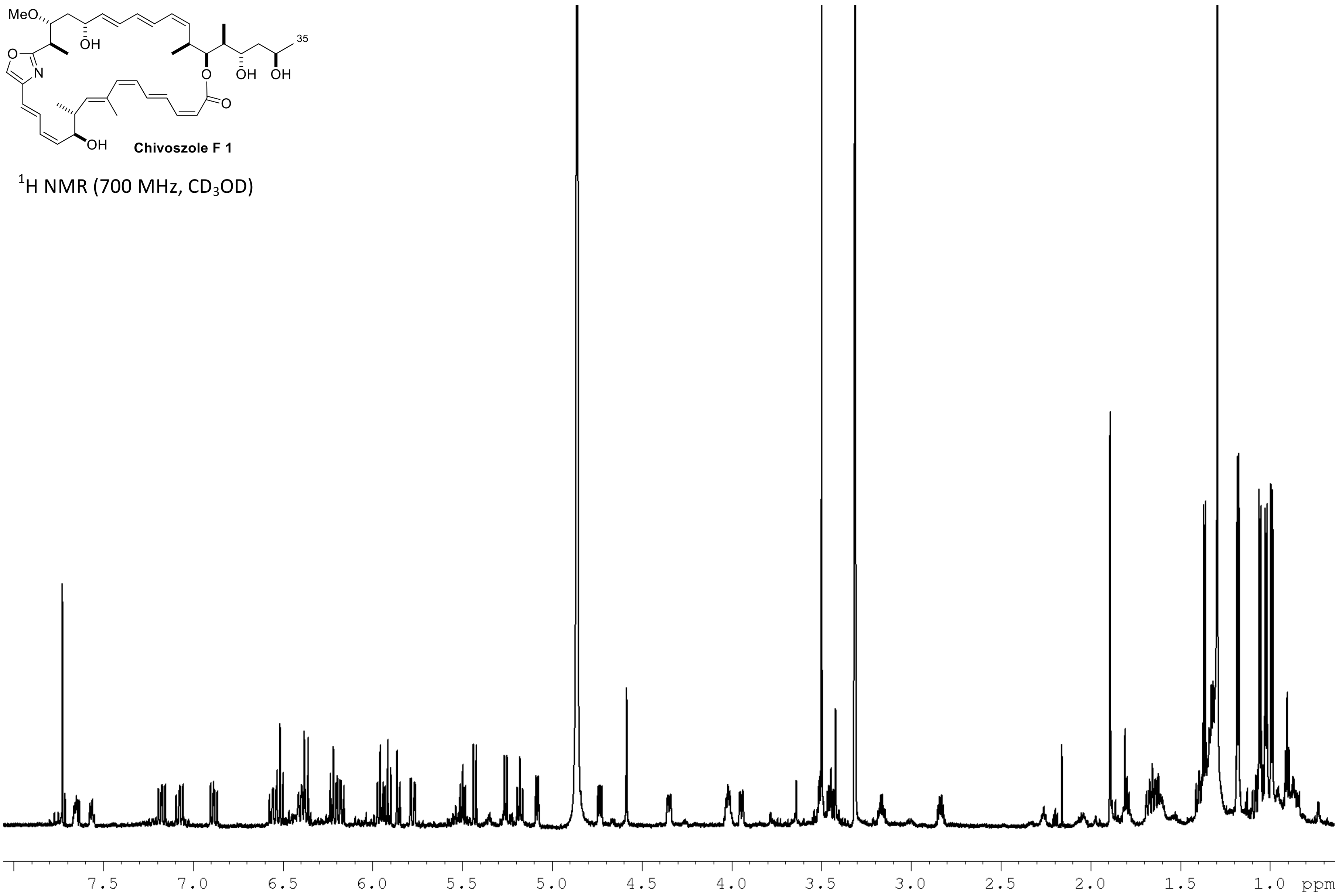
MeO
OH
O
N
35
OH OH
O
O
OH
Chivoszole F 1
^{1}H NMR (700 MHz, CD_3OD)
7.5 7.0 6.5 6.0 5.5 5.0 4.5 4.0 3.5 3.0 2.5 2.0 1.5 1.0 ppm

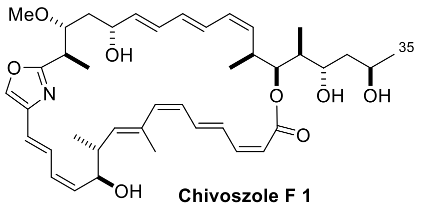

Chivoszole F 1

^{1}H NMR (700 MHz, CD$_3$OD)

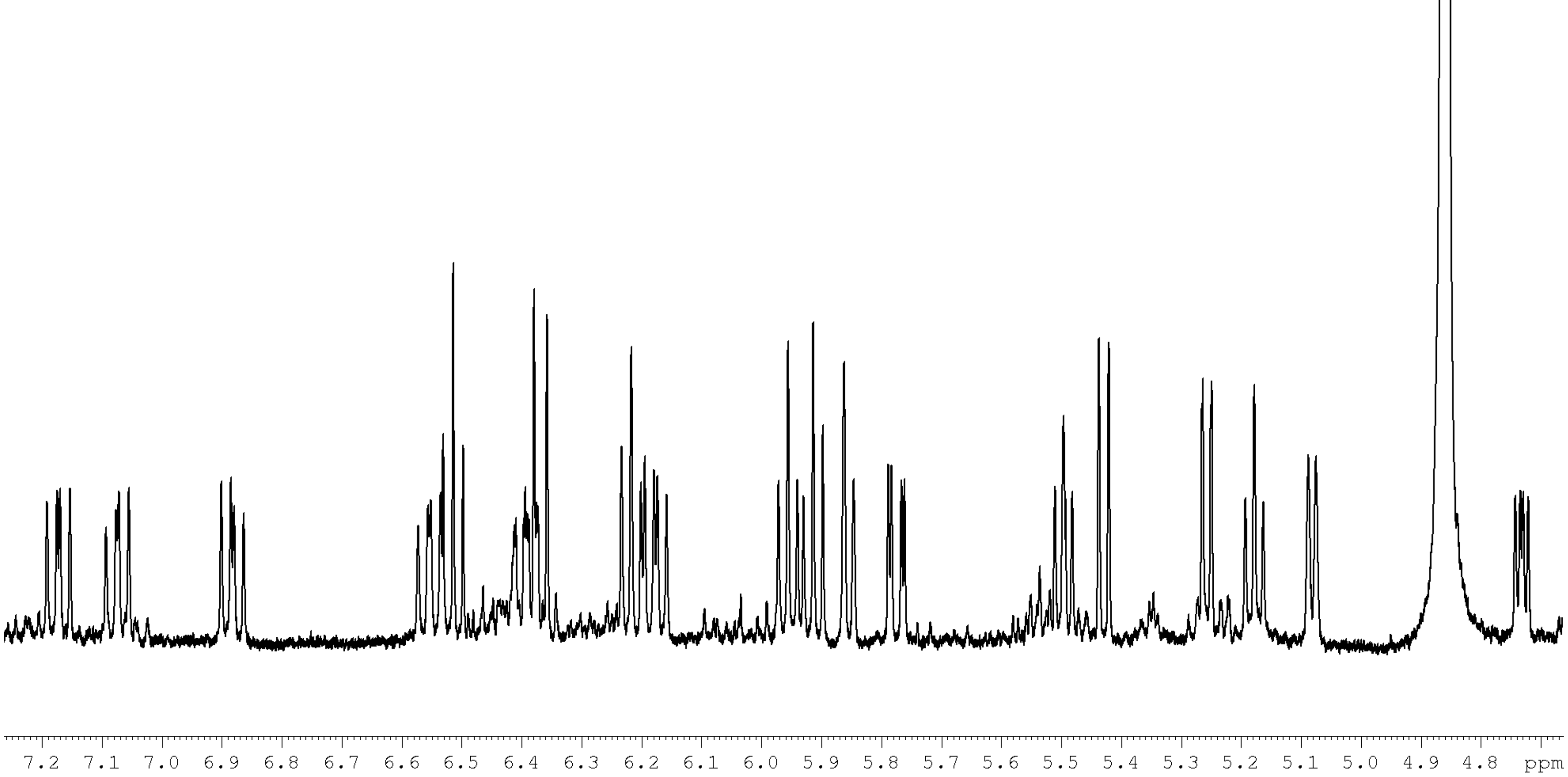

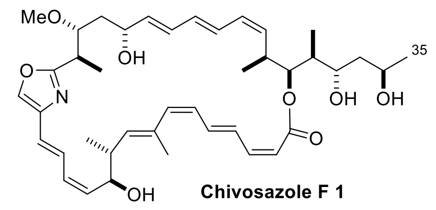

Chivosazole F 1

^{13}C NMR (175 MHz, CD_3OD)

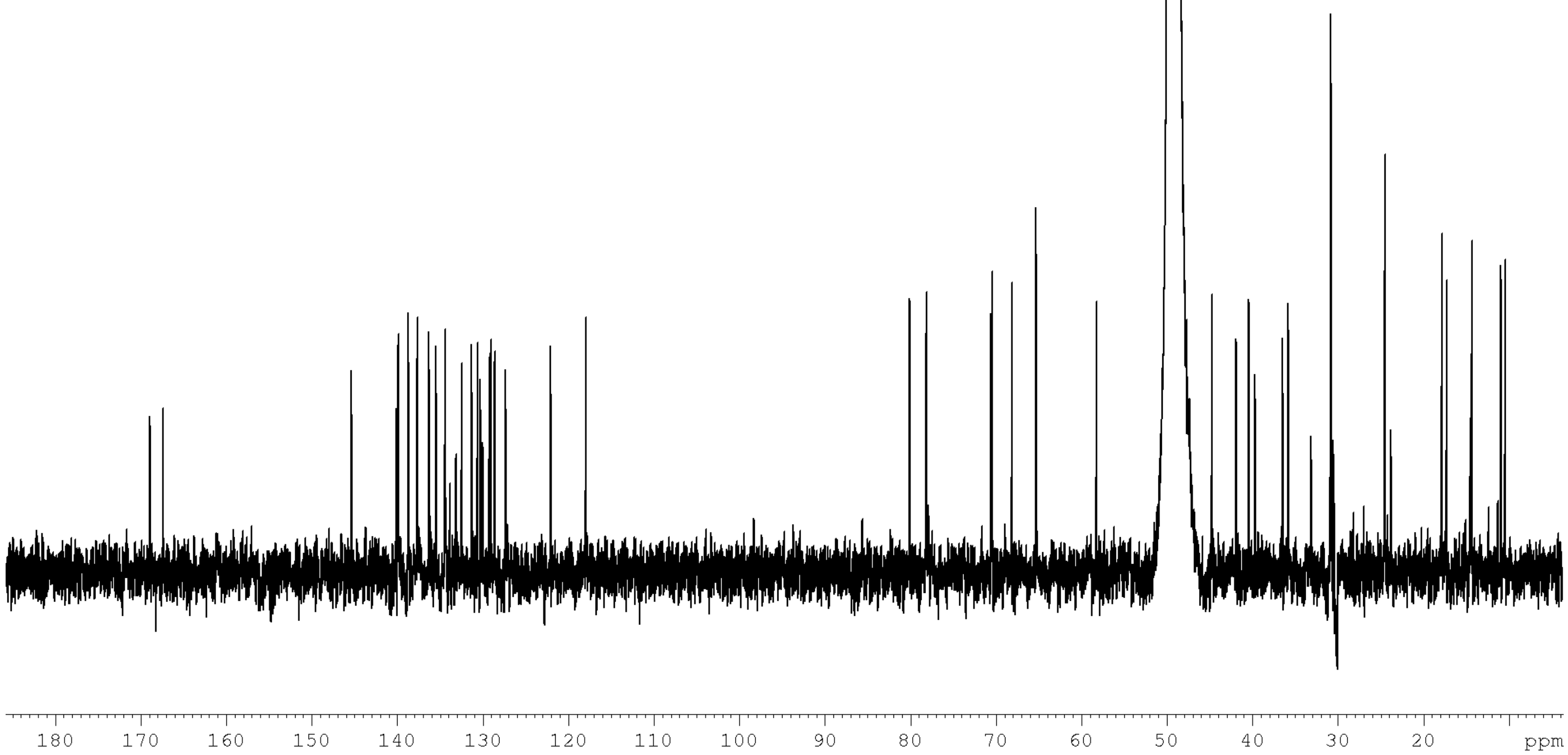

Supplement: Supplementary file 1 — Supplementary [file ANIE-56-645-s001.pdf]
